# A Stretchable, Mechanically‐Interlocked Polyrotaxane Hydrogel for Wearable Motion and Electrophysiological Monitoring

**DOI:** 10.1002/advs.75205

**Published:** 2026-04-10

**Authors:** Hao‐Zheng Huang, Yu‐Tao Zheng, Feng Chen, Jing‐Yi Li, Wen‐Hui Zhang, Liang Ding, Zhegang Huang, Wei Jiang, Li‐Ping Huang, Xing‐Can Shen

**Affiliations:** ^1^ State Key Laboratory for Chemistry and Molecular Engineering of Medicinal Resources School of Chemistry and Pharmaceutical Sciences Guangxi Normal University Guilin China; ^2^ Department of Chemistry Southern University of Science and Technology Shenzhen China; ^3^ PCFM and LIFM Lab School of Chemistry Sun Yat‐sen University Guangzhou China

**Keywords:** allyl‐functionalized naphthotube, polyrotaxanes hydrogel, wearable bioelectronics

## Abstract

Many conductive hydrogels have been developed for wearable electronics; however, it remains a challenge to achieve simultaneous mechanical robustness, stable electrical properties, and tissue–compliant interfaces. Herein, we report a mechanically interlocked polyrotaxane hydrogel prepared via one‐pot photopolymerization. The designed network integrates the energy‐dissipative “pulley effect” of sliding macrocycles with a stable covalent network. The resulting hydrogel exhibits skin‐like softness (modulus ∼8.5 kPa), ultrahigh stretchability (2450%), strong adhesion, and high ionic conductivity (7.46 mS/cm). It functions as a durable strain sensor with a broad sensing range and stable cyclic performance over 10 000 cycles. As an epidermal electrode, it acquires high‑fidelity electrocardiogram (ECG) and electromyogram (EMG) signals with a superior signal‑to‑noise ratio (>42 dB), even during motion, and maintains high signal quality over 24 h. Furthermore, a wearable five‑sensor array demonstrates its capability for real‑time gesture recognition and wireless robotic control. This work provides a robust and multifunctional material platform for advanced wearable bioelectronics.

## Graphical abstract

A mechanically interlocked polyrotaxane hydrogel integrates sliding macrocycles within a covalent network, yielding skin‑like softness, high stretchability, robust adhesion, and stable ionic conductivity. This multifunctional interface enables simultaneous high‑fidelity monitoring of human motion and epidermal electrophysiological signals (ECG/EMG), showcasing its potential for advanced wearable healthcare and human‑‐machine interaction.

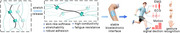

## 1 Introduction

The proliferation of wearable electronics has markedly transformed personalized healthcare and human–machine interaction [1, 2]. By enabling real‐time monitoring of human motion [3, 4] and electrophysiological signals [5, 6], these devices are pivotal for continuous health assessment [7], rehabilitation therapy [8], and advanced intelligent interfaces [9]. Conventional wearable systems, predominantly fabricated from metals, semiconductors, and carbon‐based materials, exhibit high electrical conductivity and sensitivity [10, 11, 12]. However, their inherent rigidity and mechanical mismatch with biological tissues lead to poor interfacial adaptation, compromising signal fidelity and long‐term user comfort [13]. To bridge this gap, a spectrum of soft, flexible conductive materials has been engineered to emulate the mechanical and functional properties of human tissue [14]. These materials achieve conformal contact with curvilinear surfaces and efficiently transduce external stimuli and intrinsic biological activities into electrical signals, thereby addressing the key limitations of traditional rigid constructs [15, 16, 17]. Notably, conductive hydrogels have garnered increasing research interest due to their skin‐like attributes, including comparable Young's modulus, softness, and tunable conductivity, rendering them promising candidates for electronic skin [18], energy storage devices [19, 20], soft actuators [21, 22], bioelectrodes [23], and human–machine interfaces [24]. Nevertheless, the practical deployment of hydrogel‐based devices is often hindered by their susceptibility to fatigue fracture under cyclic loading, which induces unstable electrical outputs [25]. This drawback primarily stems from inhomogeneous network structures and inefficient stress dissipation mechanisms [26]. Consequently, fabricating conductive hydrogels that integrate robust mechanical endurance with stable electrical performance under sustained operational conditions remains a significant challenge.

Polyrotaxanes represent a distinctive class of supramolecular polymers characterized by mechanically interlocked architectures, where macrocycles are threaded onto linear polymer chains via reversible host–guest interactions [27, 28]. Pioneered by Ito et al., the defining feature of these systems is the “pulley effect”, a unique molecular motion within their dynamic networks [29]. This effect enables movable cross‐links to efficiently dissipate mechanical energy, thereby endowing the materials with exceptional fracture resistance and toughness [30, 31, 32]. Owing to these remarkable properties, polyrotaxanes have attracted extensive research attention and have been successfully incorporated into diverse material platforms, including polymer elastomers [33, 34], solid electrolytes [35, 36], supercapacitors [37], and bioelectronics [38, 39]. For instance, Choi et al. enhanced the elasticity and cycling stability of a conventional polyacrylic acid binder for silicon anodes via polyrotaxane modification [40, 41, 42]. Similarly, Bao et al. leveraged topological supramolecular networks to fabricate stretchable light‐emitting devices and organic bioelectronic components [43]. These works underscore that polyrotaxane networks uniquely synergize the dynamic adaptability of supramolecular polymers with the structural stability of covalent frameworks, presenting a highly promising strategy for constructing conductive hydrogels with superior mechanical resilience. However, despite these significant advances, the rational design and fabrication of polyrotaxane‐based conductive hydrogels specifically tailored for sensing applications, such as high‐performance bioelectronic interface, remain largely unexplored. This knowledge gap can be attributed primarily to challenges including the relatively low aqueous solubility of many polyrotaxane derivatives and the synthetically complex end‐capping processes required for network stabilization [44, 45].

Building upon the foundational understanding of polyrotaxanes, our group has previously developed a series of endo‐functionalized macrocyclic hosts known as naphthotubes [46, 47]. Distinguished from conventional α‐cyclodextrin (α‐CD), the *endo*‐functionalized amide naphthotube exhibits superior binding affinity for poly(ethylene glycol) (PEG) chains, driven by synergistic shield hydrogen bonding and enhanced hydrophobic interactions [48, 49]. This robust molecular recognition facilitates the efficient threading of PEG to form stable pseudopolyrotaxane architectures. Capitalizing on this unique host–guest system, we initially fabricated a highly stretchable, shear‐induced transient hydrogel via metal‐ion coordination [48]. Subsequently, we engineered a mechanically trainable polyrotaxane hydrogel, where cyclic stretching training promoted the formation of integrated hydrogen‐bonding networks, leading to remarkable property enhancement [49].

**FIGURE 1:**
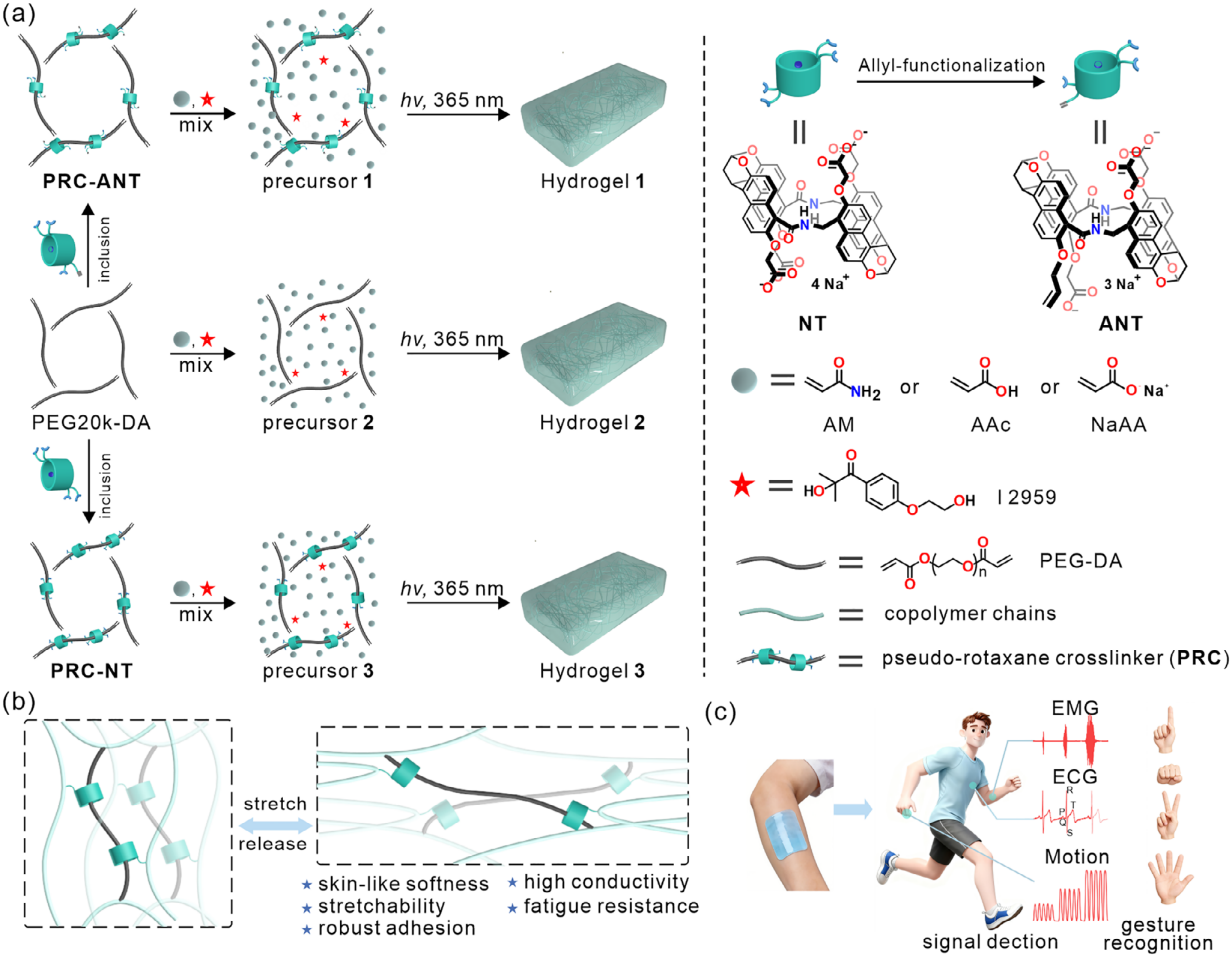
Design strategy, toughening mechanism, and proof‐of‐concept applications of the hydrogel. (a) Schematic of the fabrication process for hydrogel **1** and its control counterparts (hydrogels **2** and **3**). (b) Illustration of the dynamic sliding‐crosslink mechanism responsible for the superior toughness in hydrogel **1**. (c) Multifunctional applications of the hydrogel in sensing physiological signals (motion, ECG, EMG) and recognizing gestures.

Inspired by these promising precedents and to directly address the unmet challenge of reconciling mechanical robustness with electrical stability in hydrogels, we herein report the rational design of a novel, mechanically interlocked conductive hydrogel based on naphthotube polyrotaxanes, engineered specifically as a high‐performance bioelectronic interface. This hydrogel is synthesized through the covalent copolymerization of pseudorotaxane cross‐linkers (**PRC**) with functional vinyl monomers (Figure 1). Its architecture masterfully integrates the dynamic, energy‐dissipative “pulley effect” inherent to the supramolecular polyrotaxane skeleton with the structural permanence of a covalent polymer network. Consequently, the optimized hydrogel demonstrates an exceptional combination of properties: ultrahigh stretchability (fracture strain ∼2450%) and strength (fracture stress ∼110 kPa), a tissue‐compliant elastic modulus (∼8.5 kPa), outstanding fatigue resistance over 10 000 cycles, and robust adhesion to various substrates. The incorporation of mobile ions (H^+^ and Na^+^) renders the material highly conductive (7.46 mS/cm), while its low interfacial impedance ensures high‐fidelity biopotential acquisition. By leveraging this integrated suite of properties, we successfully demonstrate the assembly of the hydrogel into a multifunctional bioelectronic interface capable of reliably detecting a wide spectrum of signals, from large‐scale human joint motions to subtle epidermal electrophysiological activities, including electrocardiogram (ECG) and electromyogram (EMG) with superior signal to noise ratios (>42 dB). Furthermore, its functionality is extended toward interactive sensing applications. This work not only provides a viable material solution to a longstanding challenge but also establishes a new design paradigm for high‐performance, durable hydrogel‐based bioelectronics.

## 2 Results and Discussion

### 2.1 Fabrication of Allyl‐Functionalized Naphthotube and Hydrogels

The construction of robust, mechanically interlocked polyrotaxane networks necessitates the covalent integration of macrocycles into the polymer scaffold [50]. While our prior naphthotube‐based hydrogels relied on transient, non‐covalent cross‐links (e.g., metal‐coordination or hydrogen bonding), achieving permanent interlocking requires direct chemical grafting. To this end, we designed and synthesized a novel naphthotube derivative functionalized with allyl terminal groups (**S7**), following a modified synthetic route (Scheme S1) [51, 52]. The key intermediate, a bisnaphthalene cleft (**S3**) bearing both allyl and ester sidechains, was synthesized via a Friedel‐Crafts reaction between **S1** and **S2**, followed by intramolecular acetalization. The **S3** was further converted to diacid compounds **S5** by following the standard procedure reported earlier [46, 47]. Macrocyclization of **S5** with diamine **S6** under pseudo‐high‐dilution conditions afforded the target **S7** as a mixture of *syn*‐ and *anti*‐ isomers, both fully characterized by ^1^H/^13^C NMR and mass spectrometry. The configuration of **S7** was unambiguously assigned using 2D NMR spectroscopy and X‐ray crystallography (Supplementary Information). Given the established superior binding affinity of the *anti*‐isomer toward PEG chains, we selectively hydrolyzed this isomer to obtain the water‐soluble, mono‐allyl‐functionalized naphthotube (**ANT**).

The retention of host–guest recognition capability in **ANT** is paramount for forming pseudorotaxane cross‐linkers (**PRC**). To qualitatively confirm complexation, a 1:1 equimolar mixture of **ANT** and PEG_20k_‐DA was analyzed by ^1^H NMR spectroscopy. As shown in Figure S1, the characteristic proton signals of PEG exhibited pronounced upfield shifts upon mixing, consistent with their encapsulation within the hydrophobic cavity of **ANT** and analogous to the behavior observed with the classic carboxylate naphthotube (**NT**) [48, 49]. This confirms that the allyl functionalization does not impede the macrocycle's ability to thread onto PEG chains. Furthermore, to quantitatively compare the binding strengths, we performed ^1^H NMR titration experiments using a shorter, model guest molecule, oligo(ethylene glycol) diacrylate (TEG‐DA). These studies demonstrated that **ANT** possesses a stronger binding affinity for TEG‐DA than **NT** (Figure S2), indicating that the structural modification not only preserves but may actually enhance its PEG‐threading efficacy.

To translate this molecular recognition into a functional material, we engineered a conductive, mechanically interlocked hydrogel using a rationally selected monomer suite and the polymerizable **PRC‐ANT**. Acrylamide (AM) serves as the primary backbone monomer, providing a tunable, elastic network. Acrylic acid (AAc) and sodium acrylate (NaAA) are co‐monomerized to introduce mobile charge carriers (H^+^ and Na^+^ ions), which are essential for high ionic conductivity. Additionally, the carboxylate groups from AAc/NaAA impart robust substrate adhesion via electrostatic and hydrogen‐bonding interactions. Crucially, the allyl groups on **ANT** enable its covalent incorporation into the growing polymer network during radical polymerization, permanently locking the threaded PEG chains and creating the desired mechanically interlocked architecture. As schematically depicted in Figure 1, the hydrogel was fabricated via one‐pot process: first, **PRC‐ANT** cross‐linkers were self‐assembled in aqueous solution from **ANT** and PEG_20k_‐DA; subsequently, this mixture, along with AM, AAc, and NaAA, was photo‐polymerized using *Irgacure 2959* under UV irradiation, yielding the final conductive polyrotaxane hydrogel designed for wearable sensing applications.

### 2.2 Composition Optimization for Mechanical, Adhesive, and Conductive Properties

The performance of hydrogels in flexible wearable interfaces critically depends on achieving a balanced integration of mechanical robustness, stable adhesion, and high ionic conductivity [53]. To accomplish this integrated performance profile, we conducted a systematic, stepwise composition optimization.

Initial efforts focused on the mass ratio of the primary monomers, AM and AAc. As illustrated in Figure S3a,b, increasing the AAc content to 25 wt.% enhanced the tensile strength from 82 to 192 kPa, attributable to an increased physical cross‐linking density, albeit with a concomitant slight reduction in fracture strain. Beyond this point, further addition of AAc led to excessive cross‐linking, resulting in brittleness and compromised tensile properties [54]. In parallel, adhesion was assessed via 180° lap‐shear tests conducted independently on two representative substrates (Scheme S3): biological tissue (fresh porcine skin) and engineered surfaces (stainless steel). The results revealed a monotonic improvement in adhesive shear strength on both substrates as the AAc content increased up to 50 wt.% (Figure S3c,d). This enhancement stems from the abundant carboxyl groups of AAc, which foster intensive hydrogen bonding with skin and potential metal‐coordination with stainless steel [55]. Considering that robust adhesion is a prerequisite for stable signal acquisition by ensuring conformal contact, the AAc content was fixed at 50 wt.% to prioritize adhesive performance while maintaining acceptable mechanical integrity for subsequent optimization.

However, the ionic conductivity of this initial formulation was found to be insufficient for efficient signal transduction (Figure S4a). To address this, NaAA was introduced as a co‐monomer. The incorporation of NaAA, serving as a source of mobile Na^+^ ions, dramatically improved the ionic conductivity from 0.63 to 9.09 mS/cm as its amount increased from 0 to 30 mg (Figure S4b). Notably, at moderate concentrations (up to 15 mg), NaAA synergistically improved both tensile strength and adhesive shear strength. Beyond this optimal threshold, excessive NaAA induced a plasticizing effect, leading to a noticeable decline in both mechanical and adhesive properties (Figure S5) [54]. Consequently, a NaAA content of 15 mg was selected to optimally balance high conductivity with mechanical and adhesive integrity.

Finally, the influence of the **PRC‐ANT** concentration was systematically evaluated. Increasing the **PRC‐ANT** content from 2 mg to 10 mg enhanced the tensile strength from 42 to 230 kPa but reduced the fracture strain from 4210% to 1300%, with a concomitant increase in elastic modulus from 6 to 18 kPa (Figure S6a,b). This modulus range favorably matches that of human skin, promoting conformal contact [56]. Although the adhesive strength gradually declined with higher **PRC‐ANT** content, likely due to increased cross‐link density and modulus, the hydrogel maintained stable adhesion to both porcine skin and stainless steel (Figure S6c,d). Importantly, the ionic conductivity remained at a satisfactory level (5.03–8.19 mS/cm) across the entire **PRC‐ANT** range (Figure S7), ensuring reliable signal transmission. To achieve a superior overall balance, a **PRC‐ANT** content of 4 mg was identified as optimal, providing high strength (110 kPa) while maintaining excellent stretchability (2450%) and strong adhesion.

Through this iterative optimization process, an optimal hydrogel composition was identified: AM:AAc:NaAA = 50:50:15 (by mass ratio) with 4 mg of **PRC‐ANT**. This formulation exhibits an exceptional and balanced set of properties: a high tensile strength of 110 kPa, a large fracture strain of 2450%, a skin‐matched elastic modulus of 8.5 kPa, stable adhesive shear strengths of 92 kPa (porcine skin) and 49 kPa (stainless steel), and a high ionic conductivity of 7.46 mS/cm. These characteristics collectively ensure excellent compatibility with biological tissues and underscore the strong potential for advanced wearable applications. Therefore, this optimized hydrogel was employed in all subsequent investigations.

### 2.3 The Effect of the Mechanically Interlocked Polyrotaxane Networks

**FIGURE 2:**
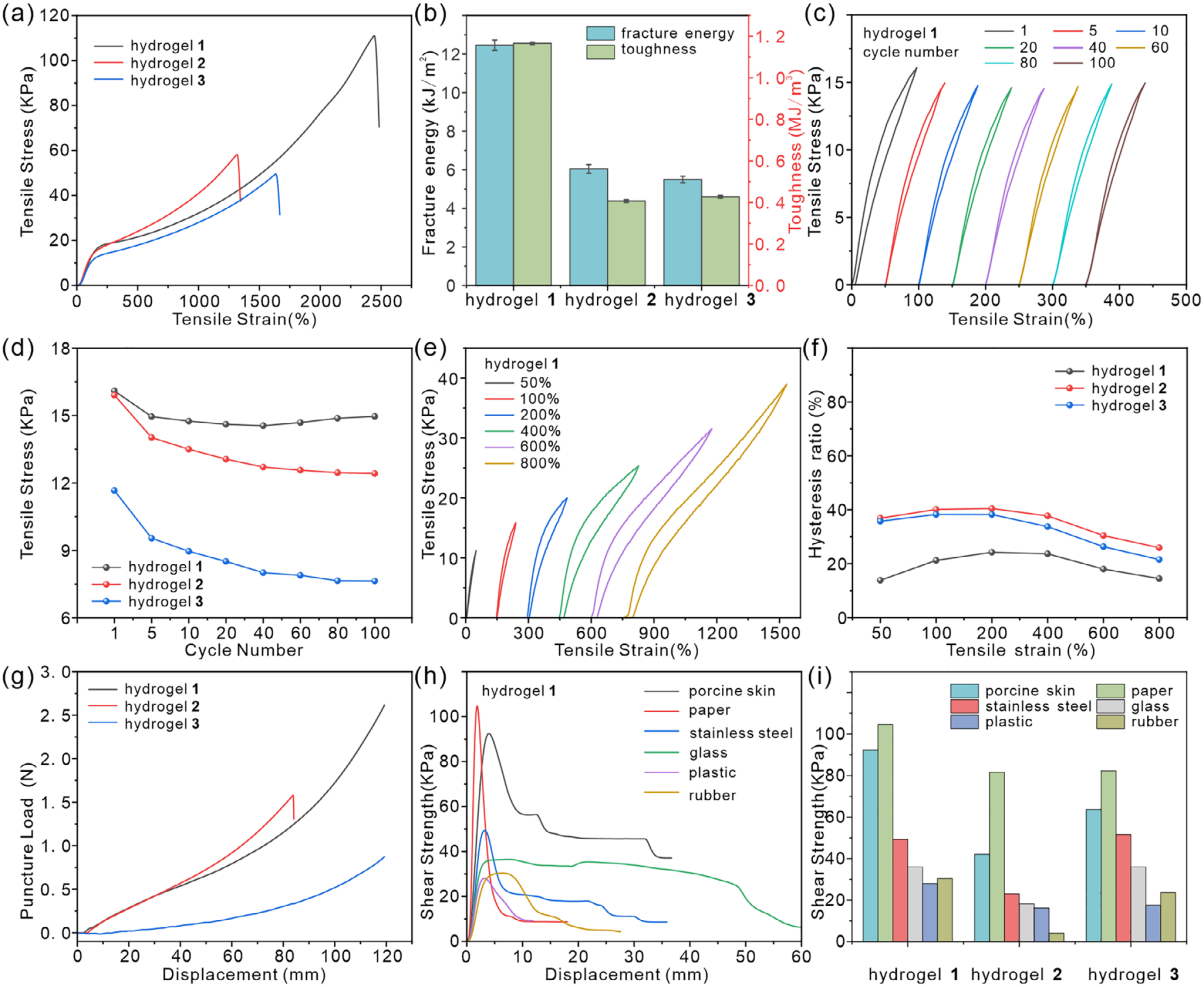
Comparative mechanical properties of the hydrogels **1**, **2**, and **3**. Representative tensile stress‐strain curves (a) and corresponding fracture energy and toughness (b) of the hydrogels **1**, **2**, and **3**. (c) Successive cyclic loading‐unloading curves of hydrogel **1** at 100% strain for 100 cycles. (d) The tensile stress change trends of hydrogels **1**, **2**, and **3** at 100% strain for 100 cycles. (e) Loading‐unloading tensile curves of hydrogel **1** at different strains from 50% to 800%. (f) Comparison of the hysteresis ratio change trends of hydrogel **1**, **2**, and **3** at varying strains from 50% to 800%. (g) Puncture force versus displacement curves of the hydrogels **1**, **2**, and **3**. (h) Adhesive shear strength versus displacement curves of the hydrogel **1** to various substrates. (i) Comparison of the adhesive shear strength of the hydrogels **1**, **2**, and **3** to various substrates.

To elucidate the unique contribution of the mechanically interlocked architecture, we prepared three hydrogels with identical monomer compositions but different cross‐linking structures. As shown in Figure 1a, hydrogel **2** employed a conventional, non‐threaded PEG_20k_‐DA cross‐linker, while hydrogel **3** utilized a pseudorotaxane cross‐linker with a non‐functionalized, classic naphthotube (**PRC‐NT**). The **NT** retains hydrogen‐bonding capacity but lacks allyl groups, thus it cannot covalently bond to the polymer network and cannot actively slide under deformation. In contrast, hydrogel **1** was fabricated using a psedurotaxane cross‐linker based on allyl‐functionalized naphthotube (**PRC‐ANT**), which enables covalent locking the macrocycles within the network. As summarized in Figure 2a,b, hydrogel **1**, featuring the covalently locked polyrotaxane network, successfully circumvented the conventional stiffness‐toughness trade‐off. At equivalent cross‐linker concentrations, it exhibited simultaneously higher tensile strength and greater elongation at break than both control hydrogels. This enhancement originates from the distinctive “pulley effect” afforded by the mobile macrocyclic junctions [30, 31, 32]. Under deformation, the **ANT** units can slide along the threaded PEG chains, facilitating efficient stress distribution and dissipation across the entire network (Figure 1b). In contrast, the static, fixed cross‐links in hydrogels **2** and **3** lead to localized stress concentration on shorter network strands, resulting in premature failure.

For practical wearable applications, long‐term durability under cyclic deformation is paramount [57]. Successive tensile loading‐unloading tests at 100% strain revealed hydrogel **1**’s exceptional resilience. It withstood 100 consecutive cycles with minimal hysteresis, a residual strain below 5%, and a stable hysteresis ratio remaining under 8% after the first cycle (Figure 2c; Figure S8). To further quantify network integrity, we calculated the input energy retention (W_total,n_/ W_total,1_) over 100 cycles. After 100 cycles, hydrogel **1** retains approximately 80% of its initial input energy, whereas hydrogels **2** and **3** retain only 58% and 41%, respectively (Figure S9). This outstanding recoverability is directly ascribed to the dynamic, reversible sliding of the **ANT** units, which dissipates energy with each cycle without causing permanent network damage [58]. Furthermore, both control hydrogels **2** and **3** showed significant stress decay and poor recovery (Figure 2d; Figures S10–S12). This dramatic difference indicates that the load‐bearing capacity of controls progressively deteriorates due to cumulative network damage, while hydrogel **1** maintains structural integrity through reversible sliding.

To further probe the energy dissipation mechanism, we compared the hysteresis behavior of the three hydrogels under different applied strains. As the strain increased from 50% to 800%, the hysteresis loops of hydrogel **1** enlarged progressively, and the calculated dissipated energy rose from 0.44 to 24.8 kJ/m^3^ (Figure 2e; Figure S13). As shown in Figure 2f and Figure S14, hydrogel **1** exhibits lower hysteresis ratios (14%–20%) across all tested strains compared to hydrogels **2** and **3** (e.g., at 50% strain: 14% for hydrogel **1** vs. 37% and 36% for controls). This strain‐dependent behavior confirms that larger deformations activate more extensive sliding motion within the polyrotaxane domains, thereby dissipating greater energy while the covalent backbone preserves overall integrity.

Microstructural evidence further supports the distinct network homogeneity. SEM imaging (Figure S15) reveals that hydrogel **1** displays a uniform, fine pore structure with smooth, well‐defined pore walls. In contrast, hydrogels **2** and **3** exhibit larger, more irregular pores with thicker, rougher walls, indicating less uniform network formation. The network's ability to resist localized damage was evaluated via puncture tests using a 1 mm diameter metal needle (Scheme S4). Hydrogel **1** displayed remarkable penetration resistance, undergoing a large, umbrella‐like deformation without rupture even at a displacement of 11 cm (Figure 2g; Video S1). In stark contrast, the control hydrogel **2** was readily punctured under identical conditions. Although hydrogel **3** also did not rupture, its puncture load was lower than that of hydrogel **1**. These results highlight the critical role of the energy‐dissipative interlocked network in blunting crack propagation [59].

Finally, the influence of bulk network properties on interfacial adhesion was investigated. While all hydrogels possess the same adhesive functional groups and can adhere to diverse substrates such as porcine skin, stainless steel, and glass (Figure 2h; Figure S16), hydrogel **1** exhibited significantly higher adhesive shear strength than the control hydrogels **2** and **3** (Figure 2i; Figure S17). This disparity underscores that the superior adhesion stems from bulk energy dissipation rather than surface chemistry. During the peeling process, the sliding polyrotaxane cross‐links in hydrogel **1** enable massive stress distribution and chain elongation, dissipating substantial energy through internal friction and network rearrangement. This mechanism effectively hinders interfacial crack propagation, thereby elevating the practical measured adhesion strength.

Collectively, the combination of control experiments, strain‐dependent hysteresis, input energy retention analysis, and microstructural evidence substantiates that the mechanically interlocked polyrotaxane network is pivotal to the superior mechanical performance of hydrogel **1**. Its performance stems from a synergistic architecture that harmonizes the energy‐dissipative dynamics of supramolecular sliding with the structural permanence of a covalent network.

### 2.4 Sensing Performance and Human Motion Detection

**FIGURE 3:**
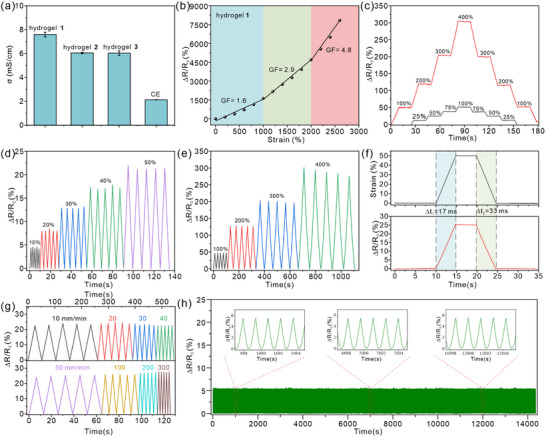
Electrical properties and mechano‐electrical response of the hydrogel **1**. (a) Conductivity of the hydrogel **1**, compared with control hydrogels (**2**, **3**) and a commercial gel electrode (CE). (b) Relative resistance changes as a function of applied strain of the hydrogel **1**; gauge factors for different strain ranges are indicated. (c) Stepwise strain‐sensing response with corresponding ∆R/R_0_ signal plateaus of the hydrogel **1**. (d) Cyclic electromechanical response under (d) small (10%–50%) and (e) large (100%–400%) tensile strains. (f) Synchronized mechanical strain and electrical resistance‐time profiles, highlighting response/recovery times. (g) Influence of tensile speed on the ΔR/R_0_ signal at 50% strain. (h) Long‐term sensing stability over 10 000 cycles at 20% strain.

The presence of mobile charge carriers (H^+^ and Na^+^ ions) endows both hydrogel **1** and control hydrogels (**2** and **3**) with substantial ionic conductivity (Figure 3a). The reversible change in conductivity under strain was visually evidenced using an LED circuit. As shown in Figure S18, stretching hydrogel **1** gradually dimmed the LED, while release restored its brightness, illustrating direct coupling between deformation and ion transport. Quantitatively, the strain‑sensing capability was assessed via the relative resistance change (∆R/R_0_), which rose monotonically with applied strain (Figure 3b; Figure S19). The gauge factor (GF), defined as GF = (∆R/R_0_)/ε, was used to quantify strain sensitivity [60]. The GF value of the hydrogel **1** was calculated to be 1.6 in the 0%–1000% strain range, increased to 2.9 between 1000% and 2000%, and further rose to 4.8 at strains up to 2500%, indicating a broad detection range with satisfactory sensitivity. Importantly, hydrogel **1** exhibited both a higher GF value compared to the control hydrogels, underscoring the superior sensing stability afforded by its interlocked network.

The sensing capability of hydrogel **1** was first evaluated through a stepwise tensile test, wherein the strain was incrementally increased from 0% to 100% and from 100% to 400%, respectively, and then stepwise decreased. As shown in Figure 3c, the relative resistance change (∆R/R_0_) exhibited corresponding stepwise variations with excellent stability at each plateau, demonstrating its capacity to steadily monitor multilevel strains in real‐time. Notably, the loading and unloading strain sensing curves were nearly symmetric without resistance hysteresis, indicating minimal electrical hysteresis and excellent material resilience. This directly reflects the reversible disruption and reformation of ionic conduction pathways, enabled by the polymer network's excellent elasticity.

Subsequently, the performance of hydrogel **1** under dynamic conditions was evaluated through continuous cyclic loading‐unloading tests across a wide range of strains (10%–400%). As displayed in Figure 3d,e, hydrogel **1** is able to accurately and successively monitor the response signals of both small (10%–50%) and larger (100%–400%) deformations, with highly consistent electrical signals in each cycle, confirming its sensitivity and reliability. The electromechanical response speed was quantified by measuring the time difference between the mechanical action (recorded by a universal testing machine) and the corresponding electrical signal change (recorded by a source meter) [61]. At 50% strain and a speed of 100 mm·min^−1^, the response times for stretching and recovery were 17 and 33 ms, respectively (the absolute times are ≈5 s), which are notably faster than the typical response of human skin (Figure 3f) [53] Furthermore, the electrical output signals of the hydrogel **1** remained stable across a wide range of tensile speeds (10–300 mm/min), demonstrating a speed‐independent characteristic essential for practical applications involving variable motion rates (Figure 3g).

More importantly, hydrogel **1** displayed outstanding long‐term sensing durability. To evaluate its long‐term stability, we extended the cyclic test to 10 000 cycles at 20%, 50% and 100% strain, respectively (Figure 3h; Figure S20). Remarkably, the electrical response of hydrogel **1** remained exceptionally stable across all tested strain amplitudes over the entire 10 000 cycles, with no observable drift or degradation in the peak ∆R/R_0_ values. The signal waveforms at each strain amplitude were highly consistent throughout the test, demonstrating excellent long‐term cyclic stability regardless of deformation magnitude. In contrast, the signals from control hydrogels **2** and **3** degraded progressively within 1000 cycles (Figure S21). This outstanding stability directly validates the structural integrity and anti‐fatigue character imparted by the synergistic, mechanically interlocked architecture, which prevents the accumulation of irreversible damage in the conductive network.

**FIGURE 4:**
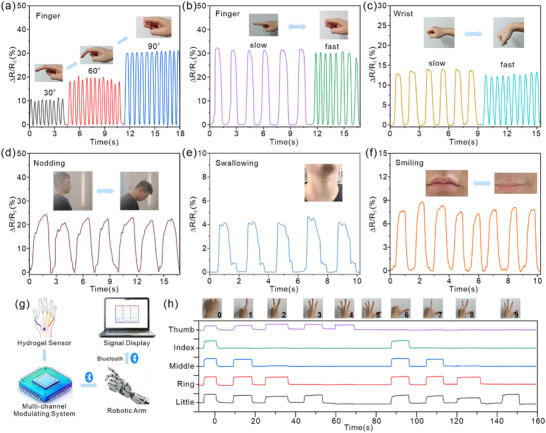
Human activity monitoring and human–machine interaction enabled by hydrogel **1**. Real‐time relative resistance (ΔR/R_0_) responses of the hydrogel sensor during: (a) finger bending at various angles, (b) finger bending at different speeds, (c) wrist bending at different speeds, (d) head nodding, (e) swallowing, and (f) smiling. (g) Schematic of a wireless human–machine interaction system integrating a hydrogel sensor array for gesture‐based control. (h) Distinct ΔR/R_0_ signal patterns corresponding to ten finger gestures (digits 0–9), used for gesture recognition.

Given its skin‐like mechanical properties, robust adhesion, excellent fatigue resistance, and reliable electromechanical response, hydrogel **1** demonstrates the most balanced and comprehensive performance profile among the previously reported slide‐ring hydrogel sensors (Table S3 and Figure S22). This unique combination of attritubes positions the hydrogel **1** as a promising candidate for wearable sensors to monitor human activities and recognize gestures. A sensor fabricated from hydrogel **1** was conformally attached to various joints to detect different human motions. The associated strain/pressure changes induced corresponding, measurable variations in the sensor's resistance. As demonstrated in Figure 4a, bending a finger from 0° to 90° induced a rapid, measurable increase in sensor resistance due to hydrogel film stretching. During repeated cycles of finger bending and straightening, the resistance changes remained highly consistent, confirming precise and stable tracking of finger motions. The sensor also responded accurately to changes in bending frequency, generating stable signals at different movement speeds (Figure 4b). Beyond finger motions, the hydrogel sensor successfully monitored larger and repetitive joint movements, including flexion/extension of the wrist, elbow, and knee, as well as head nodding (Figure 4c,d; Figure S23), showcasing its broad potential for monitoring comprehensive physical exercises.

The high strain sensitivity of the sensor enables it to effectively capture subtle physiological activities, such as breathing, swallowing, smiling, and speech. As shown in Figure 4e and Figure S24, when attached to a volunteer's throat, the sensor produced repeatable resistance signals that accurately mirrored the rhythmic patterns of regular swallowing and breathing. With the sensor placed at the corner of the mouth, the minute muscle movements induced by repetitive smiling were clearly differentiated with excellent reproducibility (Figure 4f). Furthermore, when the volunteer repeatedly spoke the specific words (“How are you” or “I'm fine”), the resultant relative resistance variation curves exhibited similar (Figure S25), characteristic waveforms, demonstrating the sensor's capacity to discern articulated speech patterns.

To further explore its potential for human–machine interaction (HMI), we developed a wireless wearable sensing system based on the hydrogel sensor. An array of five hydrogel sensors was integrated onto each finger to capture real‐time motion dynamics of the entire hand (Figure 4g). A customized signal processing module acquires the multi‐channel data and transmits it via Bluetooth to control a robotic arm, enabling it to accurately mimic human hand motions such as object grasping (Figure S26 and Video S2). Furthermore, the relative electrical signal changes from each finger sensor can be wirelessly monitored and display real time. For instance, by measuring resistance variations across the five independent channels, distinct electrical signals corresponding to common hand gestures were successfully collected (Figure 4h; Figure S27 and Video S3). These signals can be further processed for gesture interpretation. These results collectively demonstrate the system's capability for high‐precision, multi‐channel signal acquisition and its function as a wireless control interface for HMI applications.

### 2.5 Epidermal Electrophysiological Signals Monitoring

**FIGURE 5:**
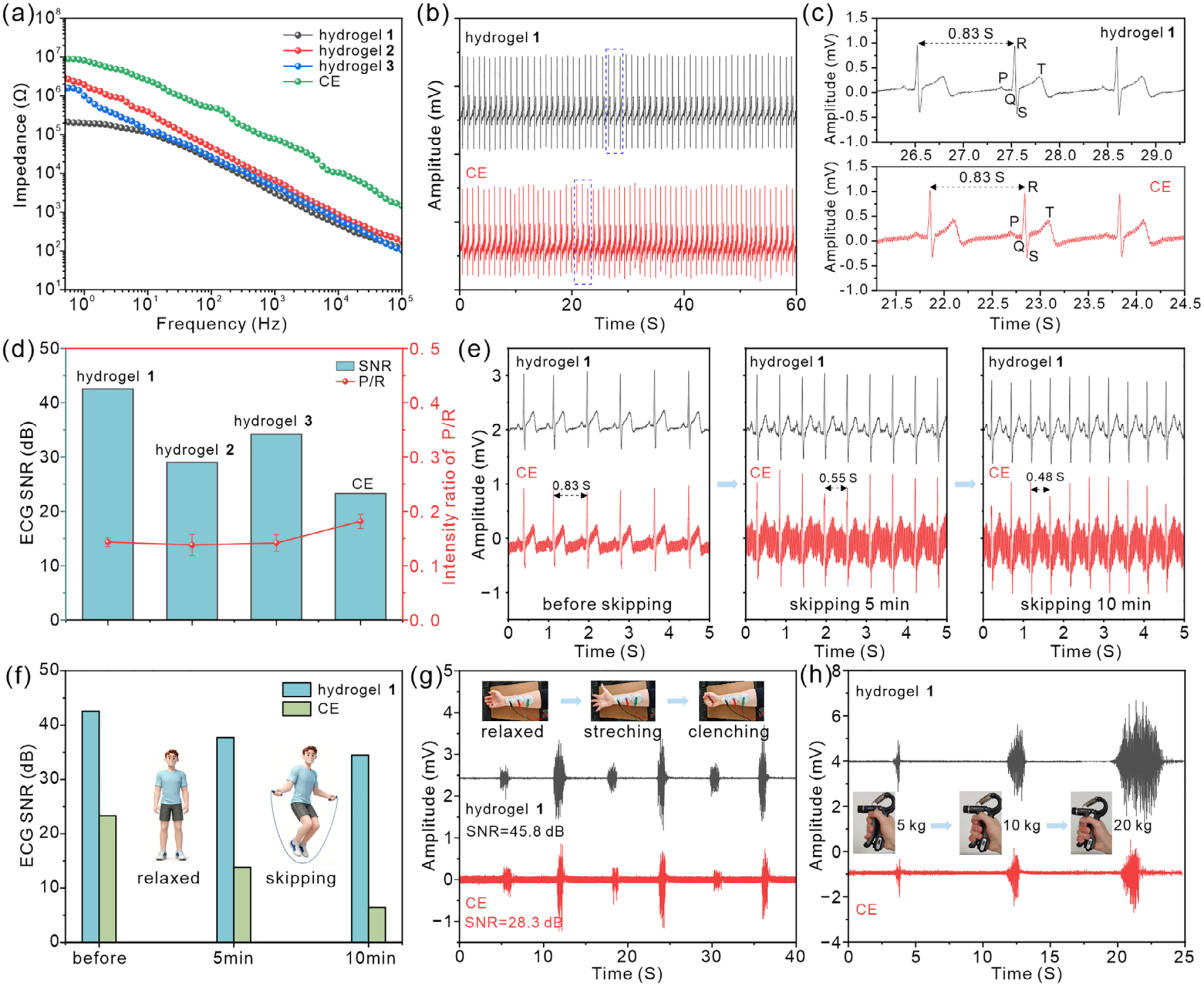
Epidermal electrophysiological signal monitoring. (a) Bode plots of electrode‐skin interface impedance for hydrogel **1**, control hydrogels (**2**, **3**), and a commercial gel electrode (CE). (b) Representative electrocardiogram (ECG) waveforms recorded by hydrogel **1** and CE. (c) Enlarged view of characteristic P‑QRS‑T complexes from (b). (d) Comparison of signal‐to‐noise ratio (SNR) and P‑wave to R‑wave amplitude ratio (P/R) among all electrodes. (e) ECG signals and (f) corresponding SNR values acquired by hydrogel **1** and CE under resting conditions and after intense skipping exercise. (g) Electromyogram (EMG) signals during repeated fist clenching, recorded by hydrogel **1** and CE. (h) EMG responses under different grip forces (5, 10, and 20 kg) measured with hydrogel **1** and CE.

Electrophysiological signals, such as the electrocardiogram (ECG) and electromyogram (EMG), carry vital information for assessing physiological states and diagnosing muscular or cardiovascular disorders [62, 63]. High‐fidelity acquisition of these signals is therefore paramount for both clinical diagnostics and the development of reliable human‑machine interfaces. We first evaluated the fundamental interfacial property of hydrogel electrodes by measuring their skin‐electrode impedance. As shown in Figure 5a, the electrode fabricated from hydrogel **1** exhibited significantly lower impedance across a broad frequency range (1–10^5^ Hz) compared to a standard commercial wet gel electrode. This superior performance originates from the synergistic combination of high bulk ionic conductivity and a tissue‑matching Young's modulus, which promotes conformal contact and maximizes the effective conductive area [64]. Notably, the electrode derived from hydrogel **1** demonstrated the lowest impedance among all tested hydrogels, a direct benefit of its optimized composition and stable network structure, thereby establishing an optimal foundation for precise biopotential acquisition.

The practical efficacy was first demonstrated through ECG monitoring. Representative ECG waveforms acquired by the hydrogel electrodes and the commercial wet gel electrode are displayed in Figure 5b,c and Figure S28. The signals captured by hydrogel **1** clearly delineate all diagnostically critical waveforms: the P wave (atrial depolarization), the QRS complex (ventricular depolarization), and the T wave (ventricular repolarization) [65]. The measured R‐R interval of approximately 0.83 s corresponds to a heart rate of 72 bpm, within the normal resting range. While the sensitivity, quantified by the P‐peak to R‐peak voltage ratio (P/R), was comparable to that of the commercial gel (Figure 5d), the signal quality differed markedly. The hydrogel electrode achieved a significantly higher signal‐to‐noise ratio (SNR) of 42.5 dB, compared to 23.3 dB for the commercial gel (Figure 5d). This enhancement is attributed to the hydrogel's superior interfacial contact and inherent noise suppression.

Crucially, the hydrogel electrode maintained exceptional signal stability under dynamic conditions. As depicted in Figure 5e,f, after the volunteer performed 10 min of skipping exercise (increasing heart rate from 72 to 125 bpm), the electrode from hydrogel **1** continued to output high‐quality ECG waveforms with an SNR above 34.5 dB. In stark contrast, the SNR of the commercial gel electrode plummeted from 23.3 dB to only 6.4 dB under the same motion‐intensive conditions. This dramatic disparity underscores that the skin‐like softness and robust, fatigue‐resistant adhesion of hydrogel **1** are indispensable for obtaining reliable biosignals during physical activity, a scenario where conventional rigid or poorly adhering electrodes typically fail.

The capability of the hydrogel electrode was further extended to EMG signal acquisition. Clear and robust EMG signals were instantaneously recorded upon fist clenching (brachioradialis contraction) and vanished upon relaxation (Figure 5g). During repetitive fist‐making cycles, the hydrogel electrode captured high‐fidelity signals with a superior SNR of 45.8 dB, compared to 28.3 dB for the commercial electrode. Furthermore, the system demonstrated quantitative monitoring capability: when the volunteer exerted graded grip forces (5, 10, 20 kg) on a dynamometer, the amplitude of the recorded EMG signal increased correspondingly with the applied force (Figure 5h; Figure S29), highlighting its potential for assessing muscle exertion levels.

To assess the practical applicability of the electrode from hydrogel **1** for prolonged wear, we systematically evaluated its long‐term stability over 24 h, including water retention, conductivity maintenance, interfacial impedance variation, and ECG signal quality. As shown in Figure S30, hydrogel **1** exhibits excellent water retention, maintaining 87.5% of its initial weight after 24 h, while the commercial electrode retains only 48.5%. Concomitantly, the ionic conductivity of hydrogel **1** decreases slightly from 7.46 to 6.64 mS/cm over 24 h, whereas the conductivity of the commercial electrode plummets from 2.13 to 0.19 mS/cm. These results demonstrate the superior hydration stability of hydrogel **1**, attributed to its uniform network structure and the hydrophilic nature of sodium polyacrylate that effectively traps water molecules within the matrix. Furthermore, the skin‐electrode impedance of hydrogel **1** remains remarkably stable with negligible change over 24 h (Figure S31), while the commercial electrode shows a slight upward trend that becomes apparent after 8 h. Subsequently, we continuously recorded ECG signals over 24 h using both electrodes. As shown in Figure S32, hydrogel **1** maintains a high SNR above 28 dB even after 24 h of continuous wear, with all diagnostically critical features (P‐wave, QRS complex, T‐wave) remaining clearly distinguishable throughout the test period. In contrast, the commercial electrode's SNR drops below 9.8 dB by 24 h, and the ECG waveform becomes severely distorted with loss of identifiable features after 12 h.

Collectively, these results demonstrate that the hydrogel electrode, enabled by its intrinsically low interfacial impedance, skin‐conformal mechanics, motion‐stable adhesion, and excellent long‐term stability, provides superior signal quality, exceptional stability during physical activity, and quantitative accuracy in monitoring both cardiac and muscular electrical activity. This integrated performance profile underscores its strong potential for use in clinical‐grade wearable electrophysiology and advanced, dynamic human–machine interfaces.

## 3 Conclusion

In summary, we have developed a novel conductive hydrogel through the strategic construction of a mechanically interlocked polyrotaxane network, which effectively reconciles the conflicting requirements of mechanical robustness and electrical reliability for wearable bioelectronics. By covalently locking dynamically sliding naphthotube macrocycles within a cross‐linked polymer matrix, the hydrogel achieves an exceptional combination of skin‐like softness, ultrahigh stretchability, exceptional fatigue resistance, strong adhesion, and high ionic conductivity. This unique architecture is proven pivotal for enabling reliable dual functionality. It functions simultaneously as a durable strain sensor with broad‐range and stable sensing capability (10 000 cycles), and as a superior epidermal electrode for acquiring high‐fidelity, motion‐robust electrophysiological signals with excellent long‐term stability over 24 h. These integrated functionalities underscore its potential as a versatile platform for advanced wearable applications. Ultimately, this work establishes a new design paradigm based on mechanically interlocked supramolecular networks, offering a versatile pathway toward next‐generation, high‐performance wearable devices for healthcare monitoring and interactive technologies.

Despite these promising results, several limitations warrant further investigation. For instance, while the hydrogel demonstrates good water retention, further improvement in long‐term anti‐dehydration would enhance its operational lifespan for extended wear applications. Additionally, systematic in vivo biocompatibility and skin irritation studies are necessary before clinical translation. Furthermore, the current synthetic procedure is relatively complex and currently limited to laboratory‐scale preparation, developing simplified and scalable preparation strategies would facilitate practical application. Addressing these challenges will further advance polyrotaxane‐based mechanically interlocked hydrogels toward real‐world wearable bioelectronic applications.

## Conflicts of Interest

The authors declare no conflicts of interest.

## Supporting information




**Supporting File 1**: advs75205‐sup‐0001‐SuppMat.docx.

**Supporting File 2**: advs75205‐sup‐0002‐VideoS1.mp4.

**Supporting File 3**: advs75205‐sup‐0003‐VideoS2.mp4.

**Supporting File 4**: advs75205‐sup‐0004‐VideoS3.mp4.

## Data Availability

The data that supports the findings of this study are available in the supplementary material of this article.
