# Supplementary material for: A Stretchable, Mechanically‐Interlocked Polyrotaxane Hydrogel for Wearable Motion and Electrophysiological Monitoring
Source: Adv Sci (Weinh). 2026 Apr 10:e75205. Online ahead of print. doi: 10.1002/advs.75205 (PMC13334668; doi:10.1002/advs.75205)
Supplement: Supplementary file 1 — Supporting File 1: advs75205‐sup‐0001‐SuppMat.docx. [file ADVS-9999-e75205-s002.docx]

**Table of contents**

1. **General**
2. **Preparation of allyl-functionalized naphthotube ANT**
3. **2D NMR of the Configurational Isomers of S7**
4. **X-Ray Crystallography of *syn*-S7 and *anti*-S7**
5. **Methods**
6. **Supporting Figures**
7. **References**
8. General

All the reagents involved in this research were commercially available and used without further purification unless otherwise noted. Solvents were either employed as purchased or dried prior to use by standard laboratory procedures. Thin-layer chromatography (TLC) was carried out on 0.25 mm silica gel plates (Anhui Liangchen, GF-254). Column chromatography was performed on silica gel 60 (Anhui Liangchen 40-63 nm, 200-300 mesh). ^1^H, and ^13^C NMR spectra were recorded on a Bruker Avance-400, 500 or 600 NMR spectrometer at 25 ^o^C. All chemical shifts are reported in ppm with residual solvents or TMS (tetramethylsilane) as the internal standards. The following abbreviations were used to report the multiplicity for NMR resonances: s=singlet; d=doublet; dd=doublet of doublet; t=triplet, m=multiplet. Electrospray-ionization high resolution mass spectrometry (ESI-HRMS) experiments were conducted on an applied Q EXACTIVE mass spectrometry system. The tensile, adhesive and puncture experiments were record on an electrical universal test machine (UTM2102, Shenzhen Suns Technology Stock Co., Ltd.). The conductivity was measured by system (RTS-8, Guangzhou Four-Probe Technology Co., Ltd.) in conjunction with a digital source meter (2450, Keithley). The probe spacing is 1.59 mm. The electrical experiments was record on a digital source meter (Keithley, 2450). The epidermal electrophysiological signals were collected on a multi-channel biosignal acquisition and processing system (RM6240XC, Chengdu Instrument Factory). Scanning electron microscope (SEM) images were obtained from the field emission SEM (Regulus 8100).

**2. Preparation of allyl-functionalized naphthotube MANT**


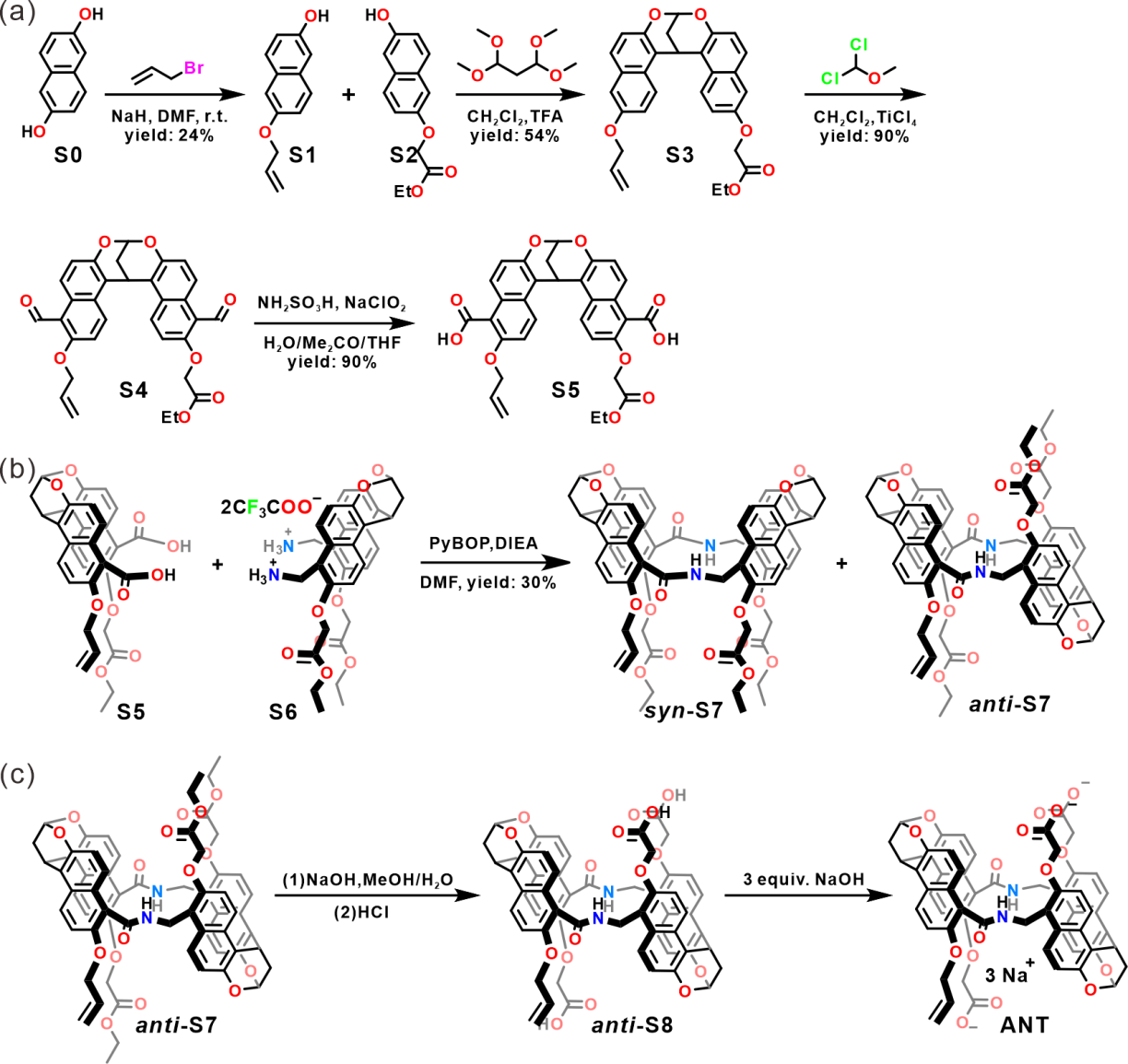


***Scheme S1*.** Synthesis of allyl-functionalized naphthotube **ANT**.

The synthesis scheme of **ANT** follow the procedures described above, in which, **S2** and **S6** have been reported previously.^[1],[2]^ Detailed synthetic procedures for **ANT** are provided below. Previous work from our group demonstrated that the *anti*-configured naphthotube binds PEG more stronger than its *syn*-configured counterpart.^[3]^ Therefore, the *anti*-configured allyl-naphthotubes, ***anti*-S7** was selected to prepare the water-soluble macrocyclic host **ANT** for preparing hydrogels.

**Synthesis of S1**

In a 2 L flask under an argon atmosphere, 2,6-naphthalenediol (100 g, 624 mmol) was dissolved in N,N-dimethylformamide (DMF, 1 L). Sodium hydride (60% dispersion in mineral oil, 23.73 g, 593.1 mmol) was added slowly to the cooled (ice bath) solution. After stirring at 0 ^o^C for 1 h, a solution of allyl bromide (68 g, 48.6 mL, 562 mmol) in DMF (200 mL) was added dropwise. The reaction mixture was then warmed to room temperature and stirred for an additional 12 h. The mixture was poured into ice water (1 L) and acidified to approximately pH 7 using aqueous HCl. The resulting precipitate was collected by filtration and purified by silica gel column chromatography (eluent: PE/CH_2_Cl_2_) to afford compound **S1** as a white solid (29.4 g, 24% yield).

**S1**, white solid, m.p. > 300 ^o^C. ^1^H NMR (500 MHz, DMSO-*d_6_*, 298K) δ [ppm] 9.49 (s, 1H), 7.61 (dd, J = 16.6, 8.9 Hz, 2H), 7.21 (d, J = 2.6 Hz, 1H), 7.12–6.99 (m, 3H), 6.09 (ddt, J = 17.3, 10.5, 5.2 Hz, 1H), 5.43 (dq, J = 17.3, 1.8 Hz, 1H), 5.27 (dq, J = 10.5, 1.5 Hz, 1H), 4.61 (dt, J = 5.3, 1.6 Hz, 2H). ^13^C NMR (126 MHz, DMSO-*d*_6_, 298K) δ [ppm] = 154.33, 154.07, 134.29, 130.33, 128.92, 128.54, 127.96, 119.33, 119.27, 117.83, 109.34, 107.78, 68.64. ESI-HRMS: m/z calcd for [M-H]^-^ C_13_H_11_O_2_^-^, 199.0765; found 199.8043(error = -2.8 ppm).


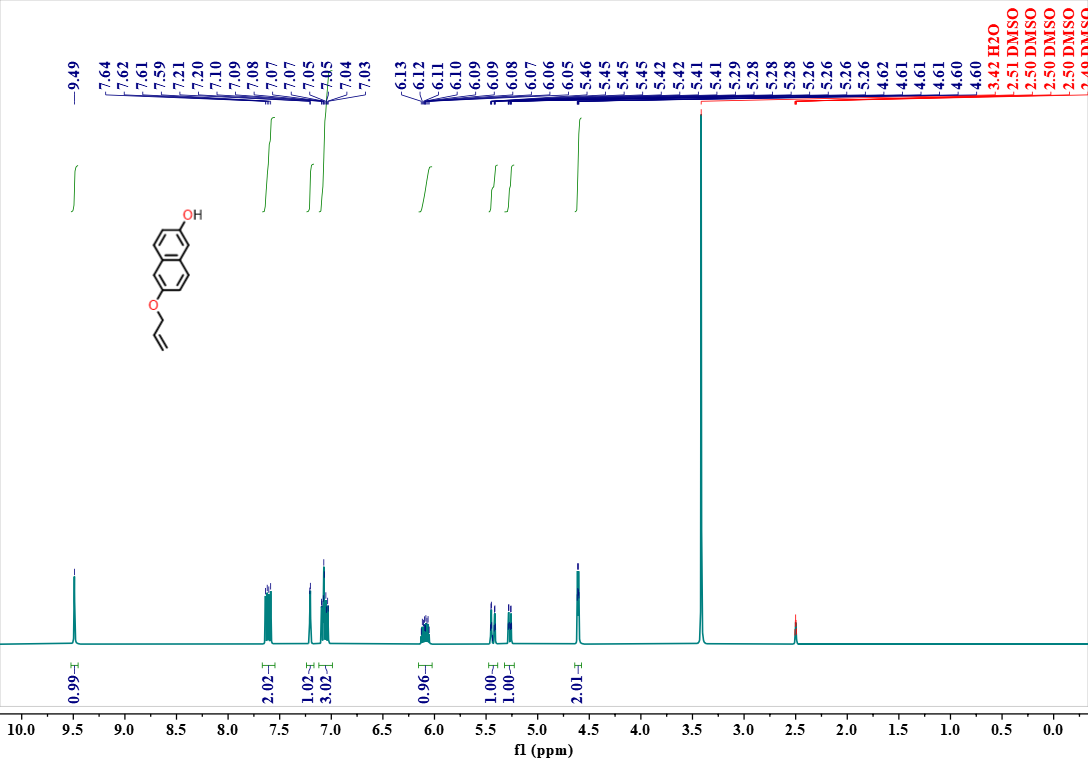


^1^H NMR spectrum (500 MHz, DMSO-*d*_6_, 298 K) of **S1**


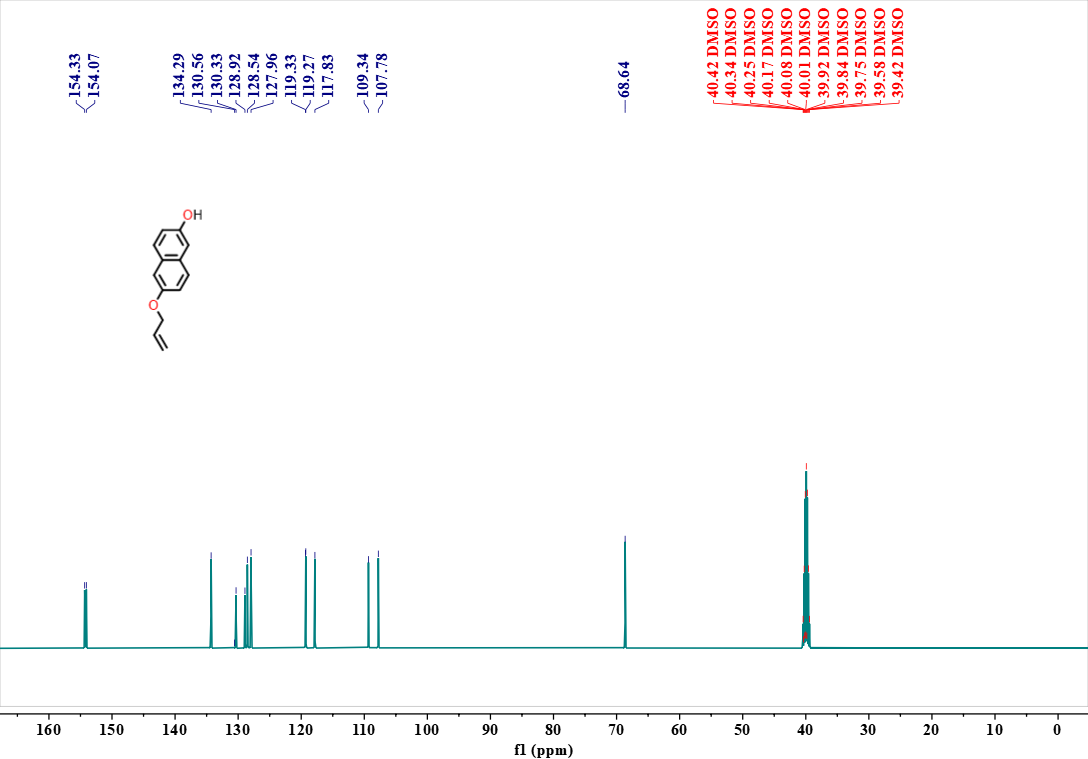


^13^C NMR spectrum (126 MHz, DMSO-*d*_6_, 298 K) of S**1**


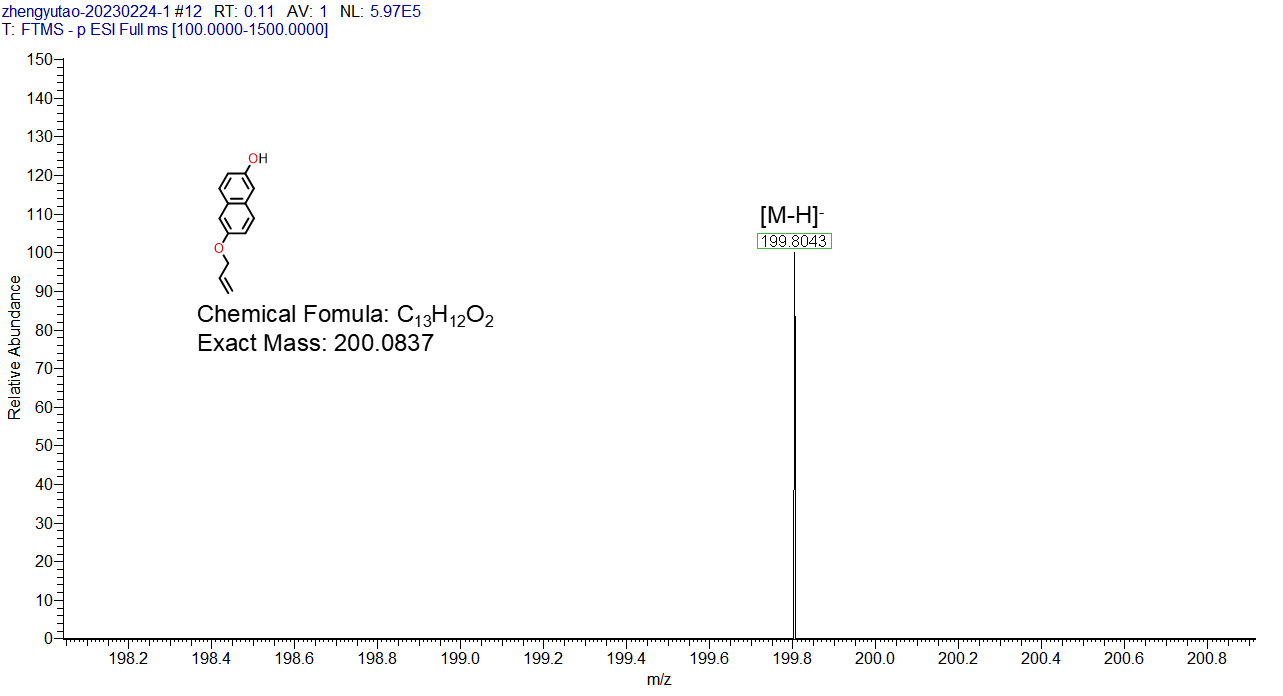


ESI-HRMS mass spectrum of **S1**

**Synthesis of S3**

A solution of **S1** (15.4 g, 77 mmol), **S2** (18.9 g, 77 mmol), and trifluoroacetic acid (TFA, 78 mL) in dichloromethane (DCM, 300 mL) was added dropwise over 15 minutes to a solution of 1,1,3,3-tetramethoxypropane (12.6 g, 77 mmol) in DCM (100 mL) under an argon atmosphere. After complete addition, the reaction mixture was stirred at room temperature for 12 h. The mixture was then poured into ice water and carefully neutralized with saturated aqueous sodium bicarbonate solution (NaHCO_3_). The resulting suspension was extracted with DCM (3×100 mL). The combined organic extracts were concentrated under reduced pressure, and the crude residue was purified by silica gel column chromatography (eluent: PE/CH_2_Cl_2_) to afford: **S3** as a white solid (10.2 g, 27% yield).

**S3**, white solid, m.p. > 300 ^o^C; ^1^H NMR (600 MHz, DMSO-*d*_6_, 298K) δ [ppm] 8.65 (t, J = 10.7 Hz, 2H), 7.56 (dd, J = 8.9, 3.0 Hz, 2H), 7.29–7.18 (m, 4H), 7.10 (dd, J = 8.9, 5.3 Hz, 2H), 6.34 (s, 1H), 6.07 (ddt, J = 16.1, 10.5, 5.2 Hz, 1H), 5.53 (s, 1H), 5.41 (dd, J = 17.1, 1.9 Hz, 1H), 5.25 (d, J = 10.5 Hz, 1H), 4.82 (s, 2H), 4.66–4.56 (m, 2H), 4.15 (q, J = 7.1 Hz, 2H), 2.39 (s, 2H), 1.19 (t, J = 7.1 Hz, 3H). ^13^C NMR (151 MHz, DMSO-*d*_6_, 298K) δ [ppm] 169.14, 154.73, 154.20, 148.91, 148.69, 134.14, 130.78, 130.57, 127.53, 127.47, 126.77, 126.47, 125.54, 125.42, 119.74, 119.68, 119.06, 118.93, 118.85, 118.48, 117.91, 108.98, 108.88, 91.40, 68.68, 65.27, 61.09, 40.41, 40.28, 26.60, 22.12, 14.50. ESI-HRMS: m/z calcd for [M+H]^+^ C_30_H_27_O_6_^+^, 483.1802; found 483.1804 (error = 0.4 ppm).


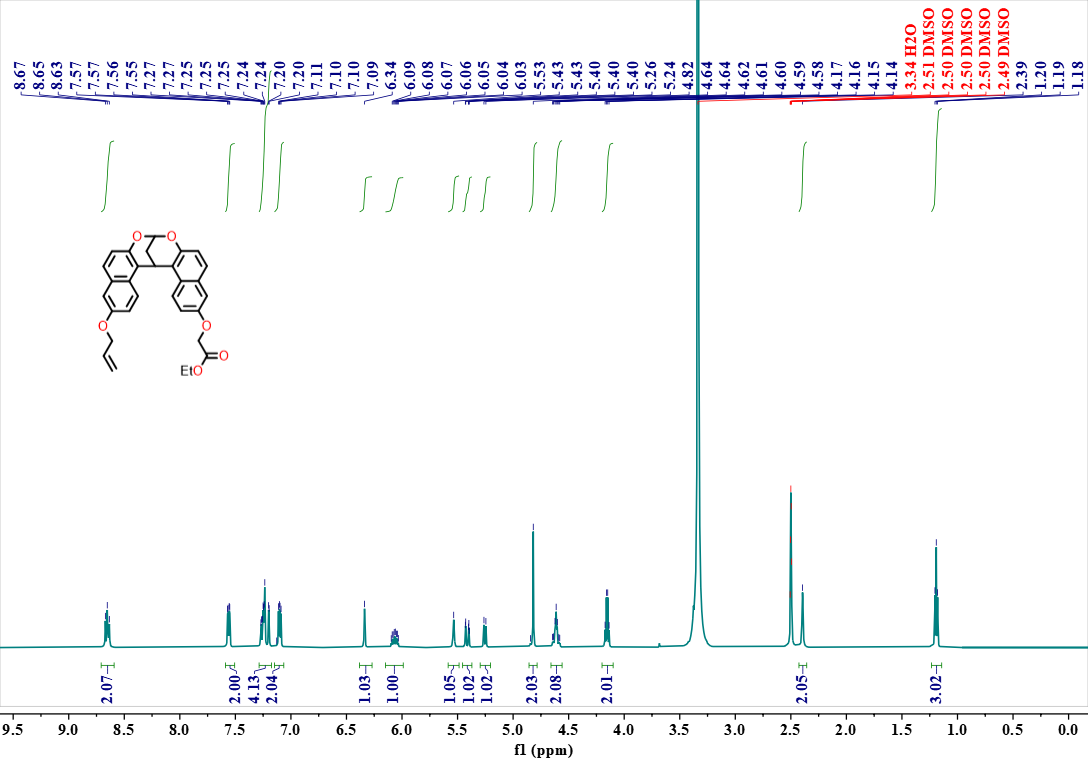


^1^H NMR spectrum (600 MHz, DMSO-*d*_6_, 298 K) of **S3**


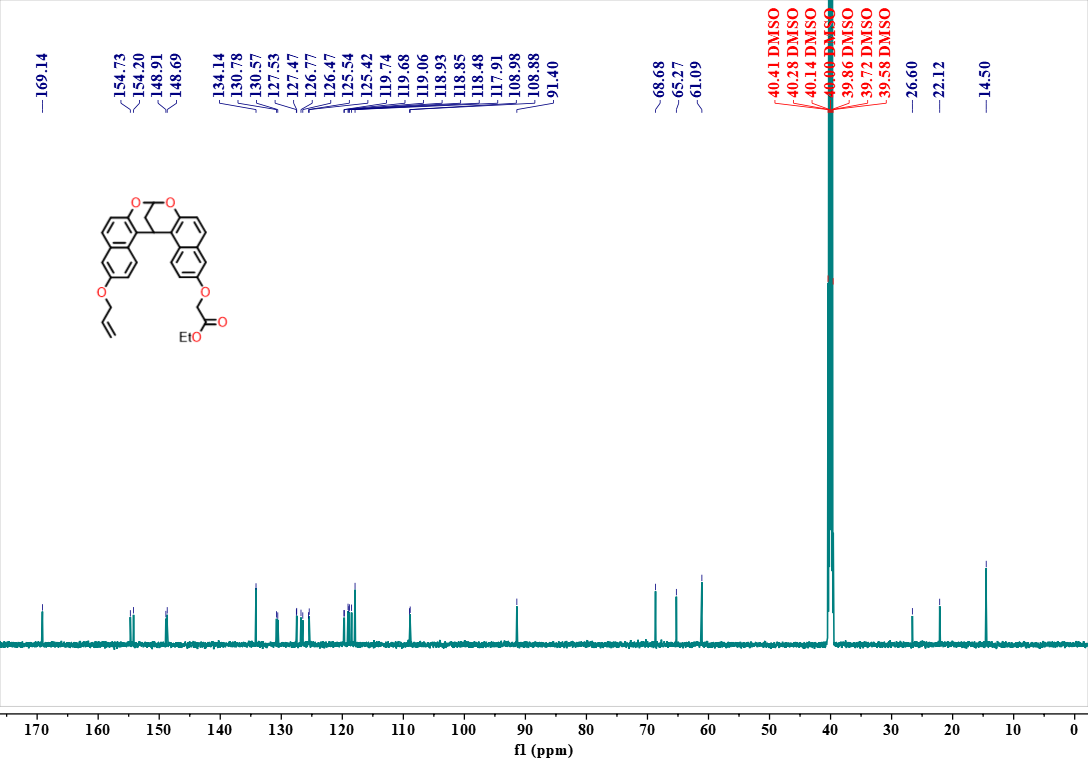


^13^C NMR spectrum (151 MHz, DMSO-*d*_6_, 298 K) of **S3**


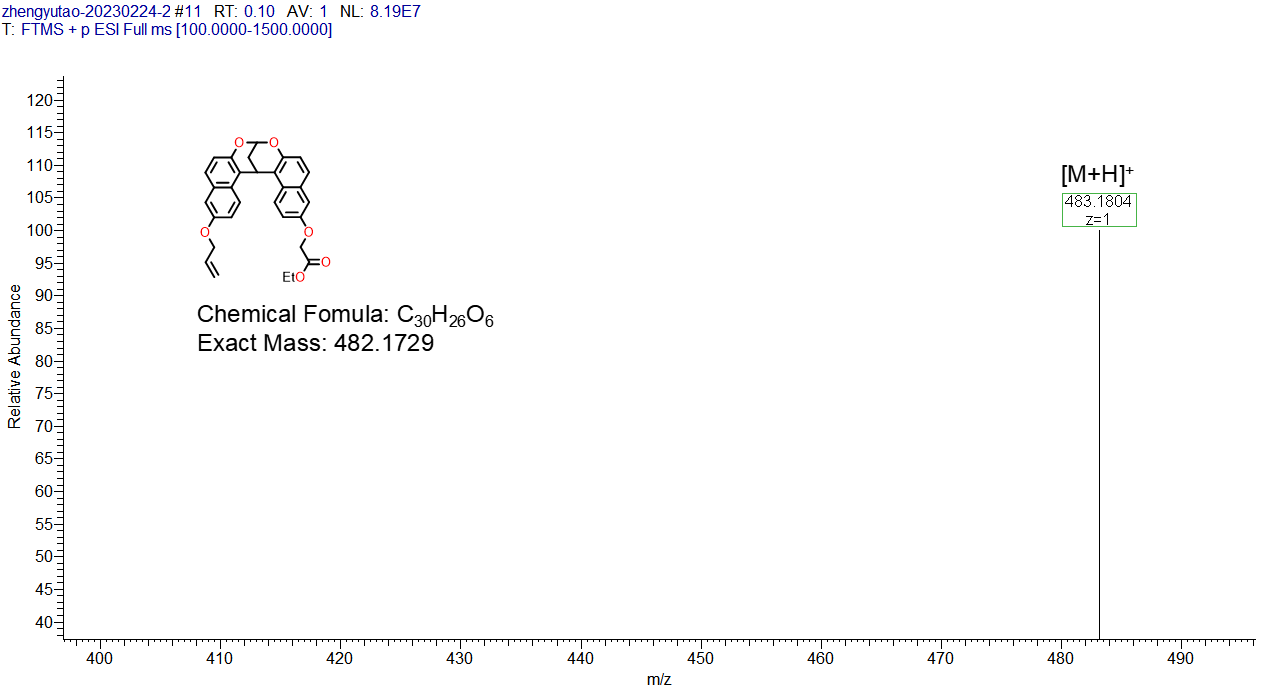


ESI-HRMS mass spectrum of **S3**

**Synthesis of S4**

1,1-Dichlorodimethyl ether (7.6 mL, 84 mmol) was added slowly to a solution of **S3** (10.2 g, 21 mmol) in dichloromethane (DCM, 250 mL) at 0 ^o^C under an argon atmosphere. After stirring for 10 minutes, a solution of titanium tetrachloride (TiCl_4_, 9.3 mL, 84 mmol) in DCM (70 mL) was added dropwise to the reaction mixture over 1 h. Upon complete addition, the mixture was stirred at room temperature for 12 h. The reaction was quenched by careful addition of saturated aqueous sodium bicarbonate solution (NaHCO_3_, 500 mL). The mixture was then extracted with DCM (3×100 mL). The combined organic layers were dried over anhydrous sodium sulfate (Na_2_SO_4_), filtered, and concentrated under reduced pressure. The resulting crude product was recrystallized from acetone to afford **S4** as a yellow solid (10.2 g, 90% yield).

**S4**, yellow solid, m.p. > 300 ^o^C; ^1^H NMR (600 MHz, DMSO-*d*_6_, 298K) δ [ppm] 10.76 (d, J = 27.0 Hz, 2H), 9.06 (dd, J = 9.6, 7.5 Hz, 2H), 8.90 (dd, J = 9.4, 4.3 Hz, 2H), 7.56 (dd, J = 22.5, 9.6 Hz, 2H), 7.30 (dd, J = 12.0, 9.3 Hz, 2H), 6.40 (q, J = 1.8 Hz, 1H), 6.13 (ddt, J = 17.3, 10.5, 5.2 Hz, 1H), 5.72 (d, J = 3.5 Hz, 1H), 5.46 (dq, J = 17.3, 1.7 Hz, 1H), 5.32 (dt, J = 10.6, 1.5 Hz, 1H), 5.18–5.07 (m, 2H), 4.94–4.82 (m, 2H), 4.18 (q, J = 7.1 Hz, 2H), 2.46 (t, J = 2.7 Hz, 2H), 1.19 (t, J = 7.1 Hz, 3H). ^13^C NMR (151 MHz, DMSO-*d*_6_, 298K) δ [ppm] 191.57, 191.30, 168.47, 161.12, 160.59, 149.00, 148.86, 133.10, 132.50, 132.38, 126.71, 126.57, 126.35, 125.91, 124.23, 124.14, 121.53, 121.39, 119.75, 119.72, 118.07, 117.00, 116.64, 115.05, 114.82, 90.92, 69.92, 66.13, 60.92, 25.82, 21.50, 13.99. ESI-HRMS: m/z calcd for [M+H]^+^ C_32_H_27_O_8_^+^, 539.1700; found 539.1703 (error = 0.6 ppm).


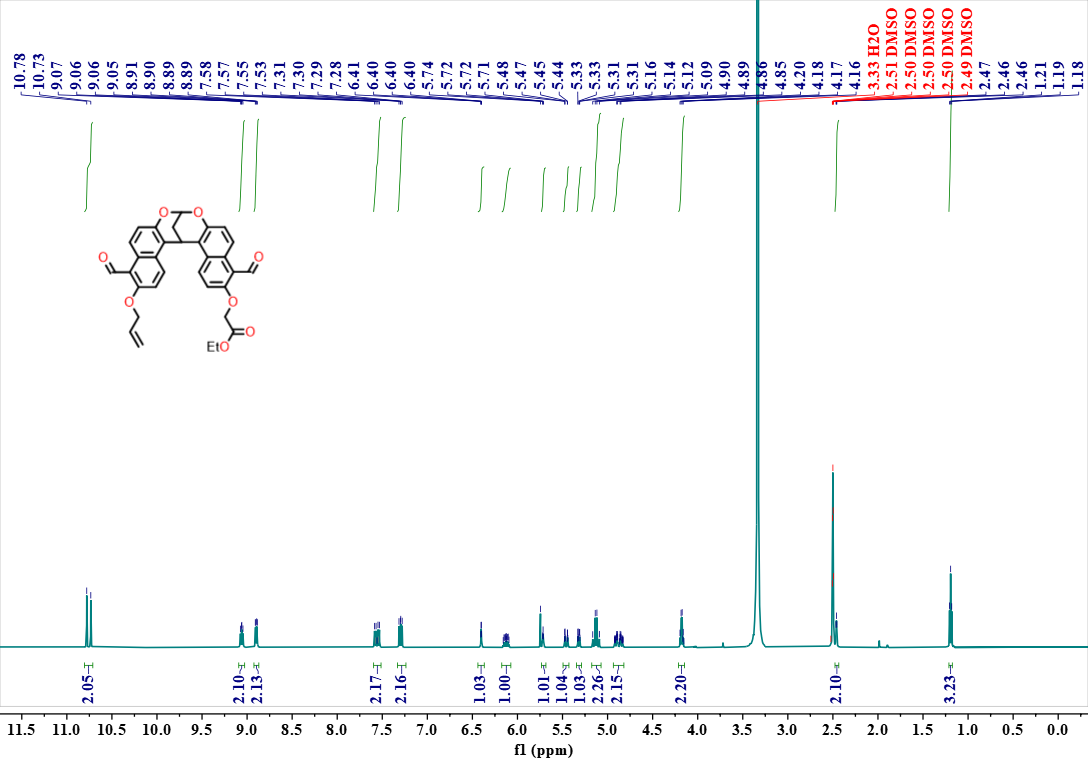


^1^H NMR spectrum (600 MHz, DMSO-*d*_6_, 298 K) of **S4**


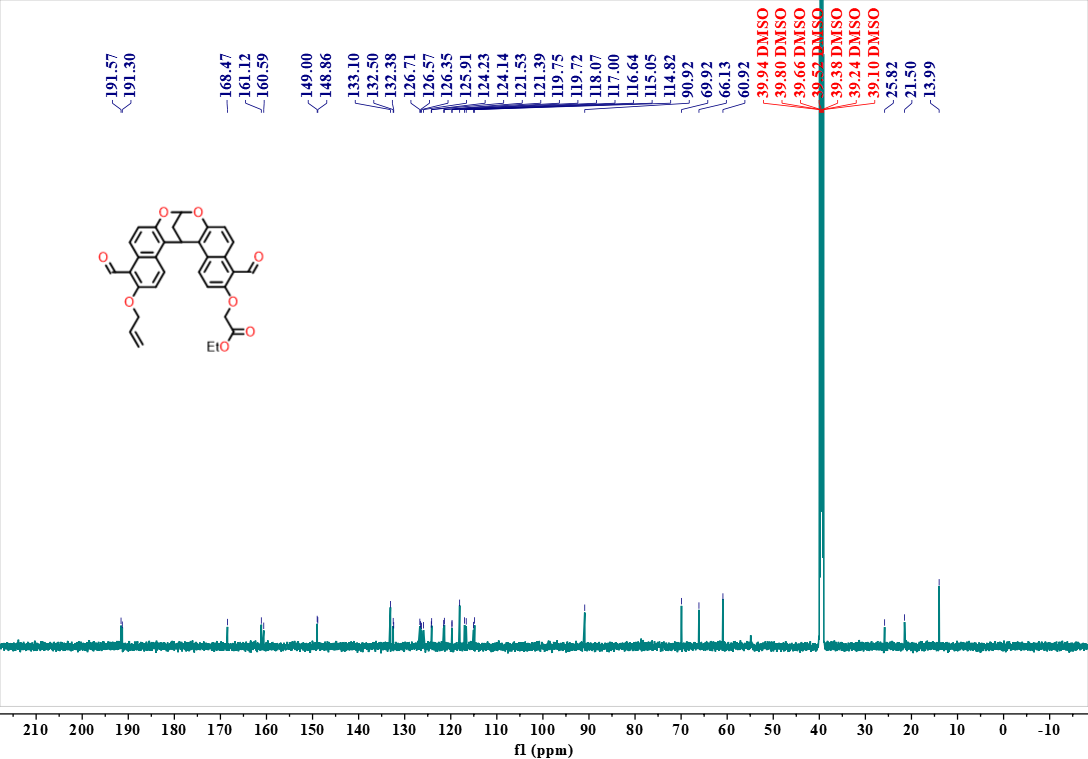


^13^C NMR spectrum (151 MHz, DMSO-*d*_6_, 298 K) of **S4**


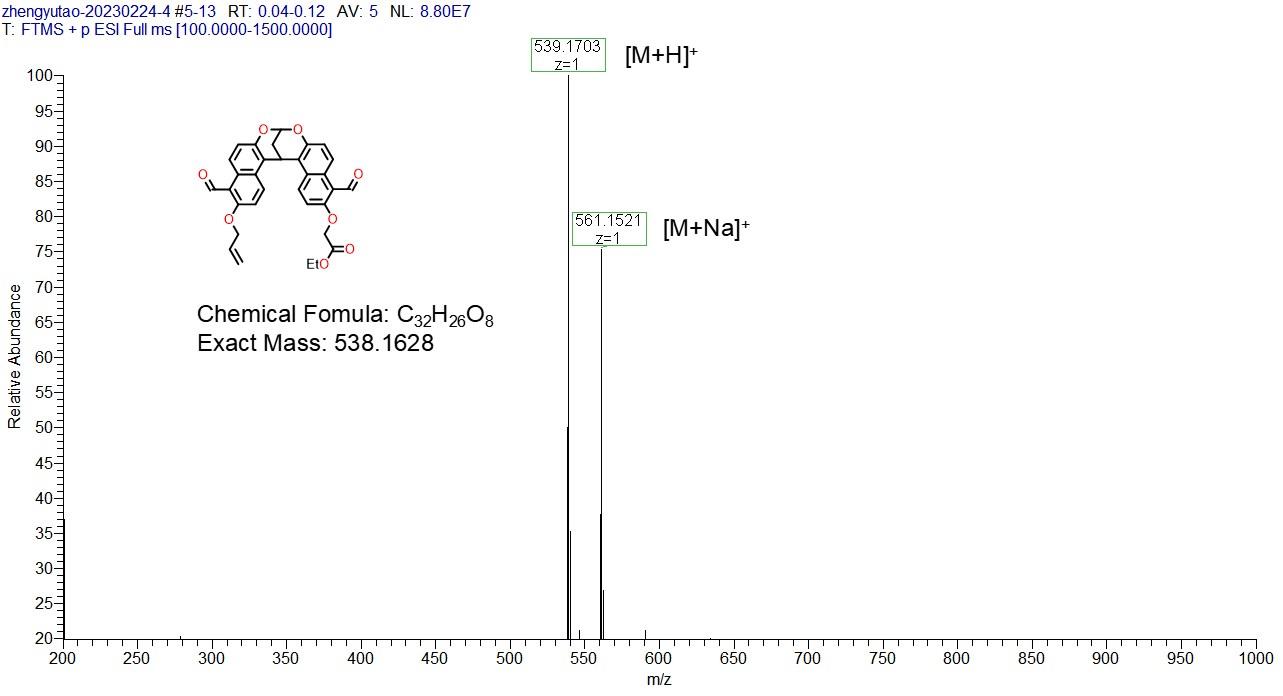
ESI-HRMS mass spectrum of **S4**

**Synthesis of S5**

Sodium chlorite (NaClO_2_, 11.2 g, 123 mmol) was added portionwise to a mixture of compound **S4** (8.2 g, 15.2 mmol), sulfamic acid (NH_2_SO_3_H, 11.1 g, 114 mmol), water (220 mL), tetrahydrofuran (THF, 220 mL), and acetone (332 mL). The resulting mixture was stirred at room temperature for 12 hours and then concentrated under reduced pressure to remove volatile solvents. The pH of the residue was adjusted to approximately 5–6 using aqueous HCl solution (1.0 N). The precipitated solid was collected by filtration, washed thoroughly with deionized water, and dried to afford **S5** as a pale-yellow solid (7.8 g, 90% yield).

**S5**, pale-yellow solid, m.p. > 300 ^o^C; ^1^H NMR (600 MHz, DMSO-*d*_6_, 298K) δ [ppm] 13.19 (s, 2H), 8.84 (d, J = 9.5 Hz, 2H), 7.56–7.49 (m, 3H), 7.43 (d, J = 9.5 Hz, 1H), 7.30–7.25 (m, 2H), 6.45 (s, 1H), 6.09 (ddd, J = 22.0, 10.2, 4.9 Hz, 1H), 5.70 (d, J = 3.6 Hz, 1H), 5.50–5.43 (m, 1H), 5.34–5.27 (m, 1H), 4.98 (d, J = 2.1 Hz, 2H), 4.79 (qd, J = 13.6, 5.0 Hz, 2H), 4.25 (q, J = 7.0 Hz, 2H), 1.28 (t, J = 7.1 Hz, 3H). ^13^C NMR (151 MHz, DMSO-*d*_6_, 298K) δ [ppm] 168.67, 168.40, 168.13, 150.20, 149.80, 148.64, 148.50, 133.62, 126.10, 125.94, 125.92, 125.87, 125.80, 123.93, 123.80, 120.41, 120.30, 119.86, 119.73, 119.37, 119.34, 117.15, 115.04, 114.50, 91.06, 69.36, 65.97, 60.71, 25.99, 21.66, 14.04. ESI-HRMS: m/z calcd for [M-H]^-^ C_32_H_25_O10^-^, 569.1453; found 569.1449 (error = -0.7 ppm).


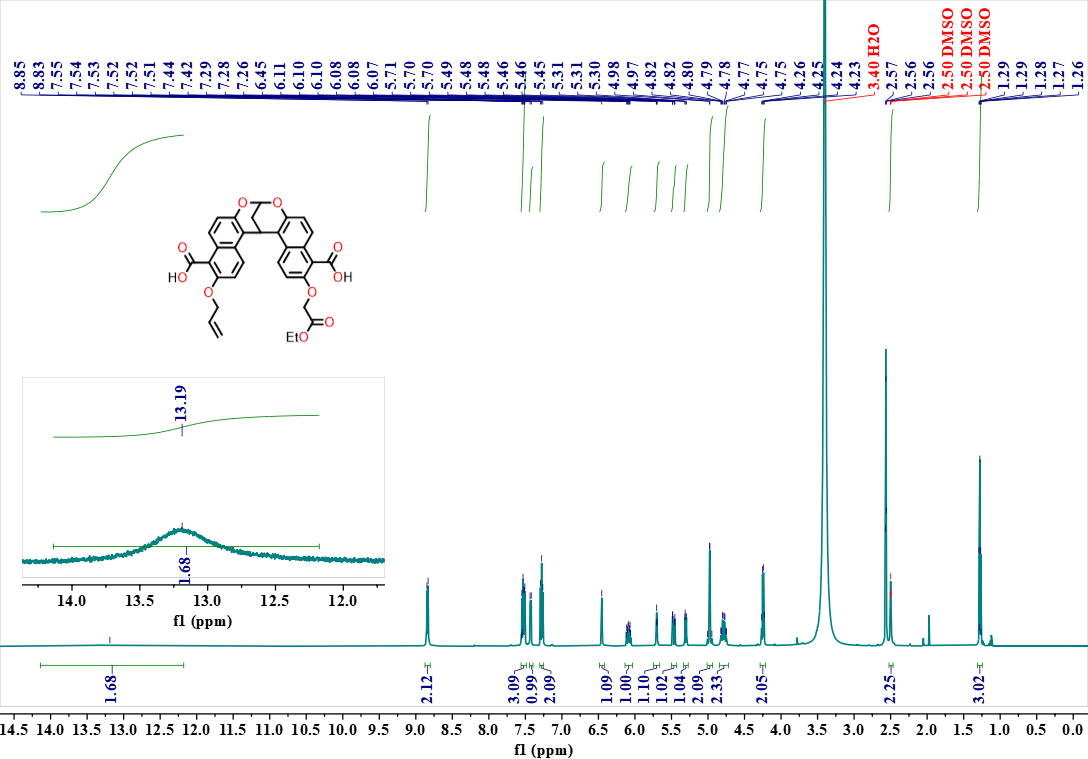


^1^H NMR spectrum (600 MHz, DMSO-*d*_6_, 298 K) of **S5**


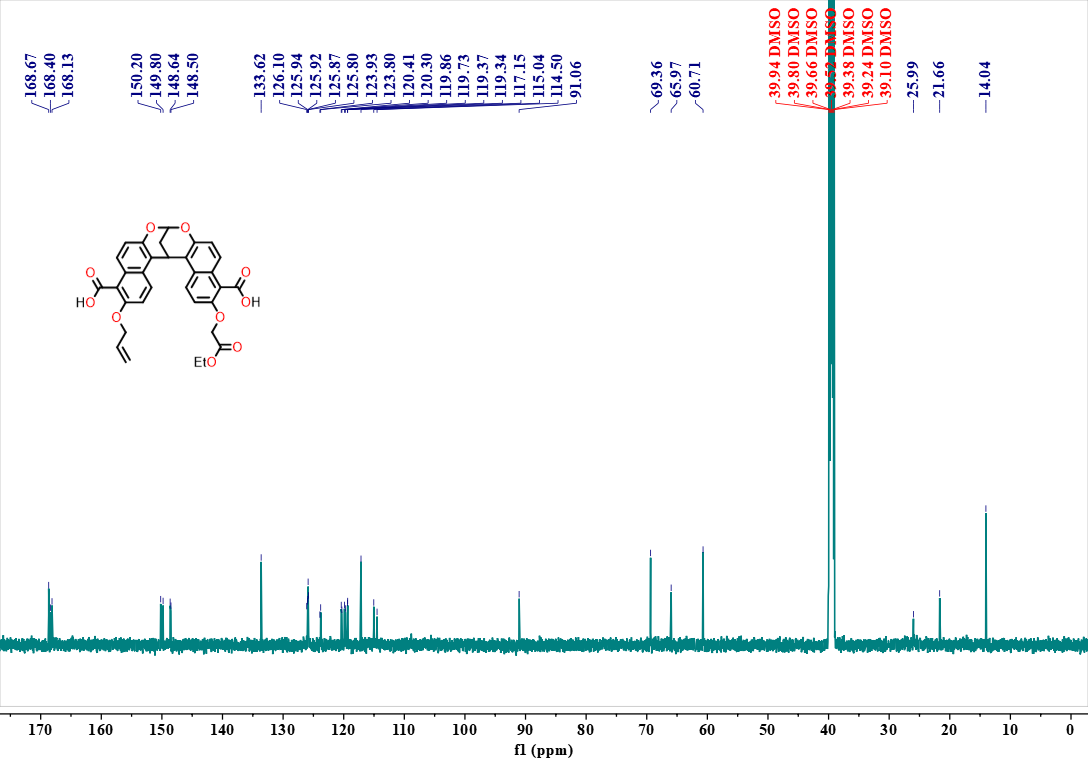


^13^C NMR spectrum (151 MHz, DMSO-*d*_6_, 298 K) of **S5**


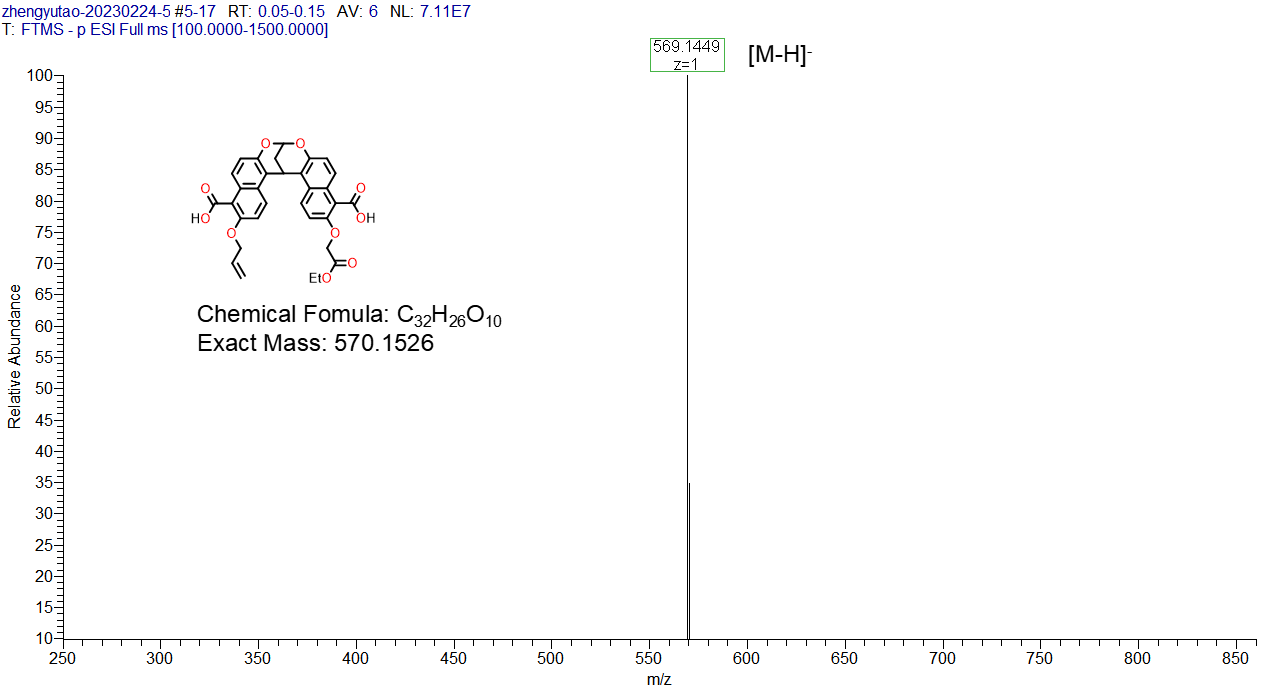


ESI-HRMS mass spectrum of **S5**

**Synthesis of *syn*-S7 and *anti*-S7**

A solution of **S5** (2.0 g, 3.5 mmol) and PyBOP (5.8 g, 11.1 mmol) in dry DMF (60 mL), and a separate solution of **S6** (2.9 g, 3.5 mmol) in dry DMF (60 mL), were simultaneously added via a dual-syringe pump over 8 h to a stirred solution of *N*,*N*-diisopropylethylamine (DIEA, 10.0 mL, 57.3 mmol) and PyBOP (5.8 g, 11.1 mmol) in dry DMF (500 mL) under an argon atmosphere. After stirring at room temperature for an additional 48 h, the majority of the solvent was removed under reduced pressure. The residue was poured into methanol (400 mL), and the resulting precipitate was collected by filtration. The crude product was purified by silica gel column chromatography (CH_2_Cl_2_/MeOH) to afford: ***syn*-S7** as a white solid (0.47 g, 11.9% yield), and ***anti*-S7** as a white solid (0.71 g, 18.1% yield).

***syn*-S7**, white solid, m.p. > 300 ^o^C; ^1^H NMR (500 MHz, CD_2_Cl_2-_*d*_2_, 298 K) δ [ppm] 8.37–8.21 (m, 4H), 7.77 (d, J = 9.2 Hz, 1H), 7.68 (dd, J = 9.2, 3.2 Hz, 2H), 7.62 (d, J = 9.2 Hz, 1H), 7.11–7.02 (m, 6H), 6.99 (dd, J = 12.8, 9.3 Hz, 2H), 6.20 (dt, J = 11.2, 1.6 Hz, 2H), 6.00–5.87 (m, 2H), 5.37 – 5.31 (m, 2H), 5.24 (dd, J = 10.1, 3.3 Hz, 2H), 5.21–5.15 (m, 2H), 5.10 (dd, J = 13.8, 7.6 Hz, 1H), 4.89 (ddd, J = 22.9, 13.8, 1.9 Hz, 2H), 4.71–4.56 (m, 6H), 4.56–4.50 (m, 1H), 4.46–4.38 (m, 1H), 4.13–4.00 (m, 4H), 3.93 (dddd, J = 17.8, 10.8, 7.1, 3.6 Hz, 2H), 2.62 (q, J = 2.9 Hz, 2H), 2.52 (t, J = 2.7 Hz, 2H), 1.13 (dt, J = 17.8, 7.2 Hz, 6H), 1.02 (t, J = 7.2 Hz, 3H). ^13^C NMR (126 MHz, CD_2_Cl_2-_*d*_2_, 298 K) δ [ppm] 169.41, 169.27, 169.13, 166.39, 165.89, 152.42, 152.34, 151.09, 150.33, 150.20, 149.91, 149.81, 149.67, 133.66, 128.73, 128.72, 127.68, 127.60, 127.44, 127.30, 126.74, 126.28, 126.16, 125.85, 124.96, 124.45, 124.32, 123.78, 123.68, 122.59, 122.34, 121.56, 121.08, 120.31, 120.22, 120.18, 120.16, 119.57, 119.53, 119.50, 119.36, 117.32, 114.94, 114.69, 114.63, 113.75, 91.75, 91.53, 70.66, 67.61, 67.40, 66.91, 61.57, 61.50, 61.47, 34.29, 34.02, 26.04, 25.91, 22.92, 22.79, 14.11, 14.04. ESI-HRMS: m/z calcd for [M+H]^+^ C_65_H_57_N_2_O_16_^+^, 1121.3703; found 1121.3697 (error = -0.5 ppm).


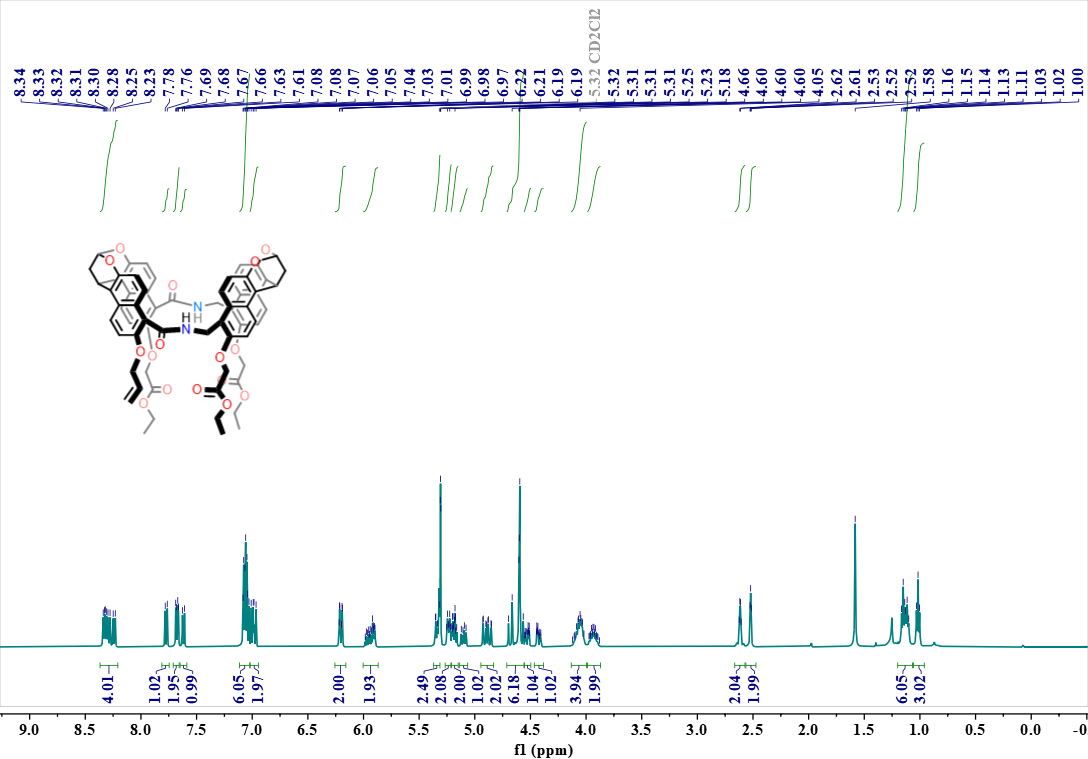


^1^H NMR spectrum (500 MHz, CD_2_Cl_2-_*d*_2_, 298 K) of ***syn*-S7**


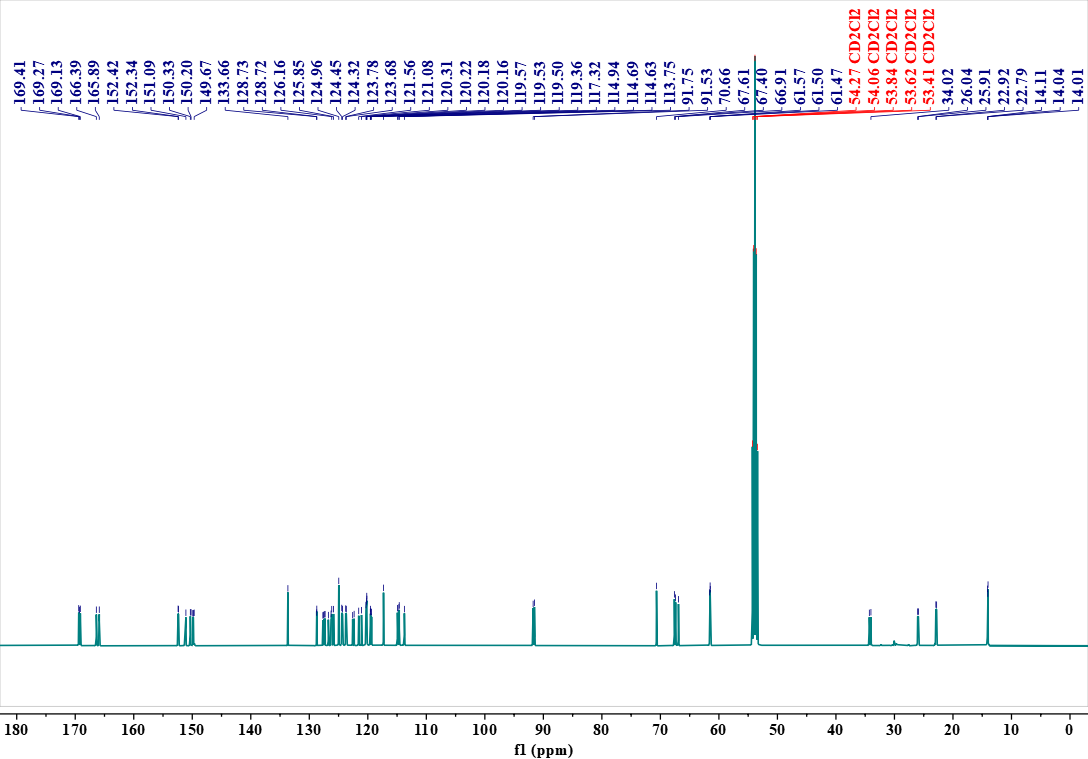


^13^C NMR spectrum (126 MHz, CD_2_Cl_2-_*d*_2_, 298 K) of ***syn*-S7**


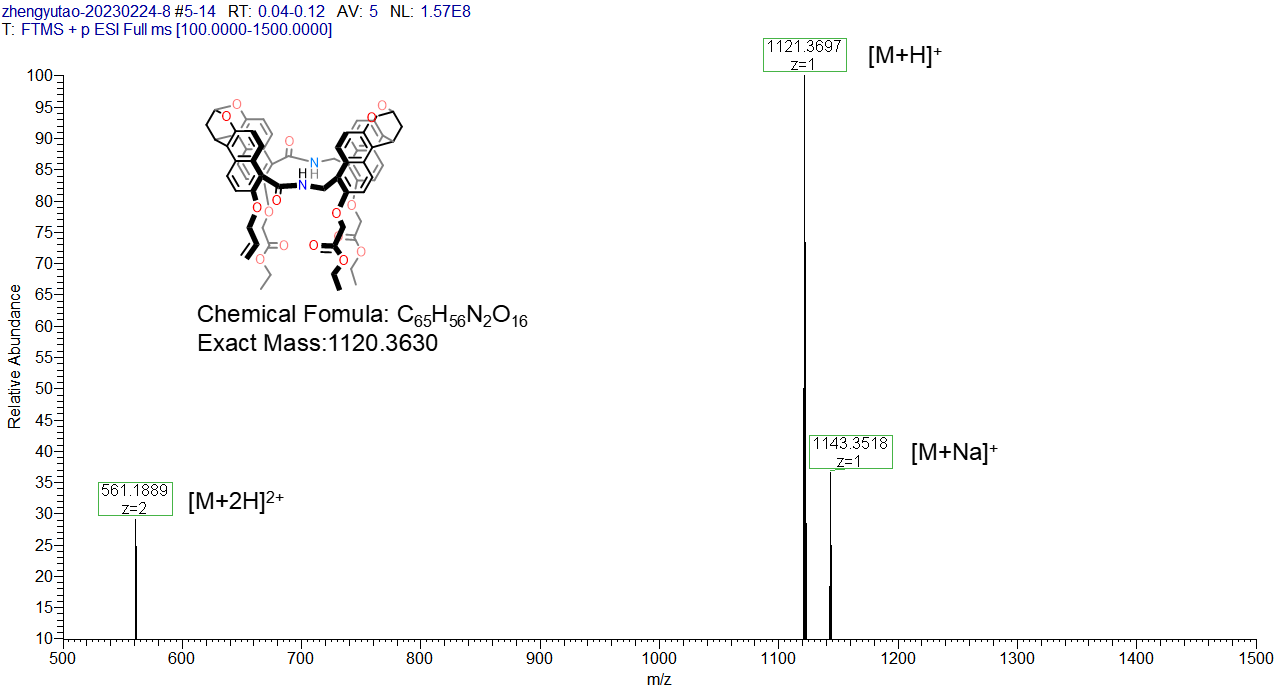


ESI-HRMS mass spectrum of ***syn*-S7**

***anti*-S7**, white solid, m.p. > 300 ^o^C; ^1^H NMR (500 MHz, CD_2_Cl_2-_*d*_2_, 298 K) δ [ppm] 8.42–8.20 (m, 4H), 7.57 (ddd, J = 89.9, 44.5, 9.2 Hz, 4H), 7.12–6.88 (m, 8H), 6.20 (dq, J = 14.5, 1.8 Hz, 2H), 6.09 (ddt, J = 17.2, 10.3, 5.0 Hz, 1H), 5.49 (dq, J = 17.2, 1.7 Hz, 1H), 5.40–5.34 (m, 2H), 5.32–5.26 (m, 4H), 5.25 (d, J = 3.3 Hz, 1H), 4.88 (t, J = 4.0 Hz, 1H), 4.73–4.67 (m, 2H), 4.65–4.58 (m, 7H), 4.48 (ddt, J = 12.9, 5.1, 1.7 Hz, 1H), 4.15 (dqd, J = 10.8, 7.1, 1.4 Hz, 2H), 4.05 (dqd, J = 10.7, 7.1, 3.7 Hz, 2H), 3.95 (dq, J = 10.7, 7.1 Hz, 1H), 3.70 (dq, J = 10.8, 7.1 Hz, 1H), 2.60 (q, J = 4.2, 3.4 Hz, 2H), 2.58–2.52 (m, 2H), 1.17 (td, J = 7.1, 4.4 Hz, 6H), 0.86 (t, J = 7.1 Hz, 3H). ^13^C NMR (126 MHz, CD_2_Cl_2-_*d*_2_, 298 K) δ [ppm] 169.07, 169.03, 168.92, 166.74, 166.26, 152.18, 152.06, 150.87, 150.05, 149.90, 149.76, 149.57, 149.52, 133.70, 128.85, 128.78, 127.39, 127.35, 127.27, 127.18, 126.47, 125.91, 125.86, 125.68, 125.07, 124.98, 124.54, 124.32, 123.60, 123.28, 123.02, 122.50, 120.54, 120.33, 120.15, 120.06, 120.01, 119.99, 119.94, 119.74, 119.53, 119.42, 117.49, 114.77, 114.66, 114.38, 113.84, 91.86, 91.77, 70.34, 67.32, 67.22, 66.92, 61.65, 61.54, 34.86, 34.67, 26.19, 25.95, 22.93, 14.30, 14.03. ESI-HRMS: m/z calcd for [M+H]^+^ C_65_H_57_N_2_O_16_^+^, 1121.3703; found 1121.3701 (error = -0.2 ppm).


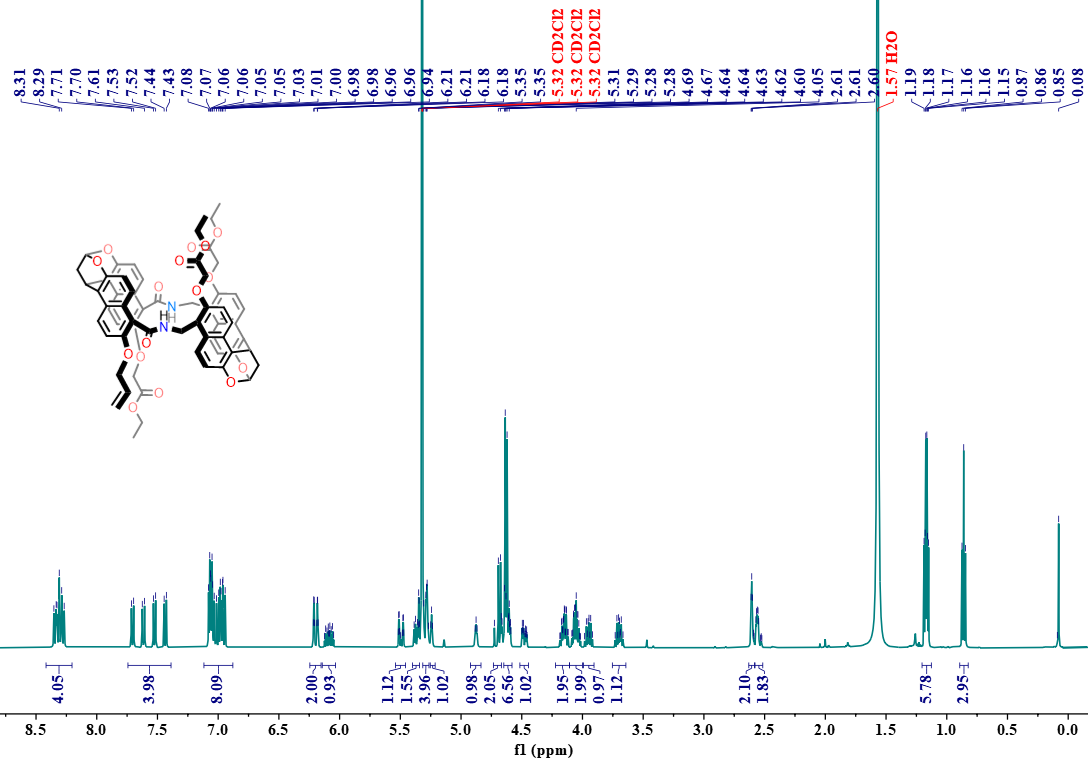


^1^H NMR spectrum (500 MHz, CD_2_Cl_2-_*d*_2_, 298 K) of ***anti*-S7**


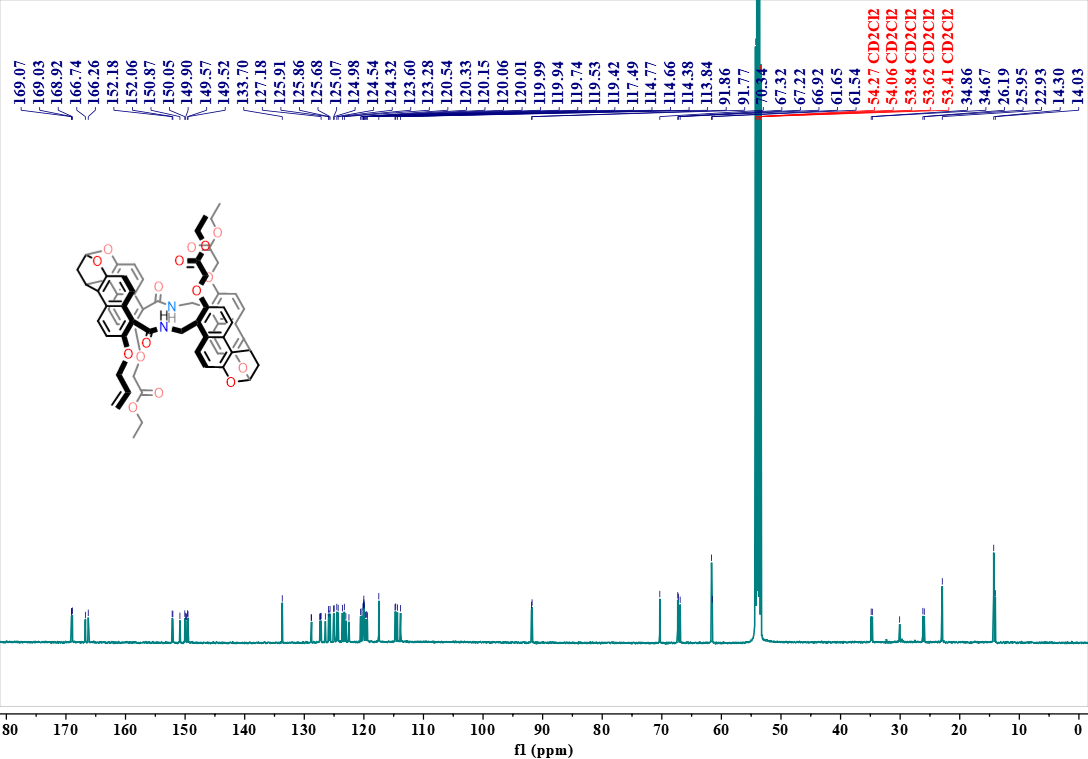


^13^C NMR spectrum (126 MHz, CD_2_Cl_2-_*d*_2_, 298 K) of ***anti*-S7**


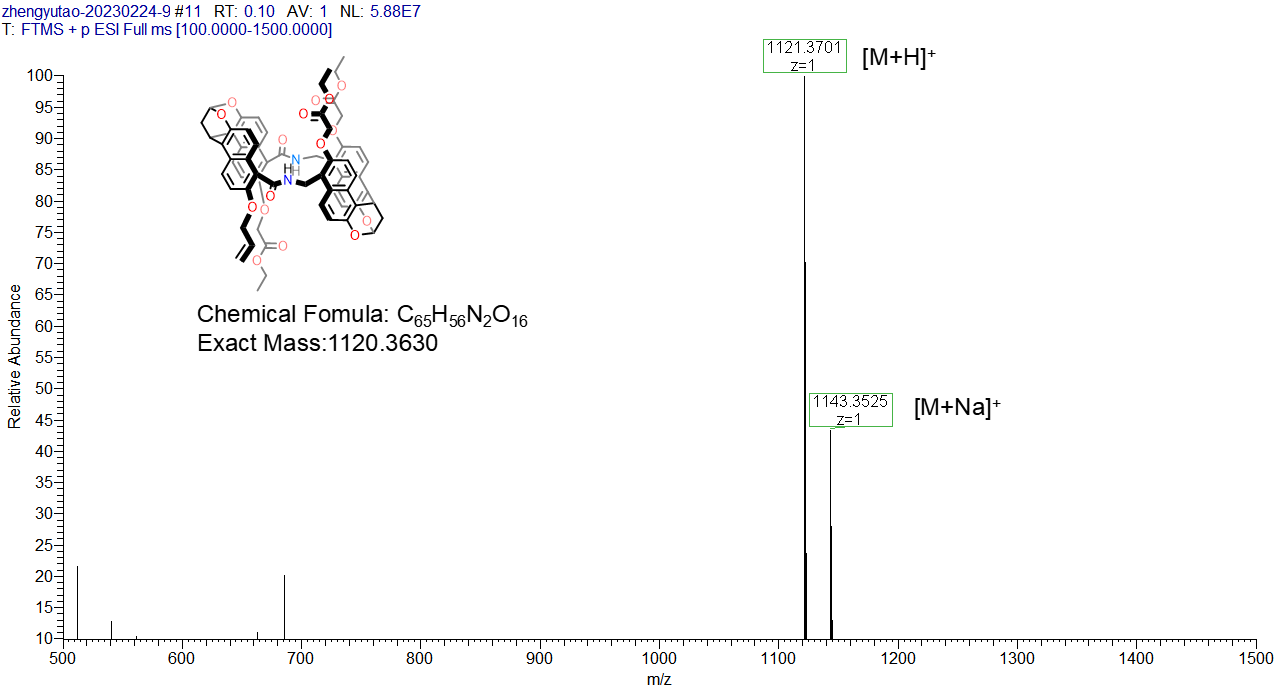


ESI-HRMS mass spectrum of ***anti*-S7**

**Synthesis of *anti-*S8 and water-soluble allyl-functionalized ANT**

***anti-*S7** (0.15 g, 0.13 mmol) was placed in a 500 mL single-necked flask equipped with a magnetic stir bar. Methanol (MeOH, 150 mL), water (H_2_O, 75 mL), and tetrahydrofuran (THF, 150 mL) were added, followed by sodium hydroxide (NaOH, 0.32 g, 8.0 mmol). The resulting mixture was stirred at 50 ^o^C for 12 h. After removal of volatile solvents under reduced pressure, the residue was dissolved in water (75 mL). Concentrated hydrochloric acid (HCl, 2 mL) was carefully added to the solution. Ultrasonication for 30 minutes induced the formation of a white precipitate. The mixture was filtered, and the solid was washed extensively with water. Drying the solid under high vacuum afforded ***anti-*S8** as a white solid (0.13 g, 94% yield).

An aqueous solution of NaOH (3 equiv) was added to ***anti*-S13** to yield **ANT** in aqueous solution. The resulting mixture was freeze-dried to afford **ANT** as a white solid.

***anti-*S8**, white solid, m.p. > 300 ^o^C; ^1^H NMR (400 MHz, DMSO-*d*_6_, 298 K) δ [ppm] 13.16 (s, 2H), 8.57 (dd, J = 9.6, 4.4 Hz, 2H), 8.49 (dd, J = 18.0, 9.5 Hz, 2H), 7.63 (d, J = 9.4 Hz, 1H), 7.54 (d, J = 9.3 Hz, 1H), 7.47 (d, J = 9.2 Hz, 1H), 7.31 (d, J = 9.1 Hz, 1H), 7.28 (s, 1H), 7.17 (dd, J = 28.3, 9.5 Hz, 2H), 7.10–6.94 (m, 6H), 6.84 (d, J = 8.6 Hz, 1H), 6.30 (d, J = 6.4 Hz, 2H), 5.99 (ddt, J = 17.4, 10.0, 4.8 Hz, 1H), 5.57 (d, J = 3.6 Hz, 2H), 5.47–5.34 (m, 2H), 5.26 (dt, J = 10.6, 1.8 Hz, 1H), 5.13 (dd, J = 13.4, 5.2 Hz, 1H), 4.88 (d, J = 16.8 Hz, 1H), 4.77– 4.60 (m, 5H), 4.55 (dd, J = 13.6, 5.0 Hz, 1H), 4.45–4.27 (m, 3H). ^13^C NMR (151 MHz, DMSO-*d*_6_, 298 K) δ [ppm] 171.19, 170.45, 165.91, 165.43, 151.67, 151.50, 150.29, 149.68, 148.96, 148.46, 148.24, 148.15, 134.09, 128.98, 128.67, 126.96, 126.68, 126.60, 126.57, 126.10, 125.86, 125.83, 125.22, 124.96, 124.91, 124.27, 124.17, 123.10, 123.05, 122.41, 119.66, 119.62, 119.53, 119.42, 119.32, 119.21, 119.05, 118.84, 118.80, 118.44, 116.56, 114.96, 114.21, 114.17, 113.85, 90.74, 90.70, 69.46, 66.03, 65.70, 65.66, 40.06, 33.82, 33.13, 25.58, 25.22, 21.32, 21.27. ESI-HRMS: m/z calcd for [M-H]^-^ C_59_H_43_N_2_O_16_^-^, 1035.2624; found 1035.2606 (error = -1.4 ppm).


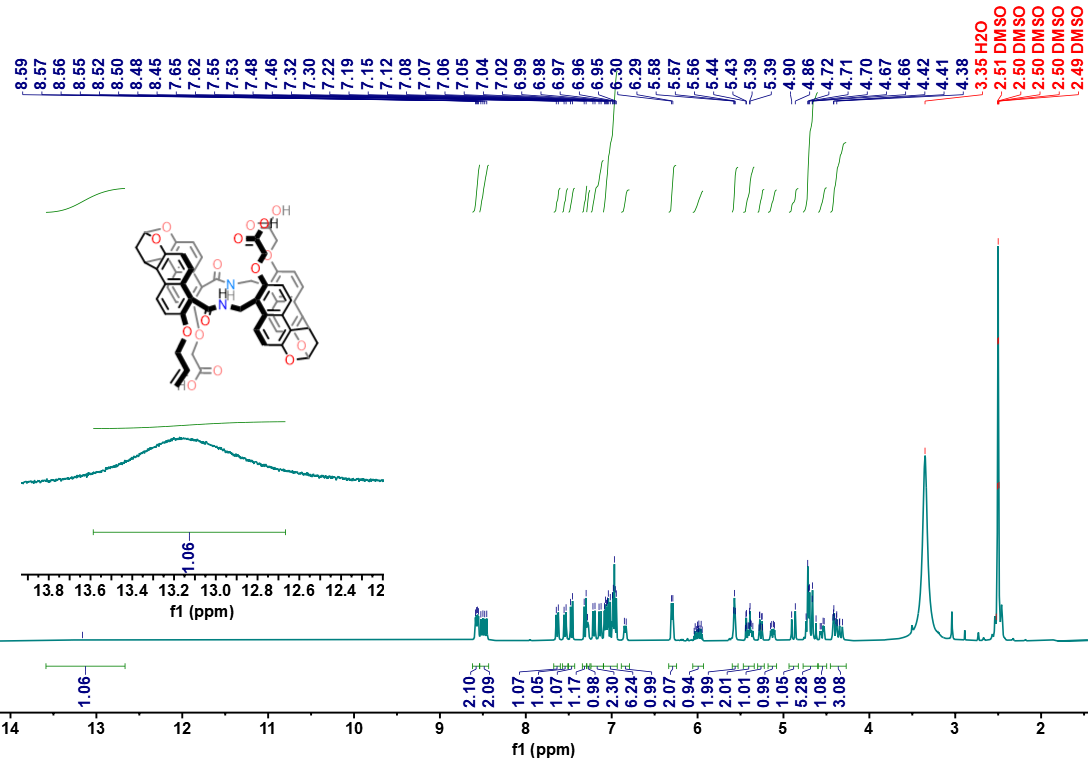


^1^H NMR spectrum (400 MHz, DMSO-*d*_6_, 298 K) of ***anti-*S8**


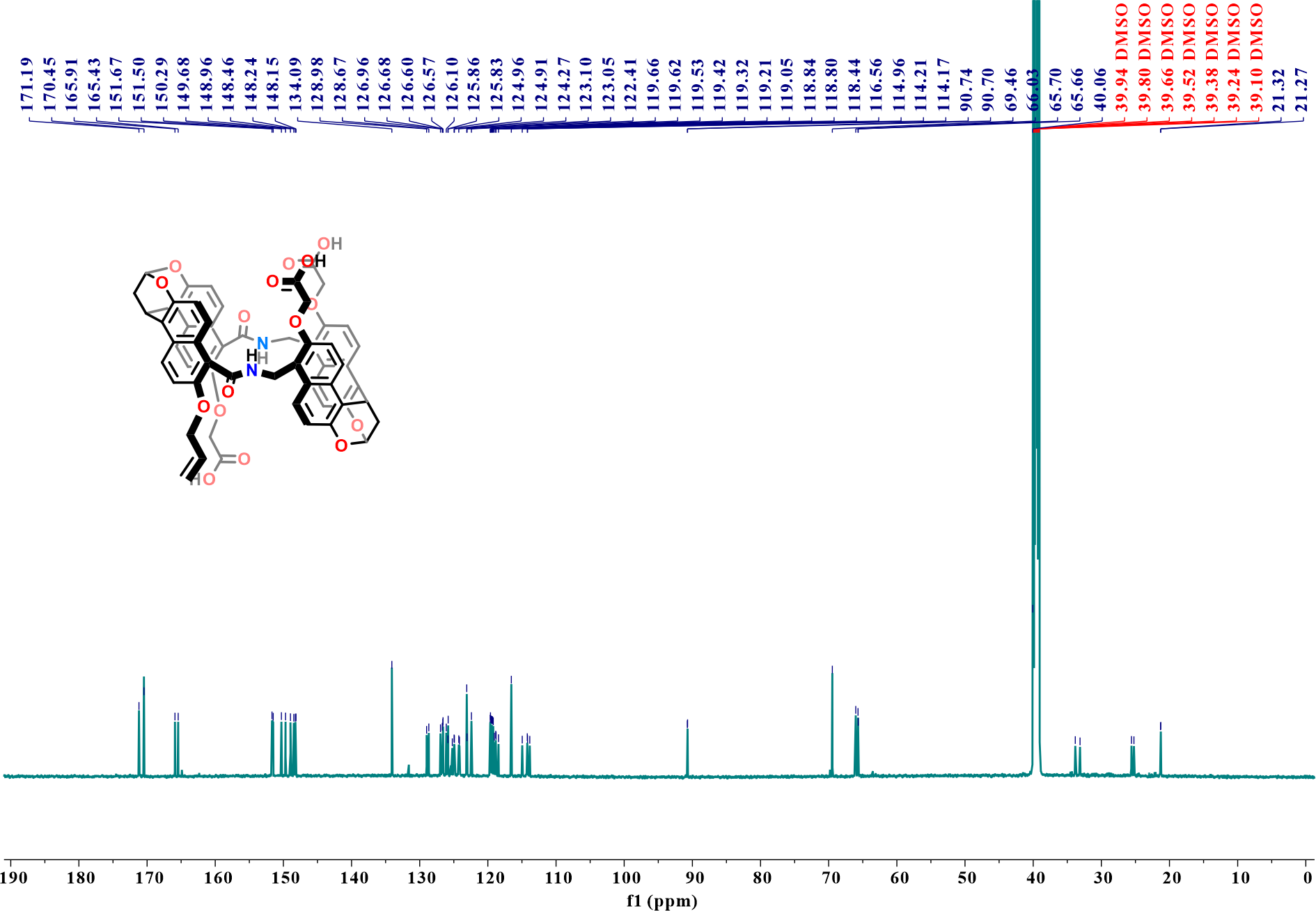


^13^C NMR spectrum (151 MHz, DMSO-*d*_6_, 298 K) of ***anti-*S8**


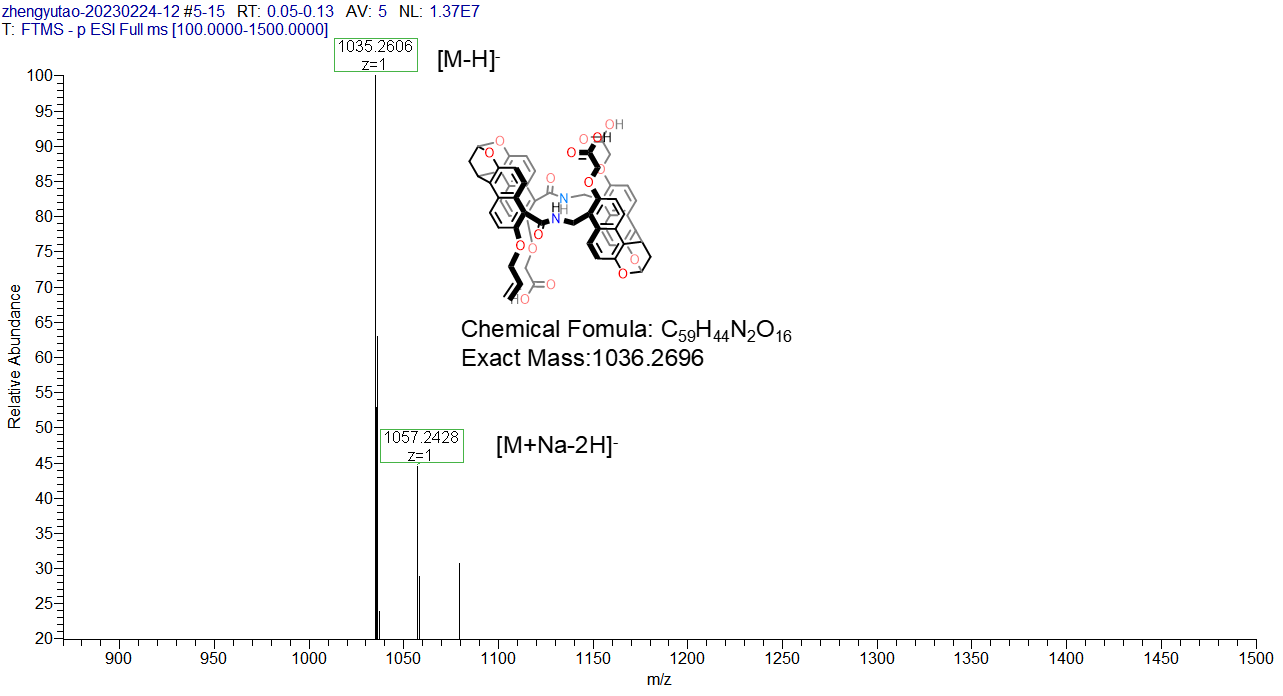
ESI-HRMS mass spectrum of ***anti-*S8**

1. **2 D NMR of the Configurational Isomers of S7**


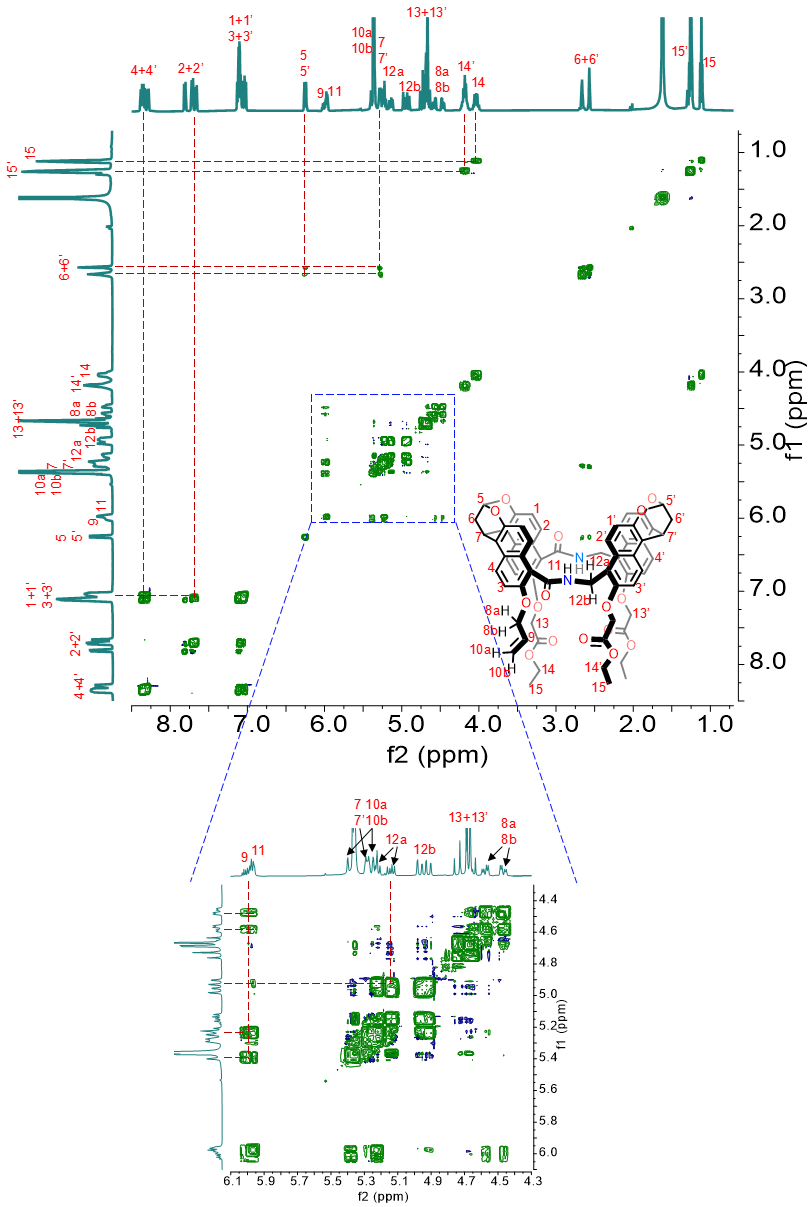


^1^H, ^1^H-COSY NMR spectrum (500 MHz, CD_2_Cl_2-_*d*_2_, 298 K) of ***syn*-S7**


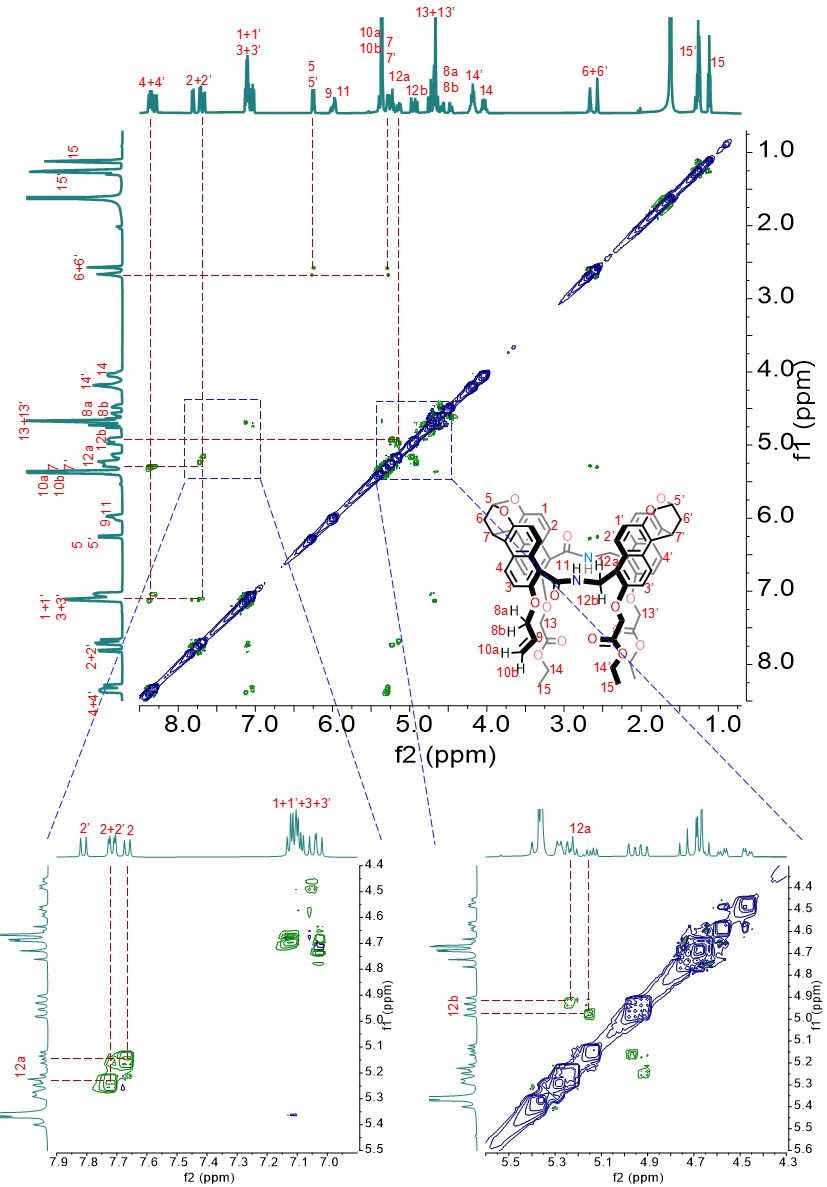


^1^H, ^1^H-ROESY NMR spectrum (500 MHz, CD_2_Cl_2-_*d*_2_, 298 K) of ***syn*-S7**


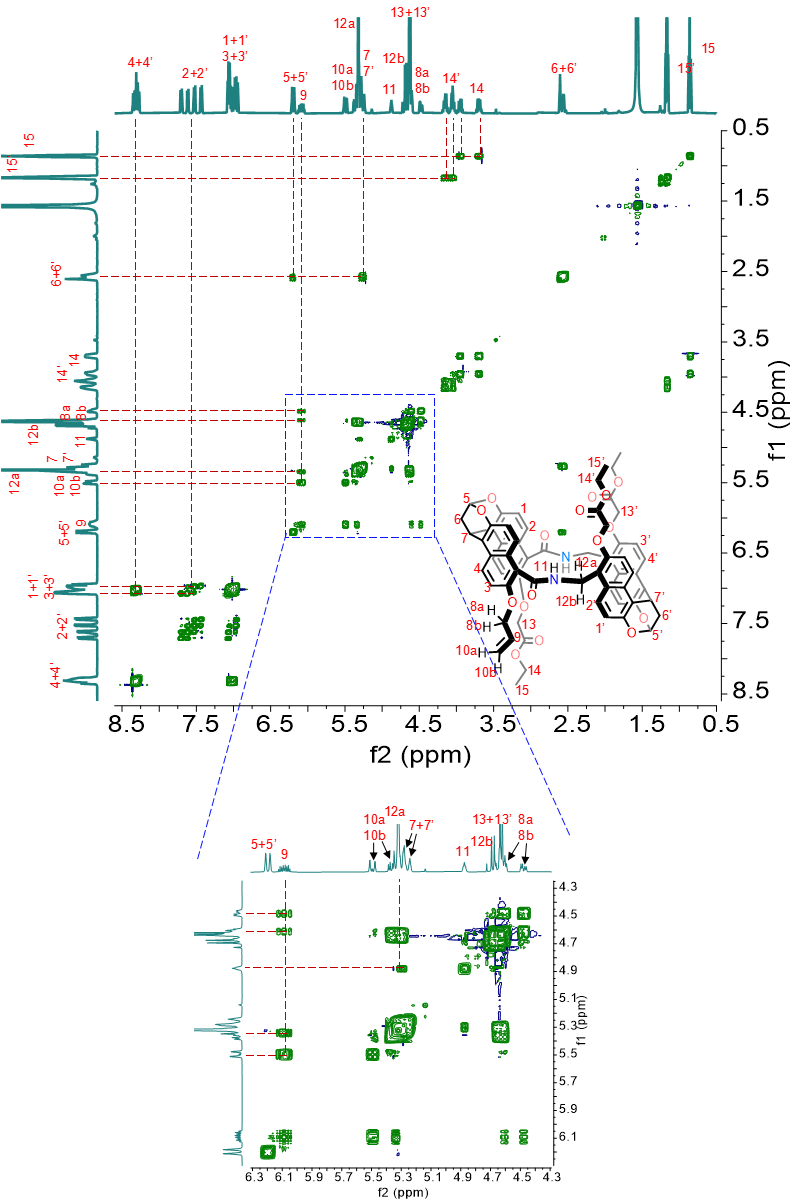


^1^H, ^1^H-COSY NMR spectrum (500 MHz, CD_2_Cl_2-_*d*_2_, 298 K) of ***anti*-S7**


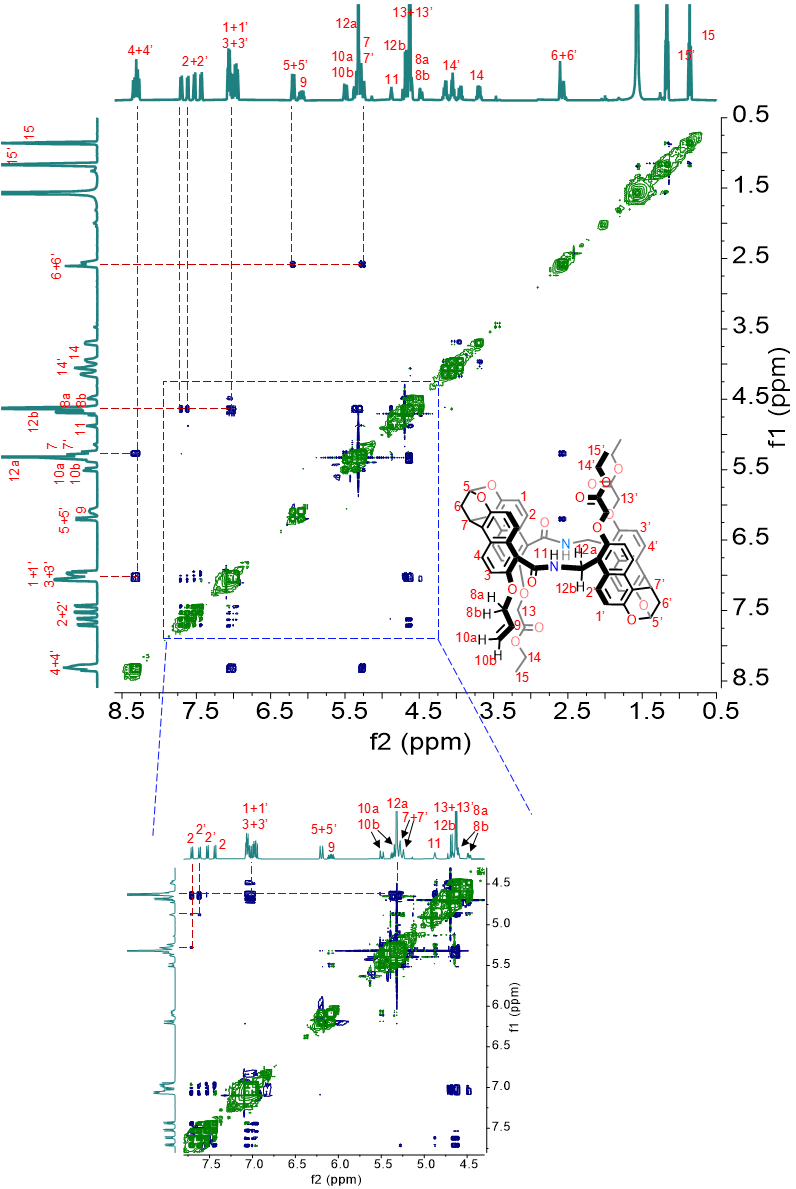


^1^H, ^1^H-ROESY NMR spectrum (500 MHz, CD_2_Cl_2-_*d*_2_, 298 K) of ***anti*-S7**

1. **X-Ray Single Crystallography of *syn*-S7 and *anti*-S7**

Suitable single crystal of ***syn*-S7** and ***anti*-S7** were was successfully obtained by slow evaporation of its saturated solution in CH_2_Cl_2_/MeOH.

Single crystal X-ray data were collected on a Bruker D8 VENTURE with Ga K*_α_* radiation (*λ* = 1.34139 Å) at 193 K or 100 K. The structures were solved by intrinsic phasing methods (SHELXT) and refined by full-matrix least squares on *F*^2^ using SHELXL^[4]^ in the OLEX2 program package ^[5]^. All non-hydrogen atoms were refined with anisotropic thermal parameters and the hydrogen atoms were fixed at calculated positions and refined by a riding mode. SQUEEZE routine implemented on PLATON^[6]^ was used to remove electron densities corresponding to disordered solvent molecules in the crystal data.


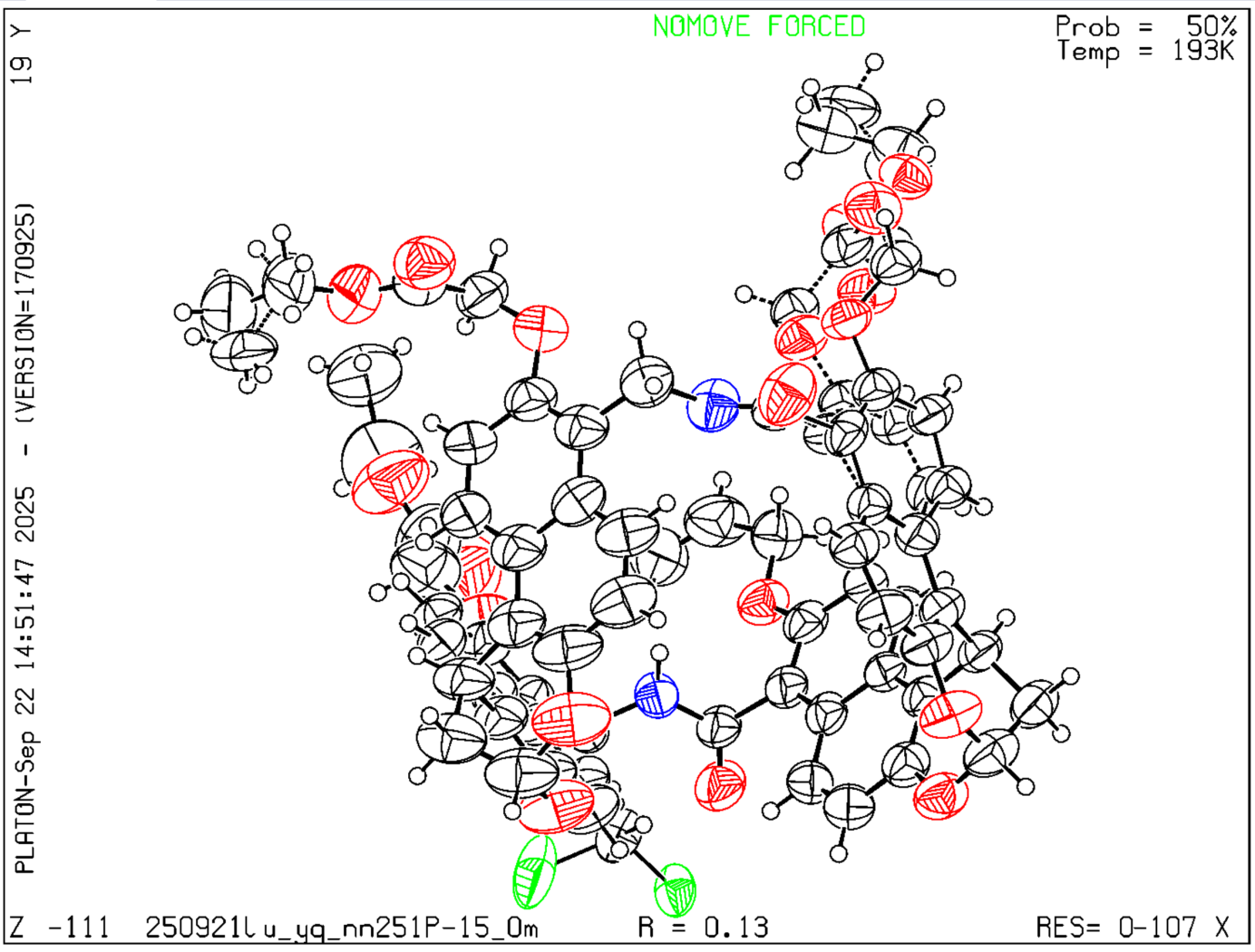


Oak Ridge thermal ellipsoid plot of ***syn*-S7**

PLAT340_ALERT_3_B Low Bond Precision on C-C Bonds ............... 0.01256 Ang.

**Response**: The crystal has too weak diffraction to obtain high resolution data.

**Table S1** Crystal data and structure refinement for ***syn*-S7**

| entry | ***syn*-S7** |
| --- | --- |
| Empirical formula | C_65.5_H_57_ClN_2_O_16_ |
| Formula weight | 1163.58 |
| Temperature/K | 193.00 |
| Crystal system | triclinic |
| Space group | *P*-1 |
| *a*/Å | 13.9834(13) |
| *b*/Å | 14.2787(13) |
| *c*/Å | 17.8058(16) |
| *α*/° | 110.269(3) |
| *β*/° | 94.708(3) |
| *γ*/° | 112.665(3) |
| Volume/Å^3^ | 2980.5(5) |
| *Z* | 2 |
| *ρ*_calc_g/cm^3^ | 1.297 |
| *μ*/mm^‑1^ | 0.778 |
| *F*(000) | 1218.0 |
| Reflections collected | 31457 |
| Independent reflections | 10861 [*R*_int_ = 0.0706, *R*_sigma_ = 0.0765] |
| Data/restraints/parameters | 10861/2425/891 |
| Goodness-of-fit on *F*^2^ | 1.097 |
| Final *R* indexes [*I*>=2*σ* (*I*)] | *R*_1_ = 0.1257, *wR*_2_ = 0.3146 |
| Final R indexes [all data] | *R*_1_ = 0.1919, *wR*_2_ = 0.3509 |
| CCDC number | 2520355 |


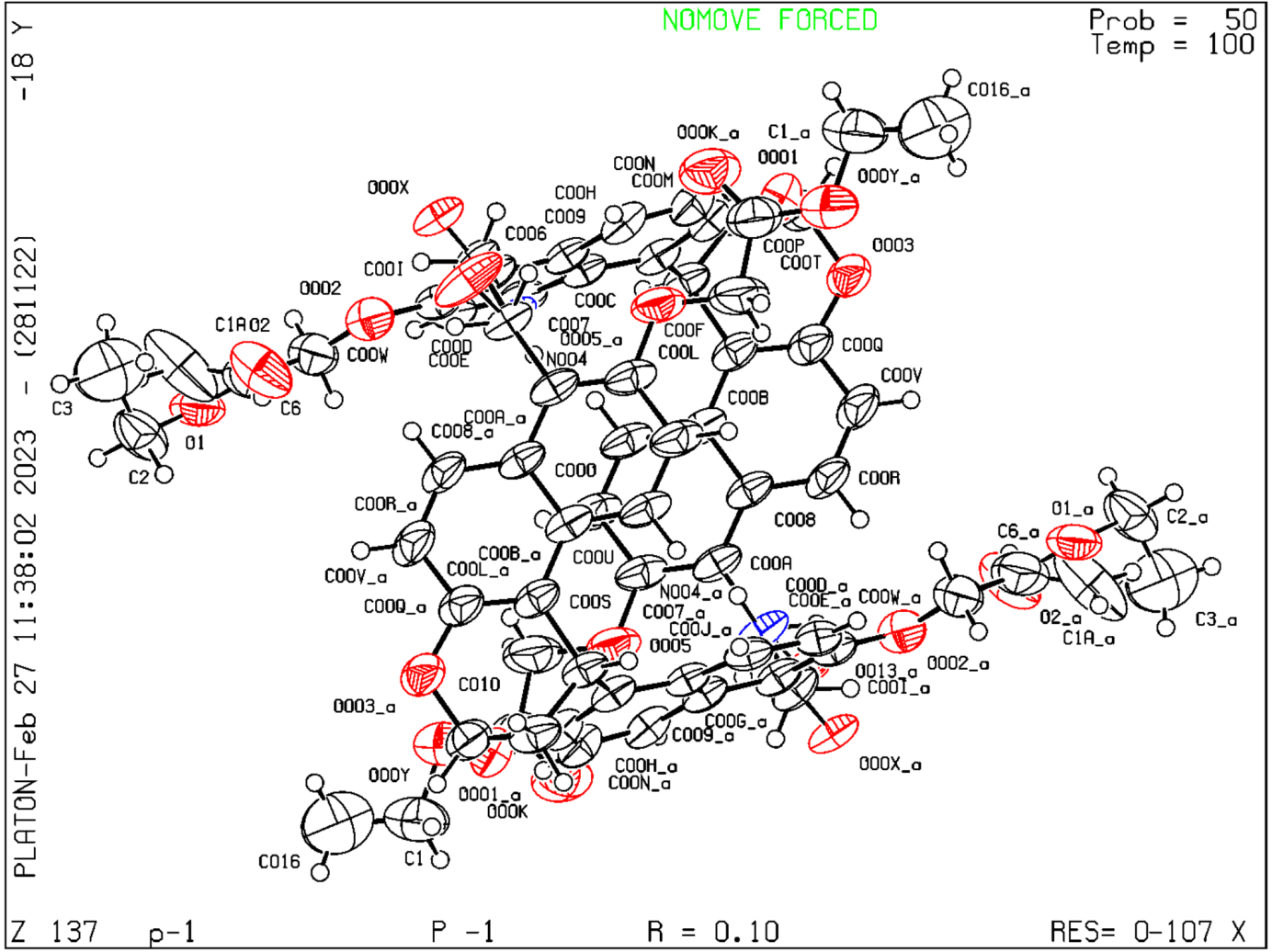


Oak Ridge thermal ellipsoid plot of ***anti*-S7**

DIFMN02_ALERT_2_B The minimum difference density is < -0.1*ZMAX*1.00

_refine_diff_density_min given = -0.872

Test value = -0.800

PLAT097_ALERT_2_B Large Reported Max. (Positive) Residual Density 0.86 eA-3

PLAT098_ALERT_2_B Large Reported Min. (Negative) Residual Density -0.87 eA-3

**Response**: The crystal has too weak diffraction to obtain high resolution data.

**Table S2** Crystal data and structure refinement for ***anti*-S7**

| entry | ***syn*-S7** |
| --- | --- |
| Empirical formula | C_65_H_56_N_2_O_16_ |
| Formula weight | 1121.11 |
| Temperature/K | 100.00 |
| Crystal system | triclinic |
| Space group | *P*-1 |
| *a*/Å | 9.8396(7) |
| *b*/Å | 11.8315(8) |
| *c*/Å | 12.0001(8) |
| *α*/° | 96.379(3) |
| *β*/° | 99.672(3) |
| *γ*/° | 95.594(3) |
| Volume/Å^3^ | 1358.98(16) |
| *Z* | 1 |
| *ρ*_calc_g/cm^3^ | 1.370 |
| *μ*/mm^‑1^ | 0.523 |
| *F*(000) | 588.0 |
| Reflections collected | 27094 |
| Independent reflections | 5540 [*R*_int_ = 0.0513, *R*_sigma_ = 0.0407] |
| Data/restraints/parameters | 5540/55/391 |
| Goodness-of-fit on *F*^2^ | 1.953 |
| Final *R* indexes [*I*>=2*σ* (*I*)] | *R*_1_ = 0.1371, *wR*_2_ = 0.4349 |
| Final R indexes [all data] | *R*_1_ = 0.1642, *wR*_2_ = 0.4667 |
| CCDC number | 2520361 |

1. **Method**

**^1^H NMR Titrations**

For ^1^H NMR titrations, a 5.0×10^-4^ M solution of the host was prepared in D_2_O. This solution (0.5 mL) was placed in a NMR tube. The sample was then titrated with a solution of guest. For all the host-guest pairs, chemical exchange is fast on the NMR time scale. When determining binding constants, NMR titrations were performed by adding guests to the solution of host with a fixed concentration of host. Nonlinear curve-fitting method was then used to obtain the binding constant through the following equation (1):

δ=I_0_+(((δ_GH_-I_0_)/2)/[H]_0_)*([H]_0_+[G]_0_+1/Ka-sqrt(([H]_0_+[G]_0_+1/Ka)^2-4*[H]_0_*[G]_0_)) (1)

**Preparation of Hydrogels**


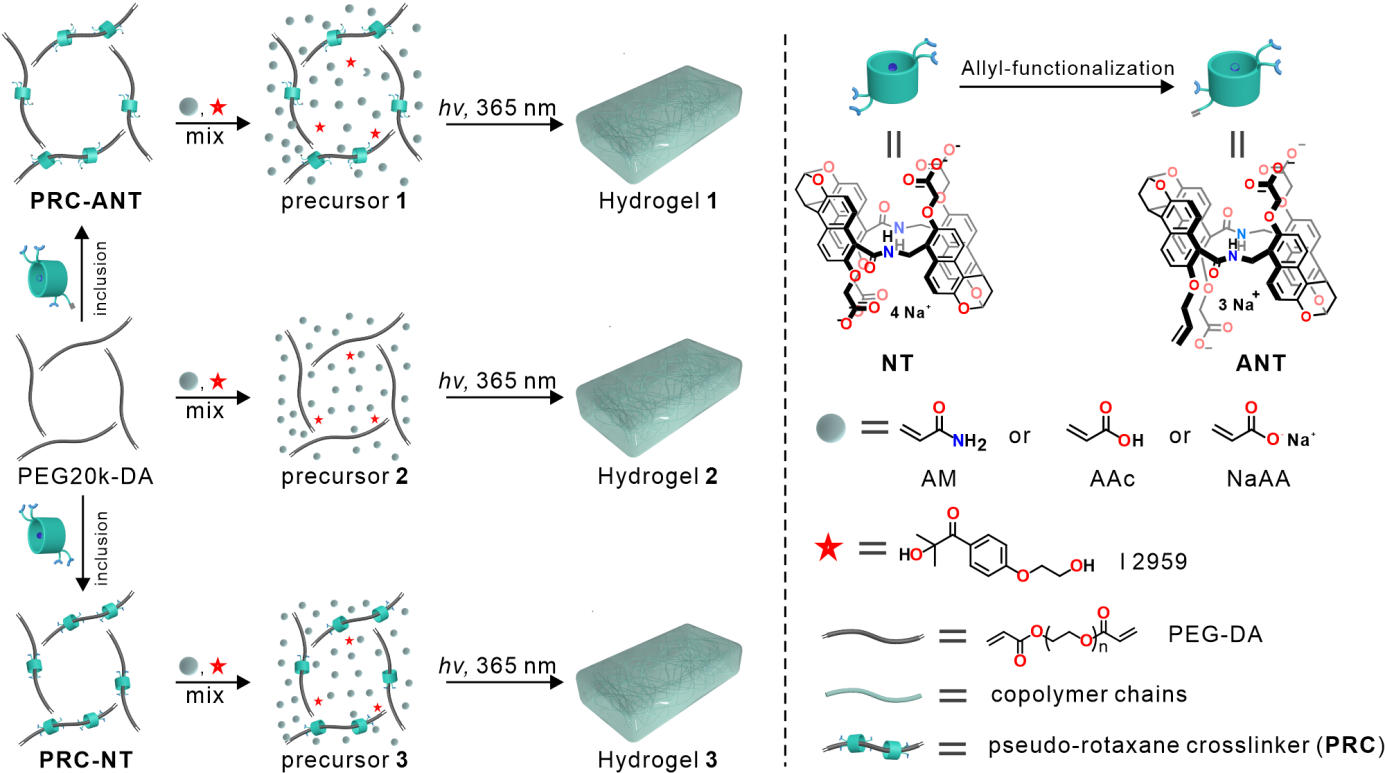


***Scheme S2*.** Preparation procedures of hydrogels.

As shown in Scheme S2, the hydrogels was typical prepared by two steps.A representative procedure is described as below. Firstly, PEG_20k_-DA and **ANT** were dissolved in a certain amount of deionized water at a molar ratio of 1:2. The mixture was stored in a refrigerator for 24 h to yield a threaded pseudo-polyrotaxane crosslinker (**PRC-ANT**) stock solution, with the **PRC-ANT** concentration fixed at 0.1 g/mL. Subsequently, the total volume of the gel precursor solution was adjusted to 400 μL. To this, predetermined amounts of acrylamide (AM), acrylic acid (AAc), sodium acrylate (NaAA), 0.3 mg of photoinitiator Irgacure 2959, and a specific volume of the **PRC-ANT** stock solution were added. Deionized water was used to bring the mixture to the final volume. The precursor solution was then pipetted into a custom polytetrafluoroethylene (PTFE) mold, covered with a 1 mm thick quartz plate to prevent moisture evaporation, and exposed to UV light (365 nm, 72 W) for 30 min to complete polymerization..

To optimize the hydrogel formulation, samples with different monomer ratios and varying contents of the functionalized pseudo-polyrotaxane crosslinker (**PRC-ANT**) were prepared following the standard procedure. In parallel, control hydrogels were synthesized for comparative analysis. Specifically, hydrogel **2** was prepared using a conventional, non-threaded PEG_20k_-DA crosslinker instead of **PRC-ANT**. Hydrogel **3** was fabricated with a pseudo-polyrotaxane crosslinker based on the non-functionalized, classic naphthotube (**PRC-NT**). All other preparation steps and parameters remained unchanged.

**Mechanical Property Characterization**

The mechanical performance of the hydrogels was evaluated using an electromechanical universal testing machine. Hydrogel samples were prepared into rectangular strips measuring 40 mm × 4 mm × 2 mm (length × width × thickness). A gauge length of 10 mm was marked and measured with a vernier caliper prior to testing. Uniaxial tensile tests were performed at a constant crosshead speed of 100 mm/min using a 20 N load cell. Each test condition was repeated three times, and the average value along with the standard deviation is reported. *The nominal stress* was calculated by dividing the instantaneous force by the original cross-sectional area, and *the nominal strain* was determined as the elongation divided by the original gauge length. *Young's modulus* was obtained from the slope of the linear region (20%–50% strain) of the stress-strain curve. *Toughness* was defined as the area under the stress-strain curve up to fracture, calculated via equation (2). *The fracture energy* was derived from the integrated area under the tensile curve and the initial specimen length, as given in equation (3).

$\text{Toughness = }\int_{\text{0}}^{\text{ε}} \text{σdε}$ equation (2)

$\text{Fracture energy = }\text{l}\int_{\text{0}}^{\text{ε}} \text{σdε}$ equation (3)

Where ε denotes the tensile fracture strain, σ corresponds to the tensile stress, and l represents the gauge length of the sample.

**Cyclic Tensile Experiments**

The fatigue resistance and energy dissipation behavior of the hydrogel were evaluated via cyclic tensile testing. All tests were conducted in a controlled environment at 25 °C and 50% relative humidity. The hydrogel was first subjected to 100 loading-unloading cycles at a constant crosshead speed of 50 mm/min to a strain of 100%. Subsequently, cyclic tests were performed at progressively increasing maximum strain levels of 50%, 100%, 200%, 400%, 600%, and 800%, starting from a pre-strain of 50% and at the same speed of 50 mm/min. Each test condition was repeated three times, and the results are presented as the mean ± standard deviation. The *dissipated energy* per cycle was quantified by the area of the hysteresis loop. The *hysteresis ratio* was calculated using the following formula (4).

$\text{Hysteresis ratio(\%) = }\frac{\text{A}_{\text{c}}}{\text{A}_{\text{l}}}\text{ × 100\%}$ equation (4)

Where A_c_ denotes the area between the loading and unloading curves in a cycle (i.e., the hysteresis loop area), and A_l_ represents the area under the loading curve.

**Adhesive Performance**

The adhesion performance of the hydrogel to various substrates was evaluated via 180° lap-shear tests using an electromechanical universal testing machine. A square gel sample (8 mm × 8 mm × 1 mm) was placed between two identical substrate panels. A contact pressure of approximately 20 kPa (applied by a 2 kg weight ) was maintained for 15 minutes to ensure conformal contact. Subsequently, the assembly was stretched at a rate of 20 mm/min until interfacial failure occurred. The *adhesive shear strength* was calculated by dividing the maximum tensile force at the point of joint failure by the initial bonded area (64 mm²). Each measurement was repeated three times, and the results are reported as the mean ± standard deviation. A schematic of the test setup is illustrated in Scheme S3.

**
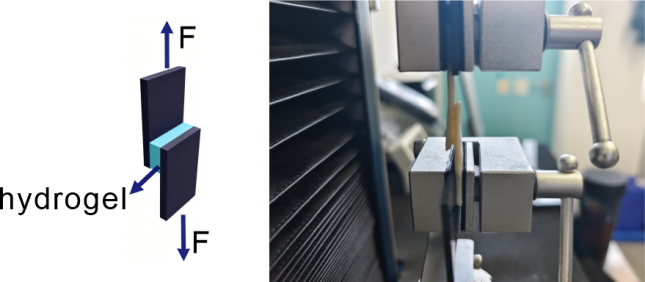
**

***Scheme S3*.** Schematic illustration of 180^o^ lap-shear test.

**Puncture Experiment**

To assess the penetration resistance, hydrogel discs (20 mm diameter, 1 mm thickness) were subjected to puncture testing. The test utilized a 1 mm diameter needle with a hemispherical tip, mounted on the load frame of a universal testing machine. The sample was securely clamped in a custom specimen holder featuring a 10 mm central opening. The needle was then advanced at 10 mm/min until complete puncture, while force and displacement data were continuously acquired. The test was repeated three times, and results are presented as mean ± standard deviation. The experimental configuration is illustrated in Scheme S4.


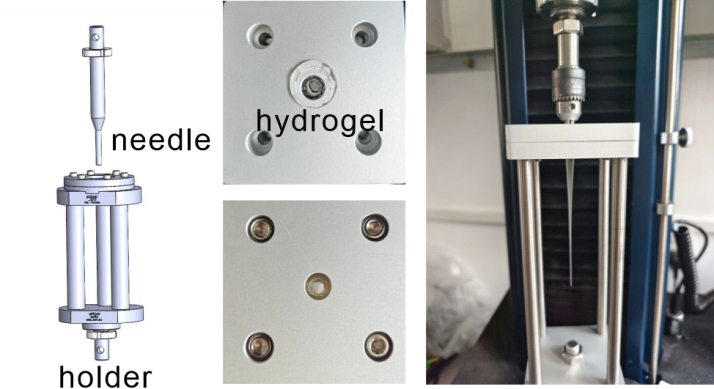


***Scheme S4*.** Schematic illustration of puncture experiment.

**Conductivity Measurements**

The electrical conductivity of the hydrogels was measured using a standard four-point probe method coupled with a digital source meter. Circular gel samples with a diameter of 2 cm and a thickness of 1 mm were prepared for testing. The probes, with a fixed spacing of 1.59 mm, were placed in contact with the sample surface. Measurements were performed under a constant bias voltage of 2 V with an output current of 100 μA. Each sample was tested three times, and the results are presented as the mean ± standard deviation.

**SEM Characterization**

To prepare the sample for SEM observation, hydrated hydrogels were freeze-dried using a freeze dryer (Alpha 2-4 LD plus) and coated with Pt using an ion-sputtering coater (Q150TES, Quorum). To verify the three-dimensional structures of hydrogels, the cross-sectional microstructures were visualized by the field emission SEM (Regulus 8100) at an acceleration voltage of 15 kV.

**Sensing Performance Measurement**

The electromechanical sensing performance of the hydrogel was characterized using a Keithley 2450 digital source meter integrated with a universal testing machine. A rectangular hydrogel sample (40 mm × 4 mm × 2 mm) was prepared for testing. Conductive copper tape electrodes were attached at both ends of the sample and connected to the source meter via alligator clips and copper wires. A constant bias voltage of 2 V was applied throughout the test. The sample was stretched at a constant rate of 100 mm/min. The *relative change in resistance (ΔR/R_0_)* was monitored in real time by the source meter and calculated according to formula (5). The *strain sensitivity* was quantified by the gauge factor (GF), derived from the slope of the ΔR/R₀ versus strain curve and calculated using equation (6). All tests were performed in triplicate, and results are reported as mean ± standard deviation.

$\text{ΔR}/{\text{R}_{\text{0}}}\text{(\%) = }\frac{\text{(R-}\text{R}_{\text{0}}\text{)}}{\text{R}_{\text{0}}}\text{ ×100\%}$ equation (5)

$GF = \frac{{\Delta R}/{R_{0}}}{\varepsilon}$ equation (6)

where R and R_0_ are the instantaneous and initial electrical resistances of the hydrogel, respectively, and ε is the applied tensile strain.

**Cyclic stability of the hydrogel**

During the test, use conductive copper tape to connect the hydrogel to the digital source meter. Let the hydrogel be stretched cyclically at a movement speed of 50 mm/min and under a strain of 50% for 1000 cycles. During the test, use a humidifier to maintain the air humidity above 80%, to prevent the gel moisture from drying out. The room temperature should be controlled at 25℃.

**Response time of the hydrogel sensor**

During the test, stretch the hydrogel at a rate of 50 mm/min to a strain of 50%, maintain this strain for 5 seconds, then return to a strain of 0%, and record the change in the relative resistance value of the hydrogel. During the process of gel loading and unloading, the time difference between the electrical signal and the strain curve represents the response time of the hydrogel.

For the remaining measurements of the sensing signals, unless otherwise specified, the stretching rate of the hydrogel will be uniformly 50 mm/min.

**Detection of human movement signals**

Use transparent tape to fix the hydrogel on the human joint, and use crocodile clips to connect the hydrogel fixed on the joint to the Keithley 2450 digital source meter. Measure the relative resistance value of the hydrogel as it changes with the movement of the human joint, and obtain the motion electrical signal of the human joint. The test voltage is always 2V. All the tests were conducted three times, and then the average values and standard deviations were recorded.

**Epidermal electrophysiological signal detection**

The measurement method for the epidermal electrophysiological signal detection is based on the previous research work^[7~9]^. The electrodes were fabricated using circular gel pieces with a diameter of 2 cm and a thickness of 1 mm. Electrocardiogram (ECG) and electromyogram (EMG) were recorded using a multi-channel biological signal acquisition and processing system (RM6240XC, Chengdu Instrument Factory, China). The calculation method for the signal-to-noise ratio (SNR) of electrocardiogram (ECG) and electromyogram (EMG) is as follows (6).

$\text{SNR(dB) }\text{= 20 × log(}\text{Signal}/\text{Noise}\text{)}$ equation (8)

For EMG, the signal is defined as the average absolute amplitude of the EMG wave, and the noise is determined as the average absolute amplitude of the static EMG signal. For ECG, the signal is calculated as the average amplitude of the consecutive ten R waves, and the noise is quantified by the average amplitude within a 0.1 second window before the P wave. All the tests were conducted three times, and then the average values and standard deviations were recorded.


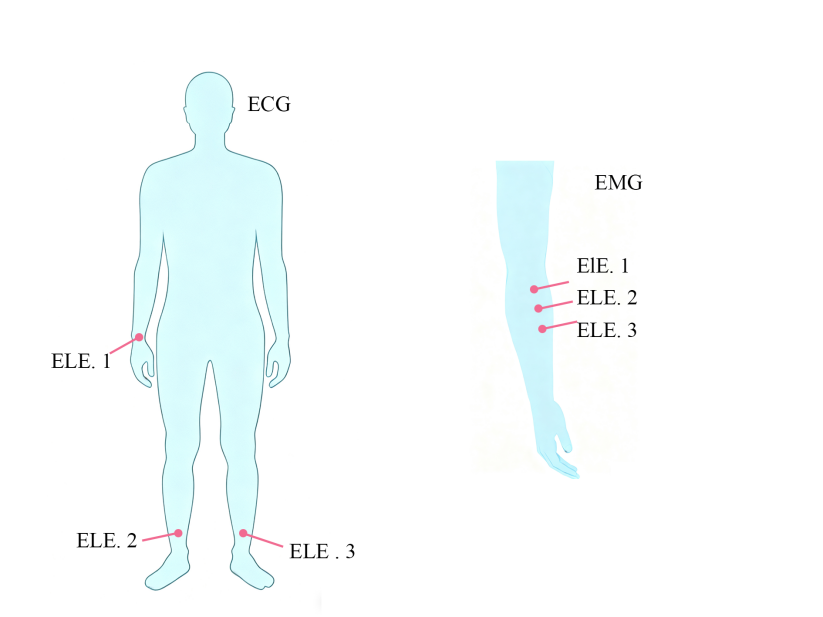


***Scheme S5*.** Schematic illustration of electrode placement position for monitoring ECG and EMG signals.

**Human Subjects Statement**: All experimental procedures involving human subjects research conducted in this study were approved by the Human Ethics Committee of Guangxi Normal University (Approval No. 20250608001). Written informed consent was obtained from all volunteers prior to their participation in motion detection and electrophysiological monitoring procedures.

1. **Supporting Figures**


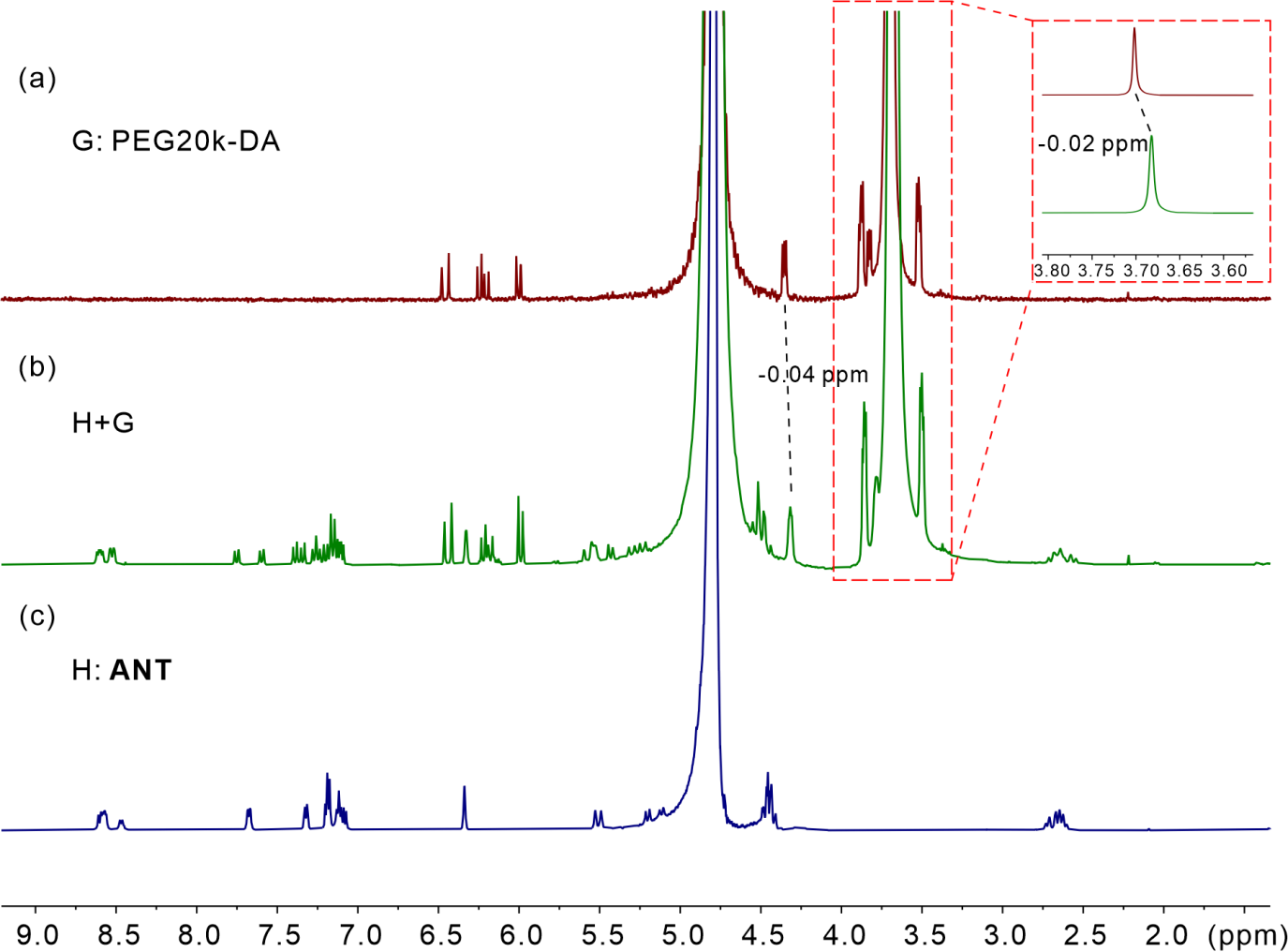


***Figure S1****.* ^1^H NMR spectra (500 MHz, D_2_O, 0.5 mM, 25 ^o^C) of (a) PEG_20k_-DA, (c) **ANT**, and (b) their equimolar mixture. The characteristic proton signals of the guest (PEG_20k_-DA) exhibit significant upfield shifts upon mixing with **ANT**, as evidenced by the comparison of spectra (a) and (b). This observation confirms that PEG_20k_-DA is threaded into the cavity of **ANT**, where its protons experience a pronounced shielding effect.


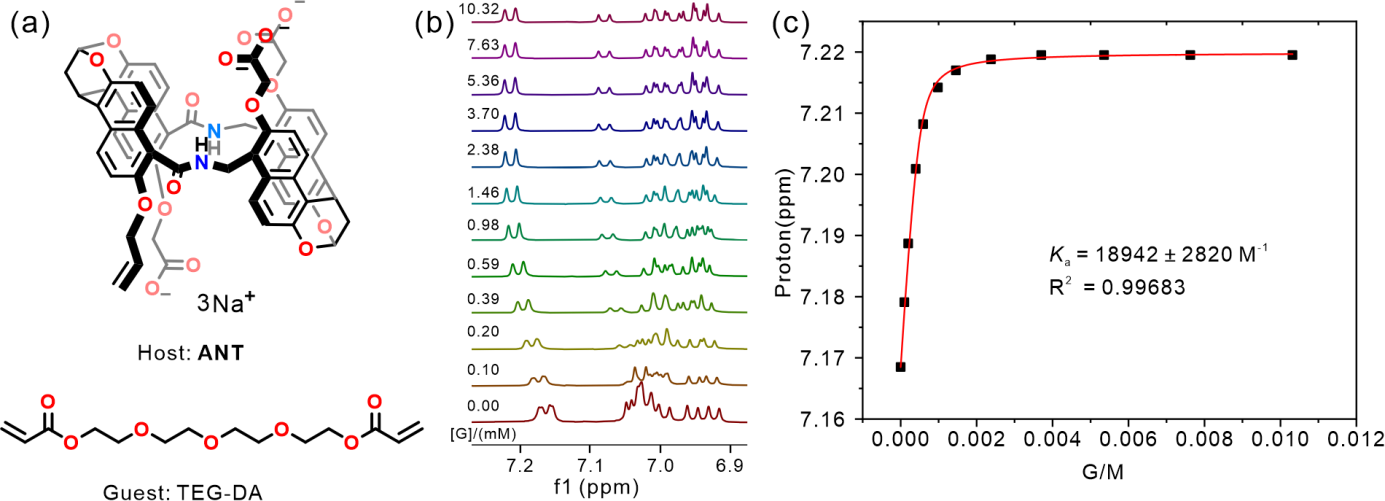


***Figure S2****.* Characterization of the host-guest interaction between **ANT** and TEG-DA. (a) Chemical structures of **ANT** and the model guest TEG-DA. (b) Partial ^1^H NMR spectra (500 MHz, D_2_O, 298 K) of **ANT** (0.5 mM) upon titration with by TEG-DA. (c) Nonlinear curve fitting of the titration data, used to determine the binding constant for the TEG-DA@**ANT** complex in D₂O at 25 °C.


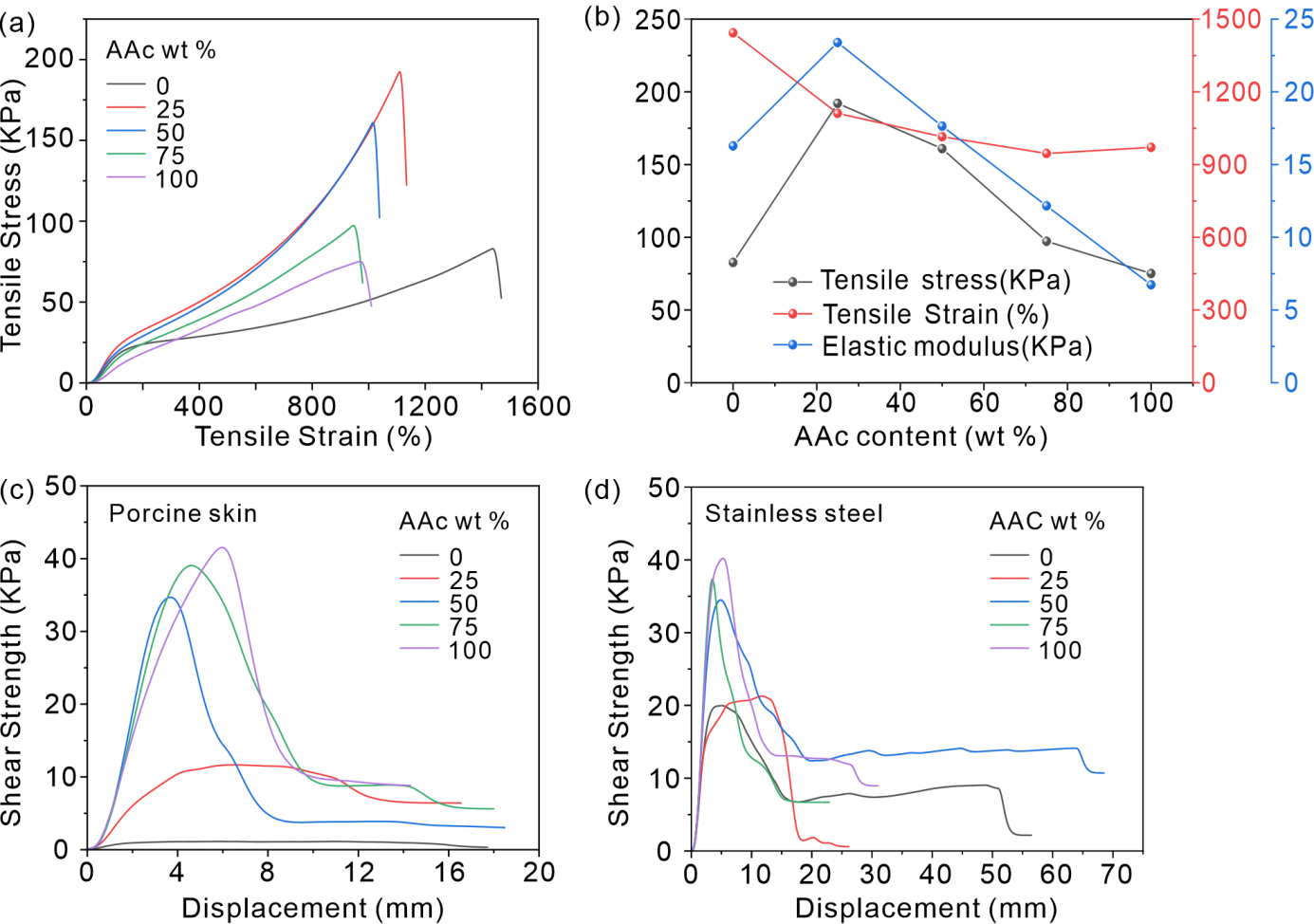


***Figure S3*.** Optimization of acrylic acid (AAc) content. (a) Mechanical properties of hydrogel **1** with varying AAc percentages. (b) Tensile strength and elongation at break as a function of AAc content. Adhesive shear strength of hydrogel **1** on fresh porcine skin (c) and stainless steel (d), plotted against AAc content.

***
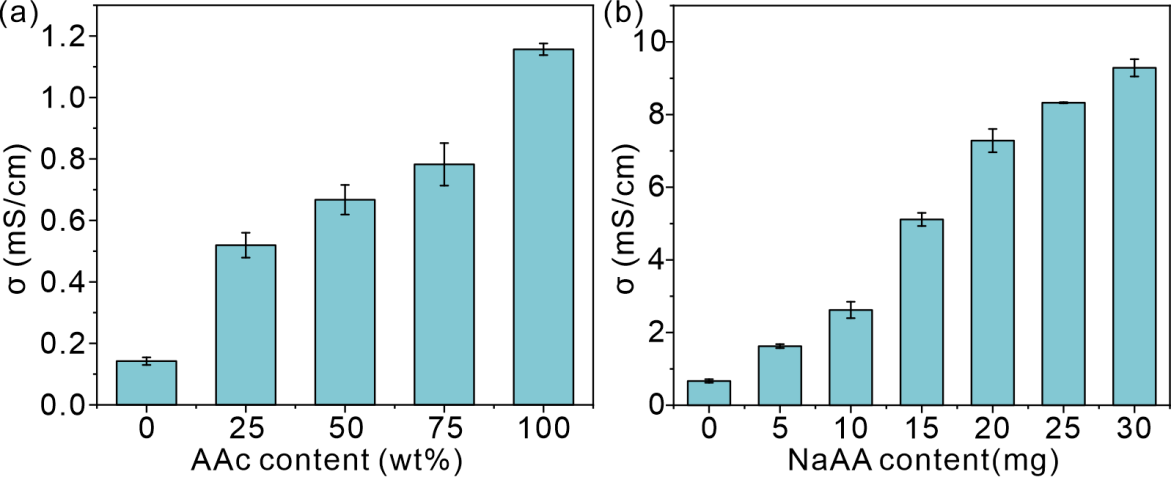
***

***Figure S4*.** The electrical conductivity of hydrogel **1** as a function of monomer content. The systematic variation in conductivity with AAc content (a) and NaAA content (b) is examined.

***
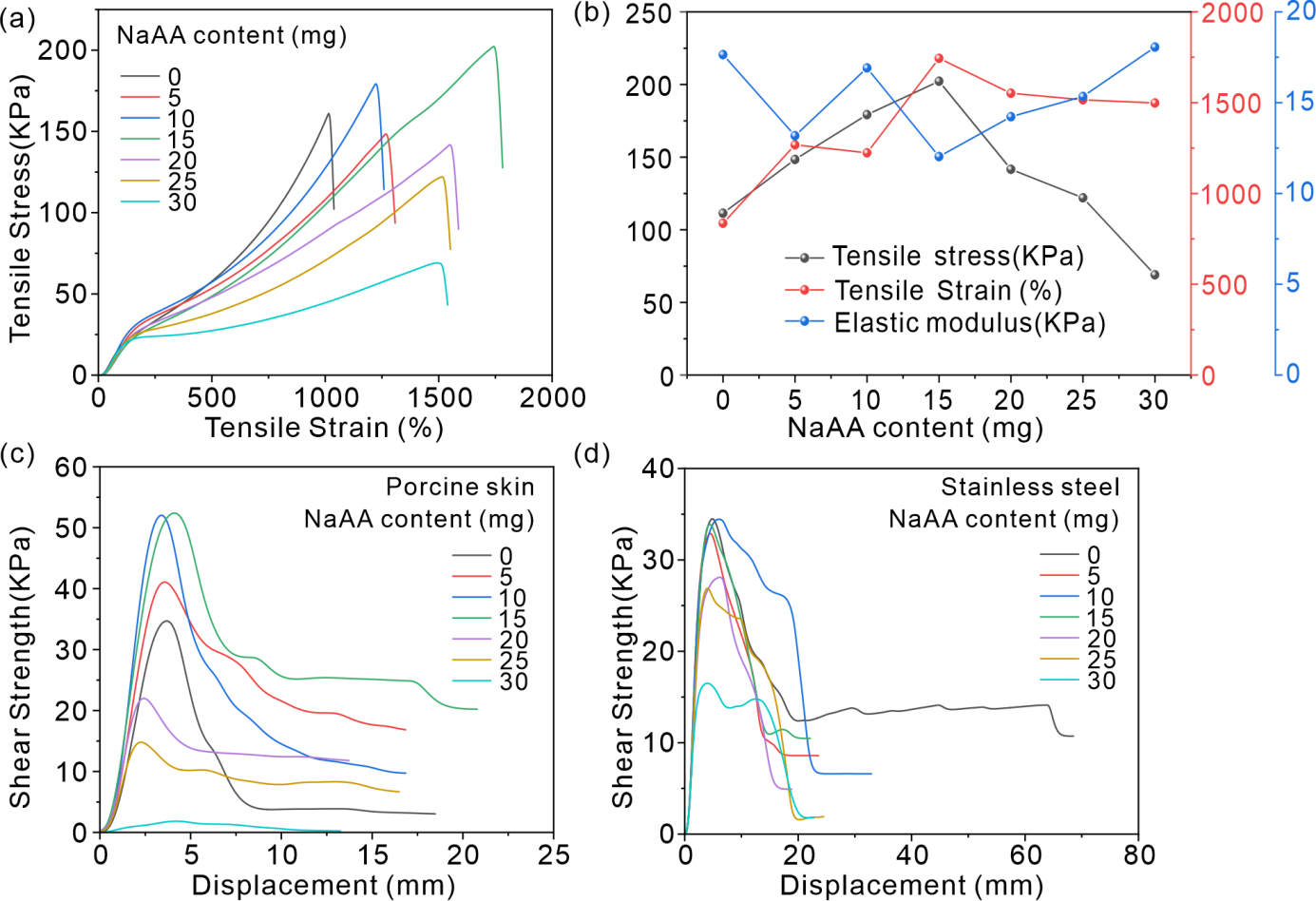
***

***Figure S5*.** Optimization of NaAA content. (a) Mechanical properties of hydrogel **1** with varying NaAA loading. (b) Tensile strength and elongation at break as a function of NaAA content. Adhesive shear strength of hydrogel **1** on fresh porcine skin (c) and stainless steel (d), plotted against AAc content.


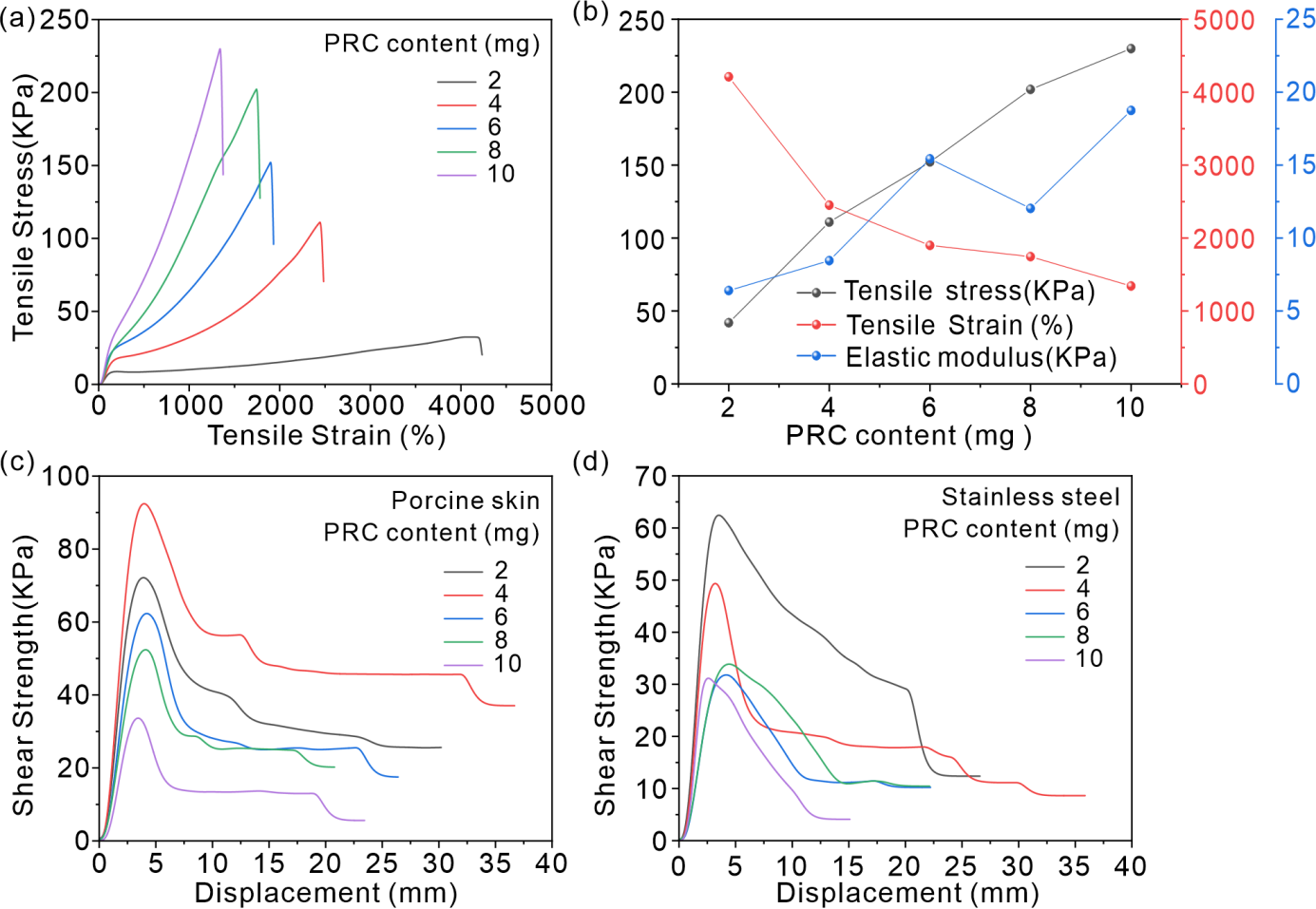


***Figure S6*.** Optimization of **PRC-ANT** crosslinker content. (a) Mechanical properties of hydrogel **1** with varying **PRC-ANT** content. (b) Tensile strength and elongation at break as a function of **PRC-ANT** content. Adhesive shear strength of hydrogel **1** onfresh porcine skin (c) and stainless steel (d) substrates versus **PRC-ANT** content.


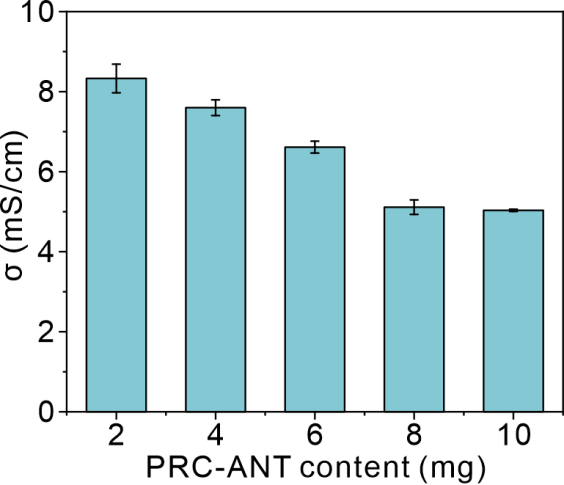


***Figure S7*.** The electrical conductivity of hydrogel **1** as a function of crosslinker content.


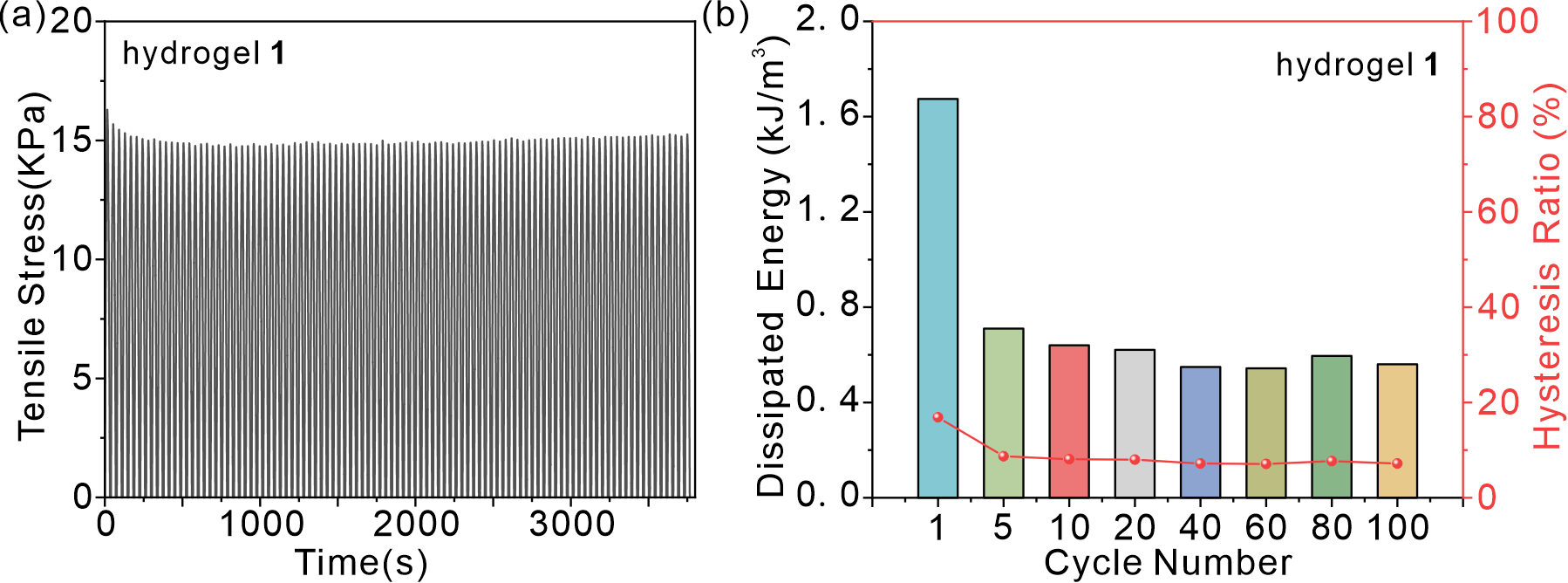


***Figure S8*.** Cyclic tensile behavior of hydrogel **1**. (a) Stress response recorded over 100 consecutive loading-unloading cycles. (b) Corresponding dissipated energy and hysteresis ratio evaluated for these cycles.


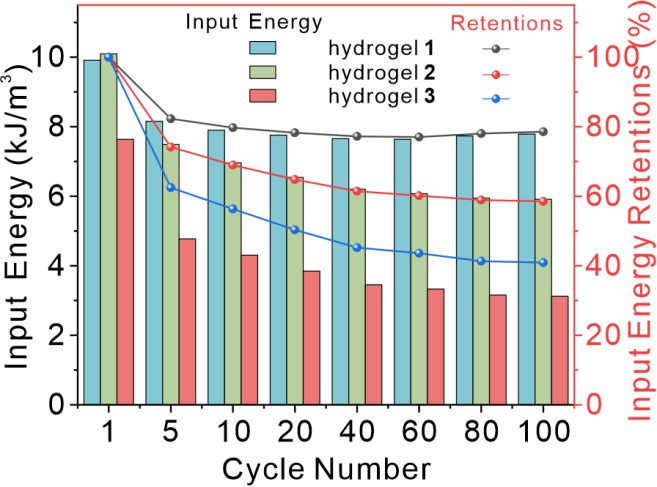


***Figure S9.*** Input energy and its retention of hydrogels **1**, **2** and **3** over 100 cycles at 100% strain.


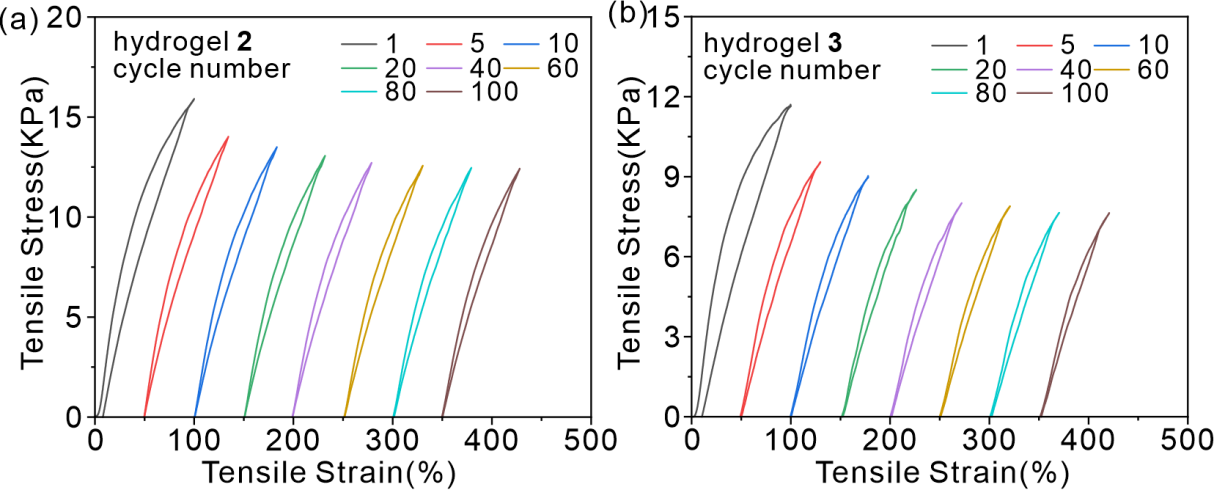


***Figure S10*.** Consecutive loading-unloading tests of the control hydrogels. (a) Representative tensile responses of hydrogel **2** over 100 cycles at 100% strain. (b) Representative tensile responses of hydrogel **3** over 100 cycles at 100% strain. For comparative purposes, the curves are offset horizontally for visual clarity.


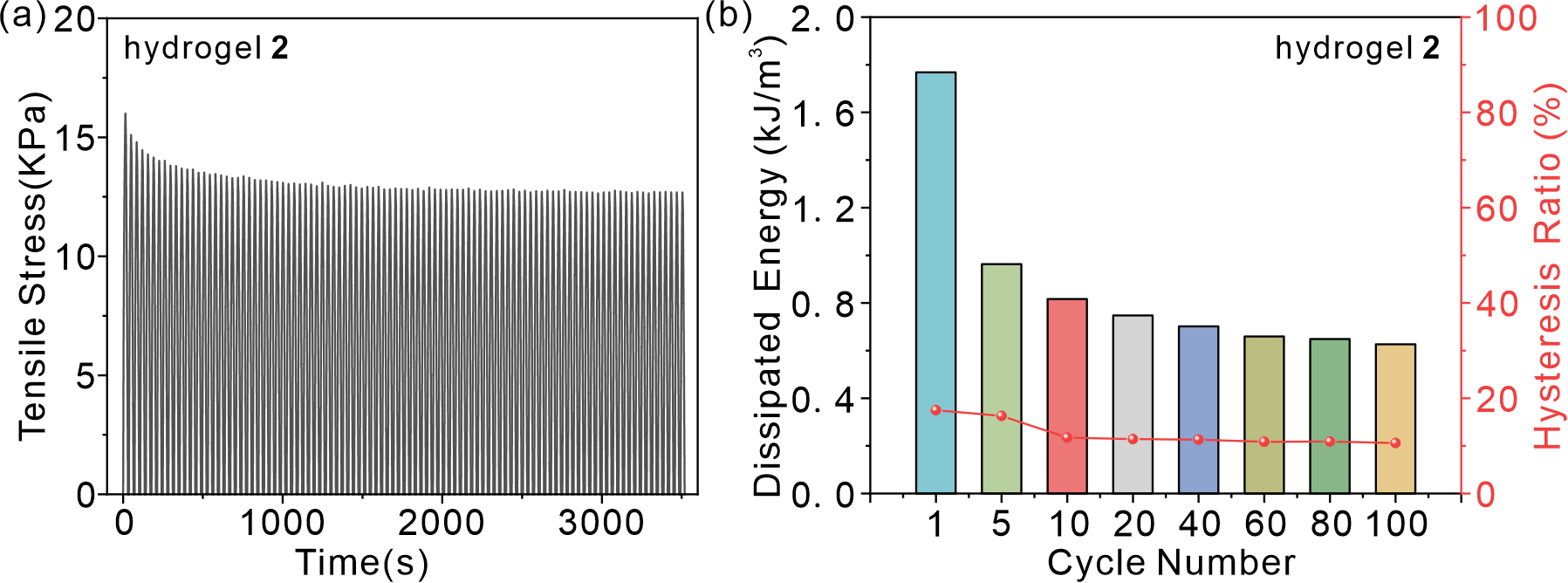


***Figure S11*.** Cyclic tensile performance of hydrogel **2**. (a) Stress response recorded over 100 consecutive loading-unloading cycles. (b) Corresponding dissipated energy and hysteresis ratio evaluated for these cycles.


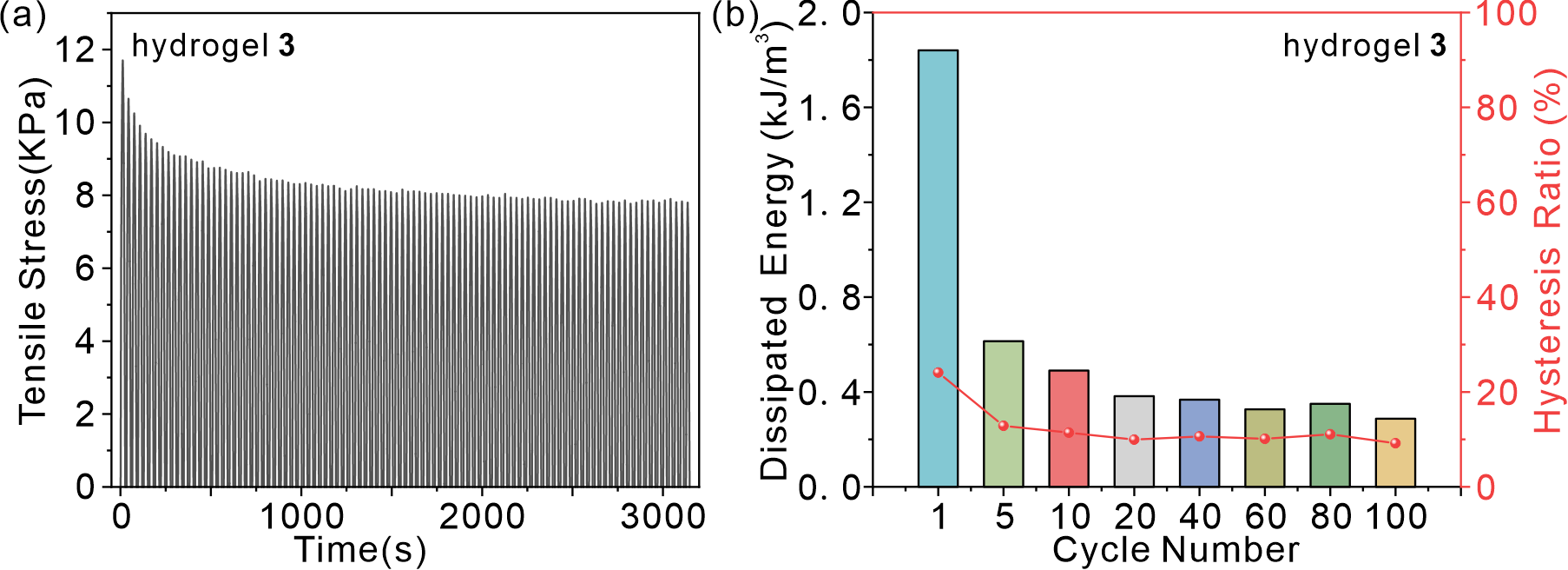


***Figure S12*.** Cyclic tensile performance of hydrogel **3**. (a) Stress response recorded over 100 consecutive loading-unloading cycles. (b) Corresponding dissipated energy and hysteresis ratio evaluated for these cycles.


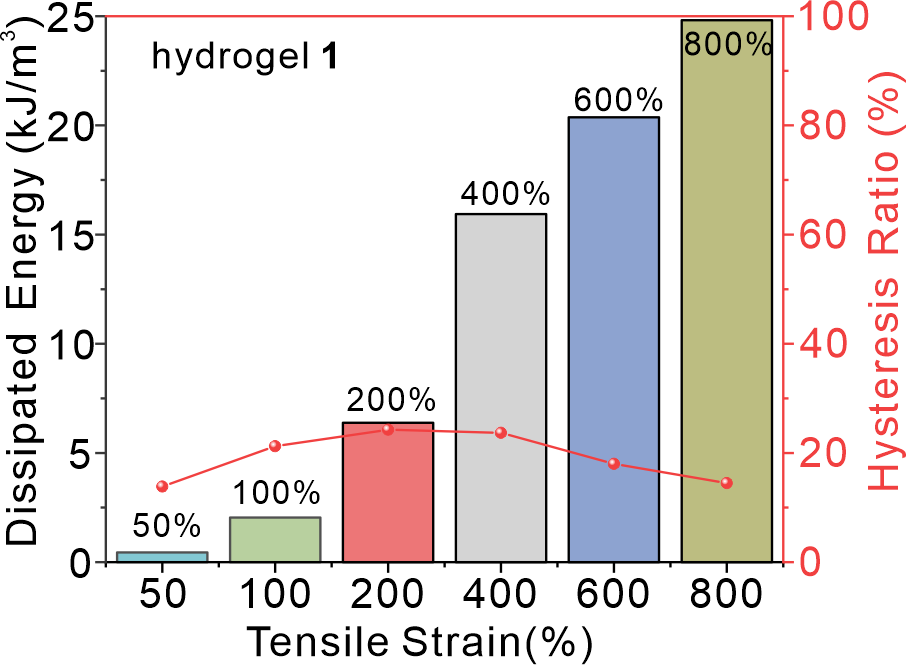


***Figure S13*.** Corresponding dissipated energy and hysteresis ratio of hydrogel **1** at different strains from 50% to 800%.


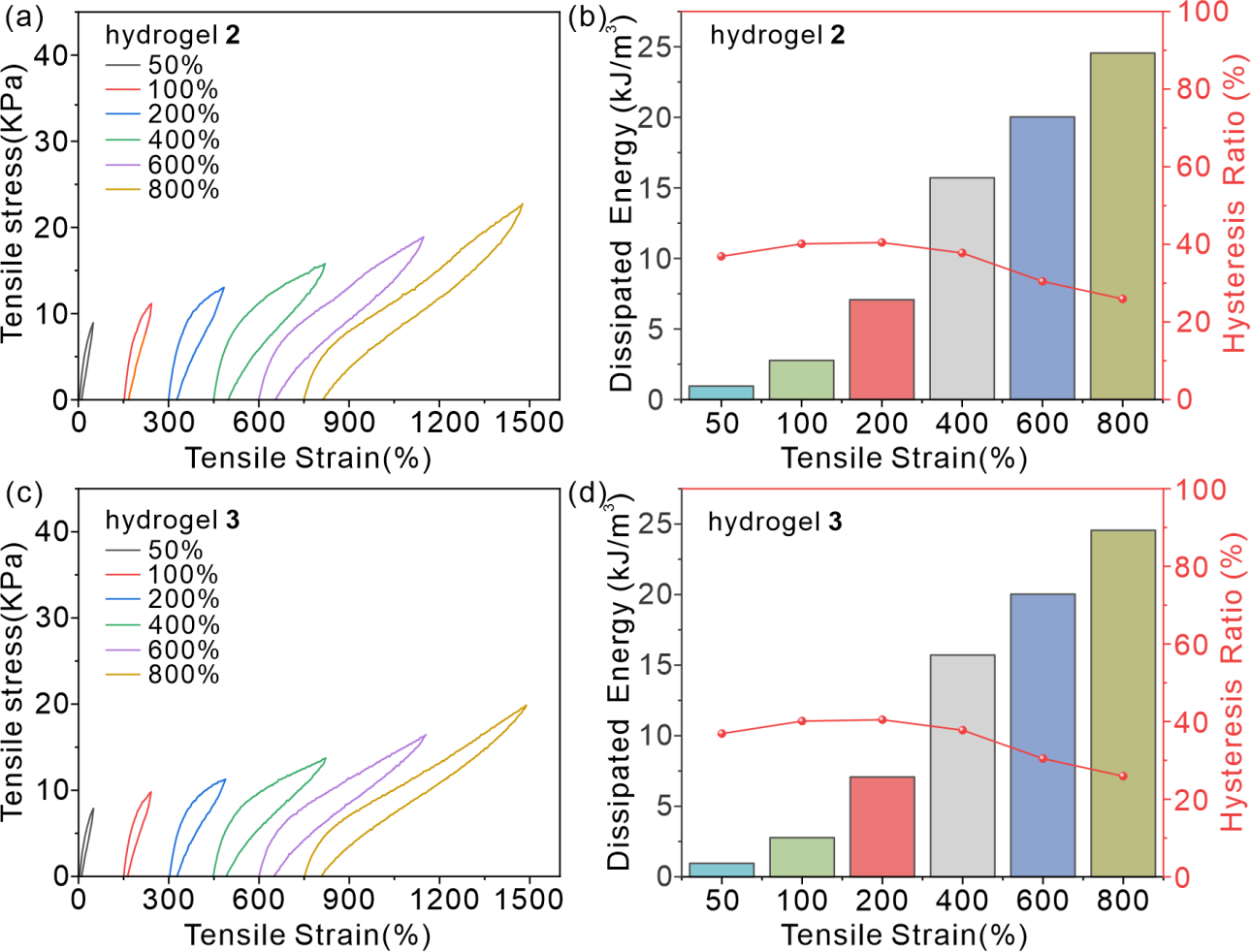


***Figure S14*.** Loading-unloading tensile curves and corresponding dissipated energy and hysteresis ratio of hydrogel **2** (a, b) and **3** (c, d) at different strains (50%~800%).


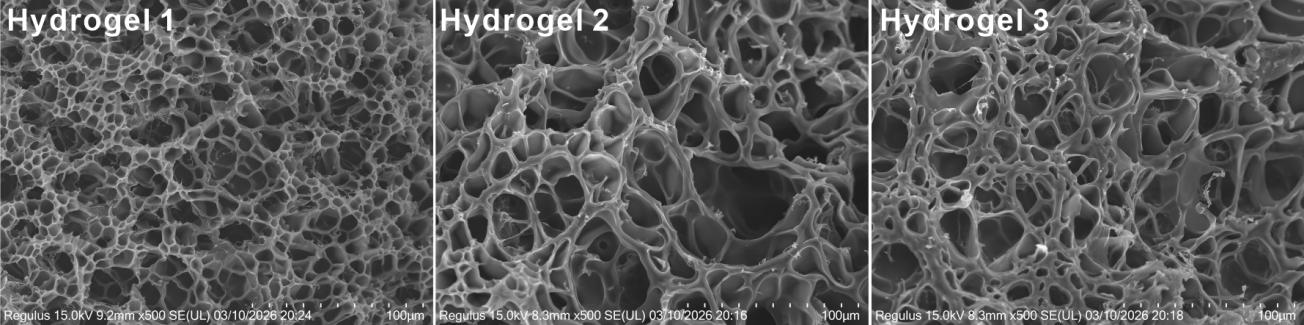


***Figure S15.*** SEM images of the freeze-dried hydrogels **1**, **2** and **3**.


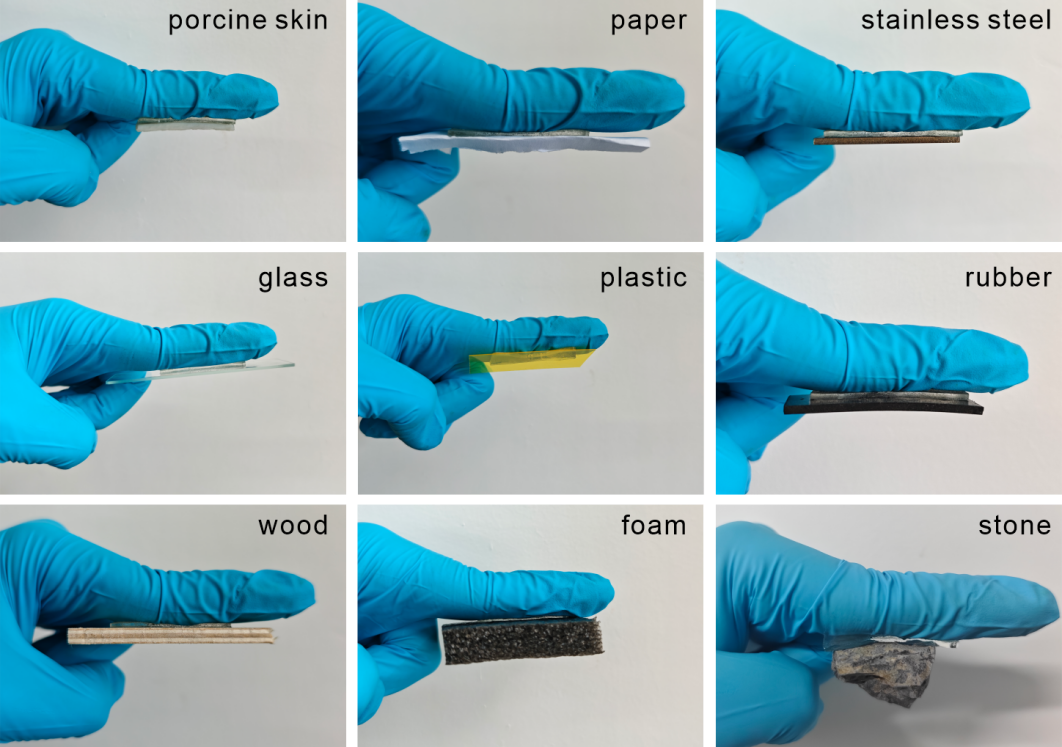


***Figure S16*.** Adhesion demonstration of hydrogel **1** on various substrates.


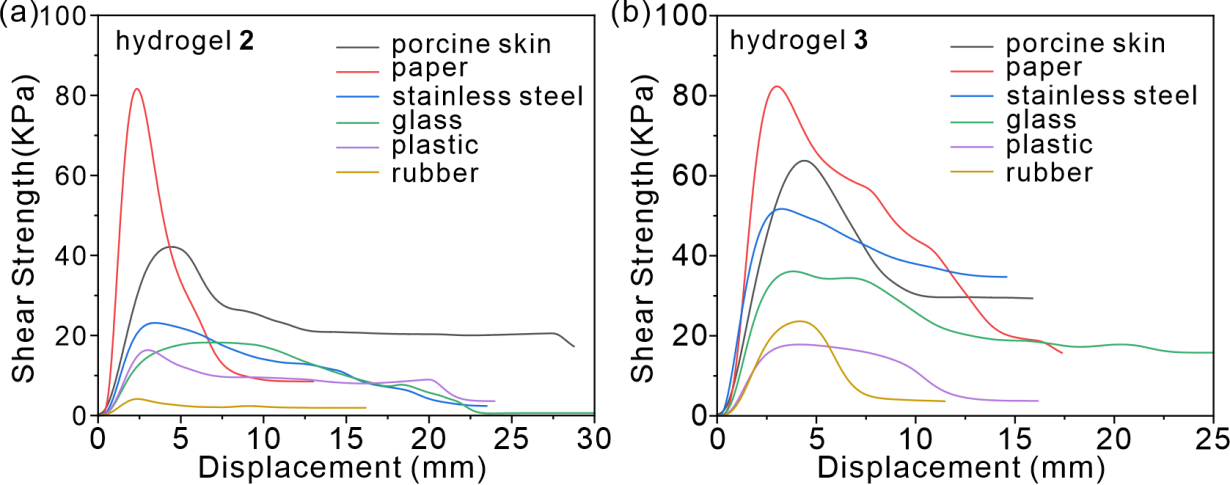


***Figure S17*.** Comparative evaluation of the adhesive shear strength for control hydrogels. The adhesion of hydrogel **2** (a) andhydrogel **3** (b) to various substrates is measured.

***
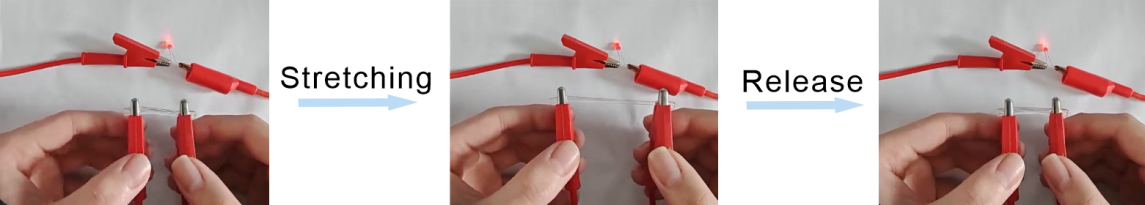
***

***Figure S18*.** Visual demonstration of the hydrogel's strain-dependent conductivity. Photographs of an LED circuit show the changes in brightness corresponding to the stretching and release of the hydrogel.


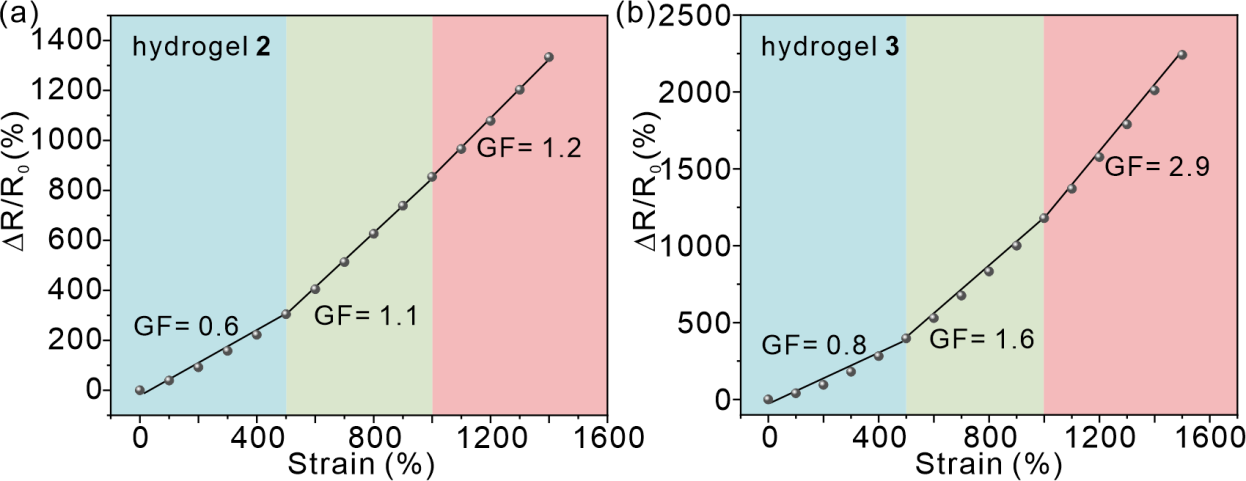


***Figure S19*.** The strain-dependent relative resistance change (ΔR/R₀) of the control hydrogels. Plots show ΔR/R₀ versus applied strain for (a) hydrogel **2** and (b) hydrogel **3**. Gauge factors for different strain ranges are indicated, respectively.


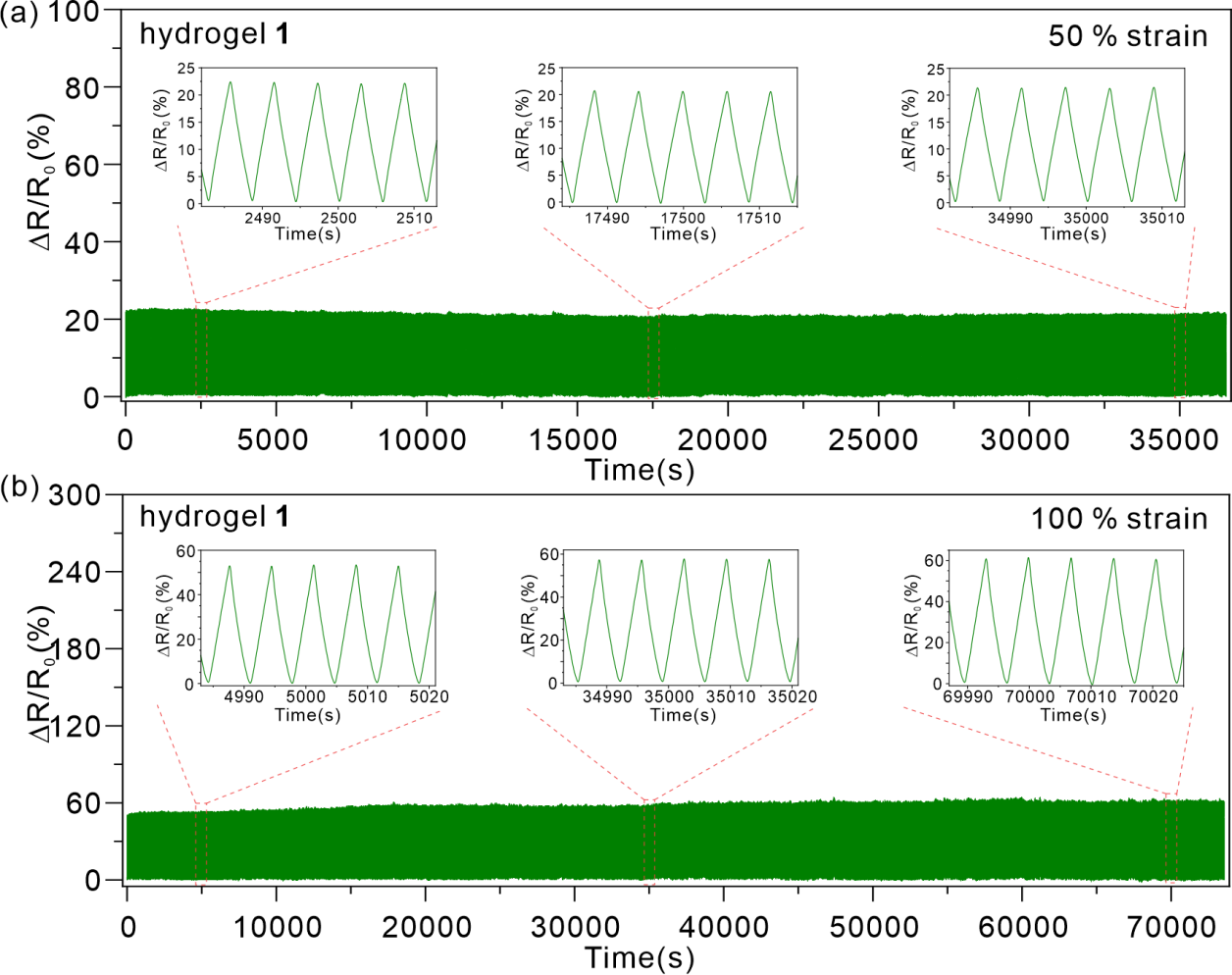


***Figure 20.*** Long-term sensing stability of hydrogel **1.** The performance stability was evaluated over 1000 consecutive loading-unloading cycles at 50% (a) and 100% (b) strain.


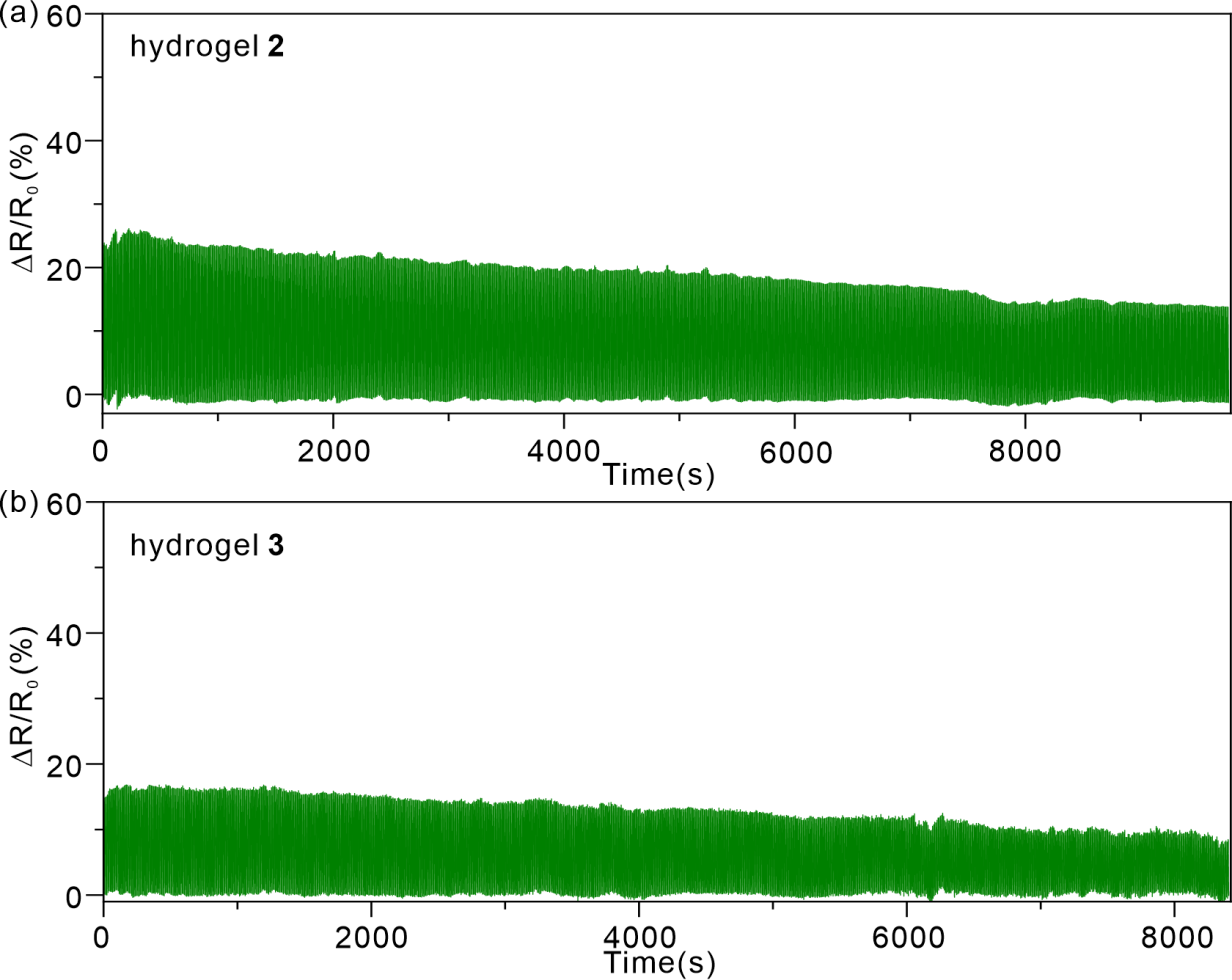


***Figure S21*.** Long-term sensing reliability of the control hydrogels. The performance stability was evaluated over 1000 consecutive loading–unloading cycles at 50% strain for hydrogel **2** (a) and hydrogel **3** (b).


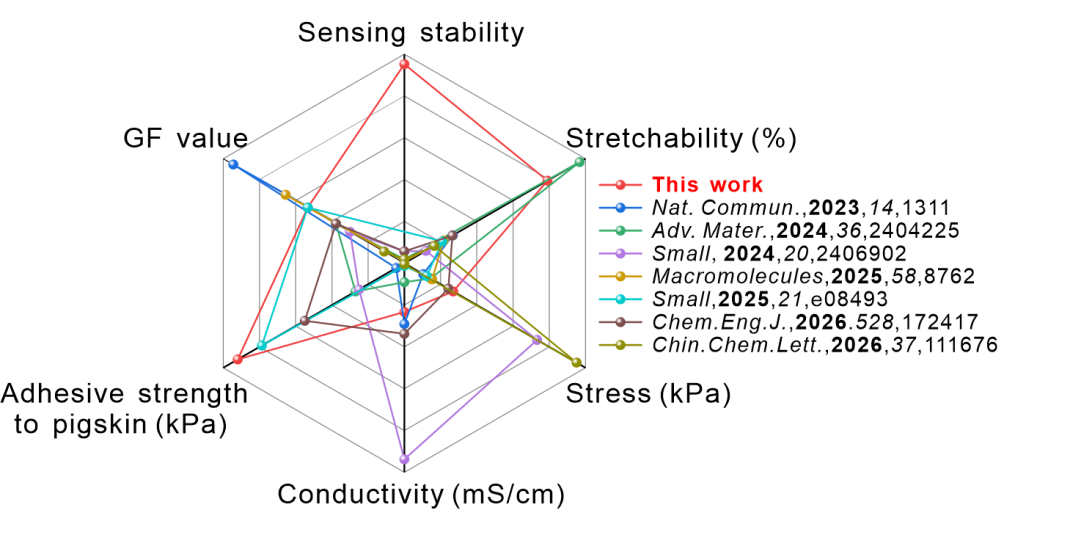


***Figure S22***. The radar plot of the key parameters relevant to wearable sensing applications for comparing hydrogel **1** with representative slide-ring sensors from the literature.


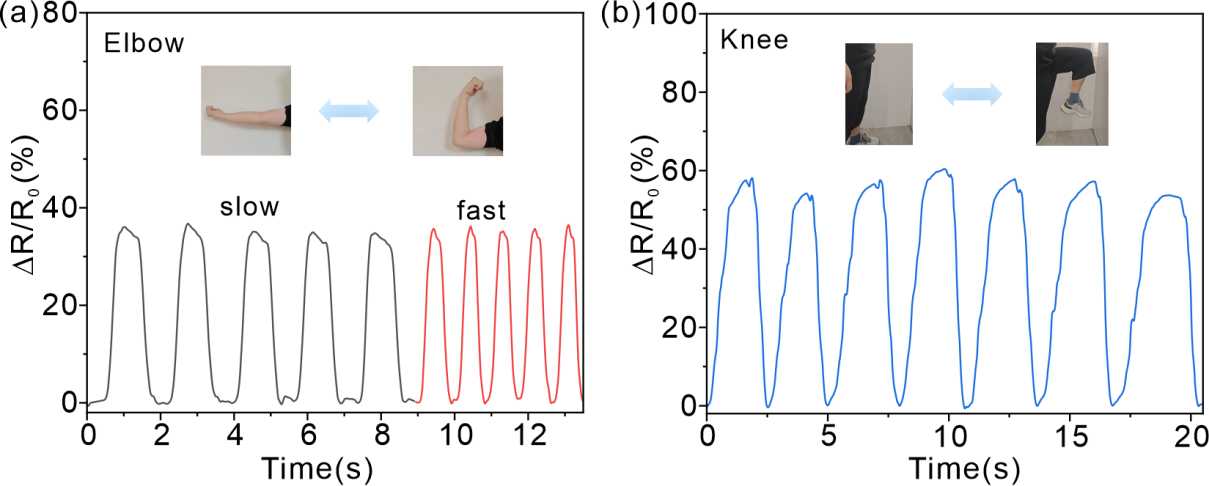


***Figure S23*.** Real-time monitoring of large joint movements. The relative resistance change (∆R/R₀) of the hydrogel **1** sensor is recorded during elbow (a) and knee (b) bending motions.


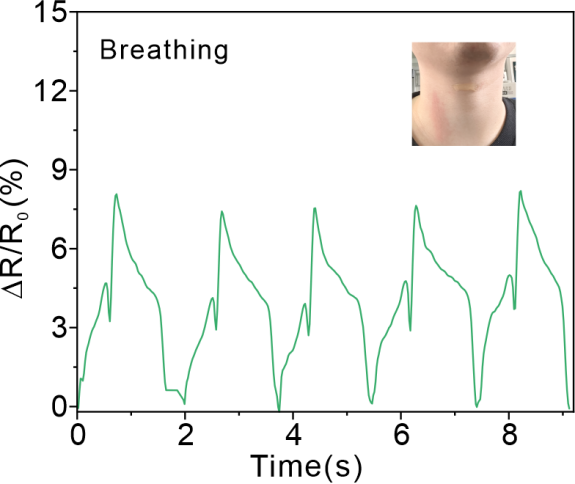


***Figure S24*.** Real-time monitoring of subtle physiological activities. The relative resistance change (∆R/R₀) of the hydrogel **1** sensor is plotted corresponding to breathing cycles.


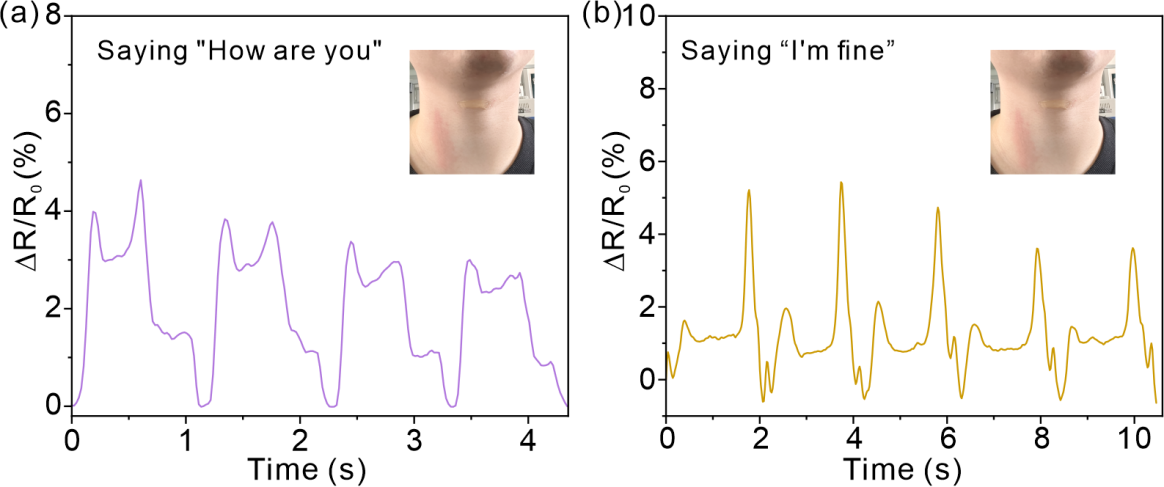


***Figure S25*.** Real-time detection of speech patterns. The relative resistance change (∆R/R₀) of the hydrogel **1** sensor is recorded upon articulation of the phrases “How are you?” (a) and “I’m fine” (b).


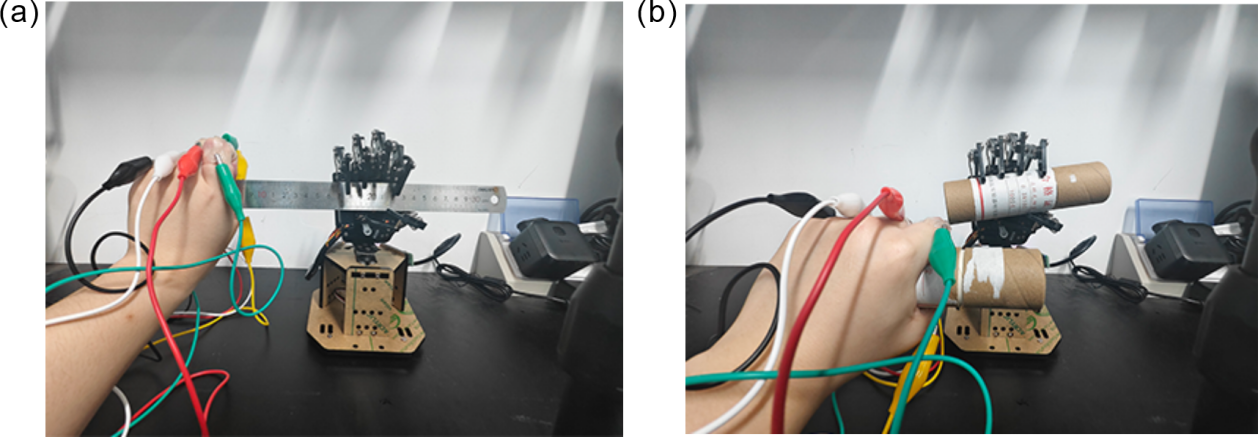


***Figure S26*.** Demonstration of robotic manipulation using the hydrogel sensor. Photographs show the control of a robotic arm for grasping a ruler (a) and a cylindrical capillary box (b).


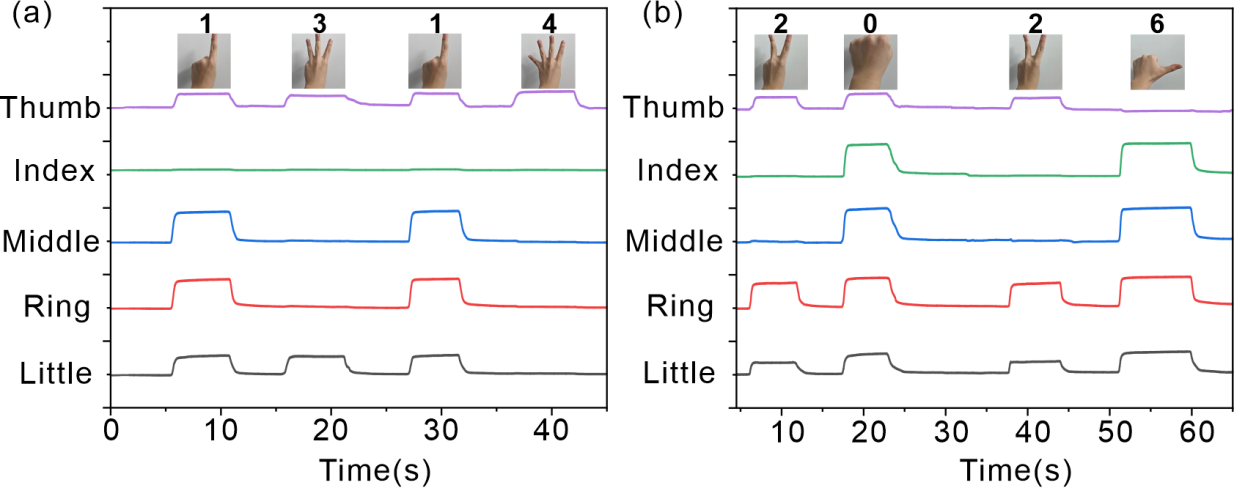


***Figure S27*.** Real-time recognition of finger-posture gestures. The relative change in electrical signal (ΔR/R_0_) is plotted in response to sequential gestures representing the numbers “**1314**” (a) and “**2026**” (b).


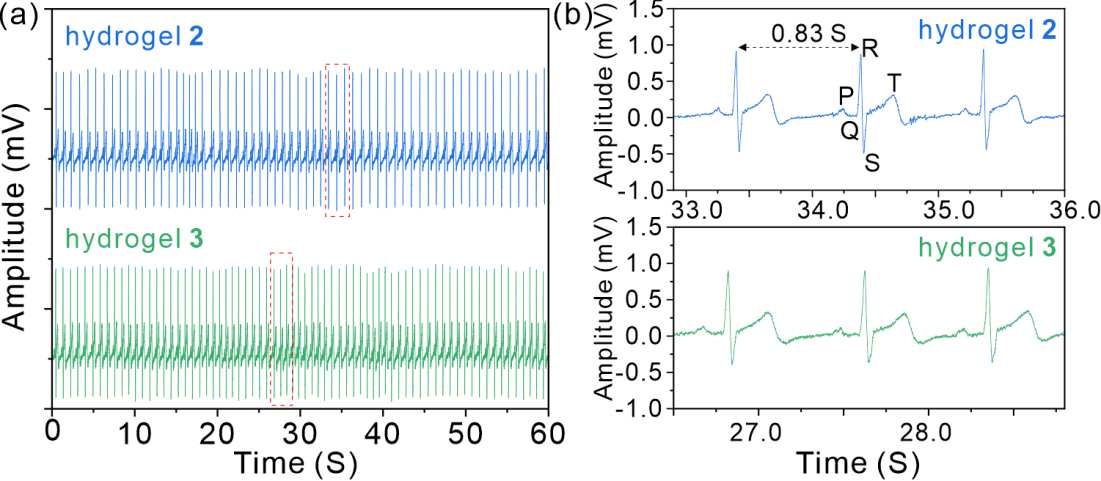


***Figure S28*.** Comparative analysis of ECG signals. (a) Representative electrocardiogram (ECG) waveforms recorded by hydrogel **2** and **3**. (b) Enlarged view of characteristic P‑QRS‑T complexes from (a).


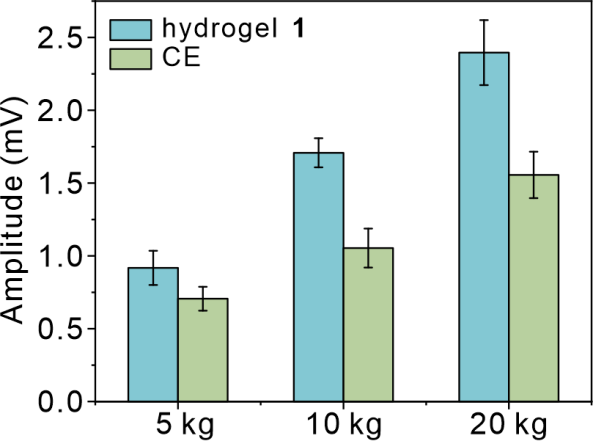


***Figure S29*.** Comparative measurements of electromyographic (EMG) signals. The EMG amplitude is shown for different grip forces (5, 10, and 20 kg), acquired simultaneously using the hydrogel **1** sensor and a conventional electrode (CE).


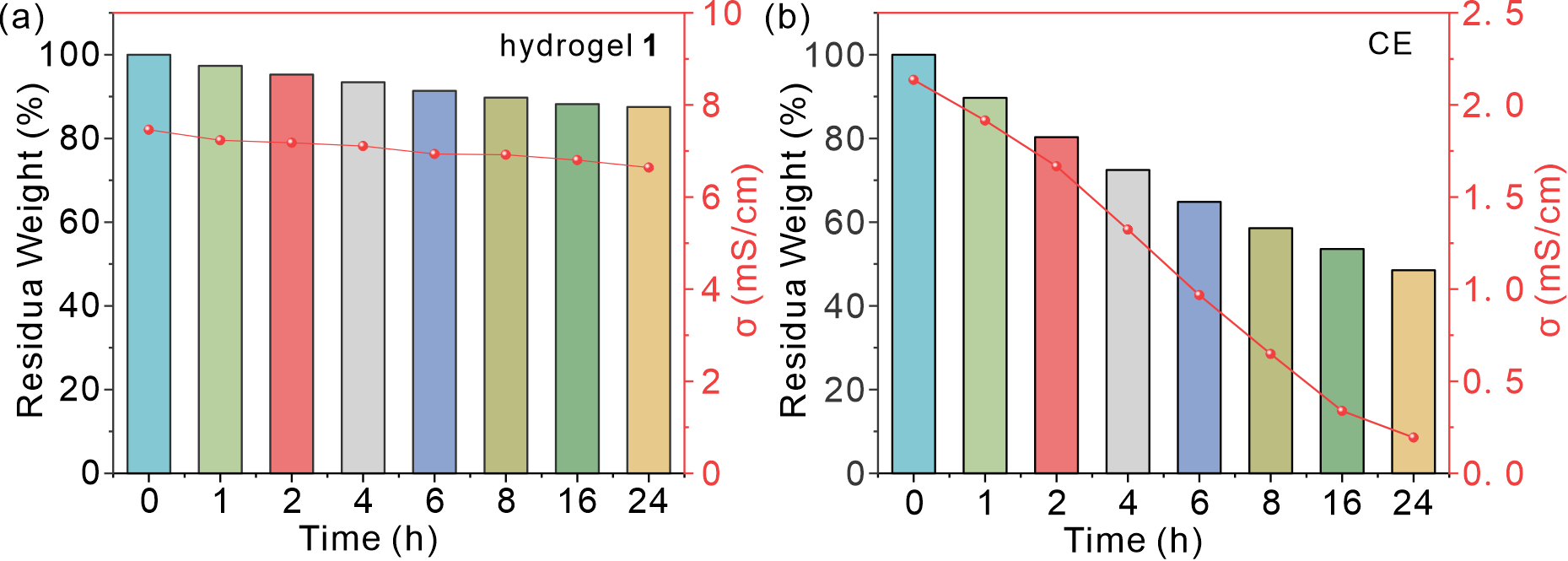


***Figure S30.*** Water retention and conductivity stability of hydrogel **1** (a) and commercial electrode (CE) (b).


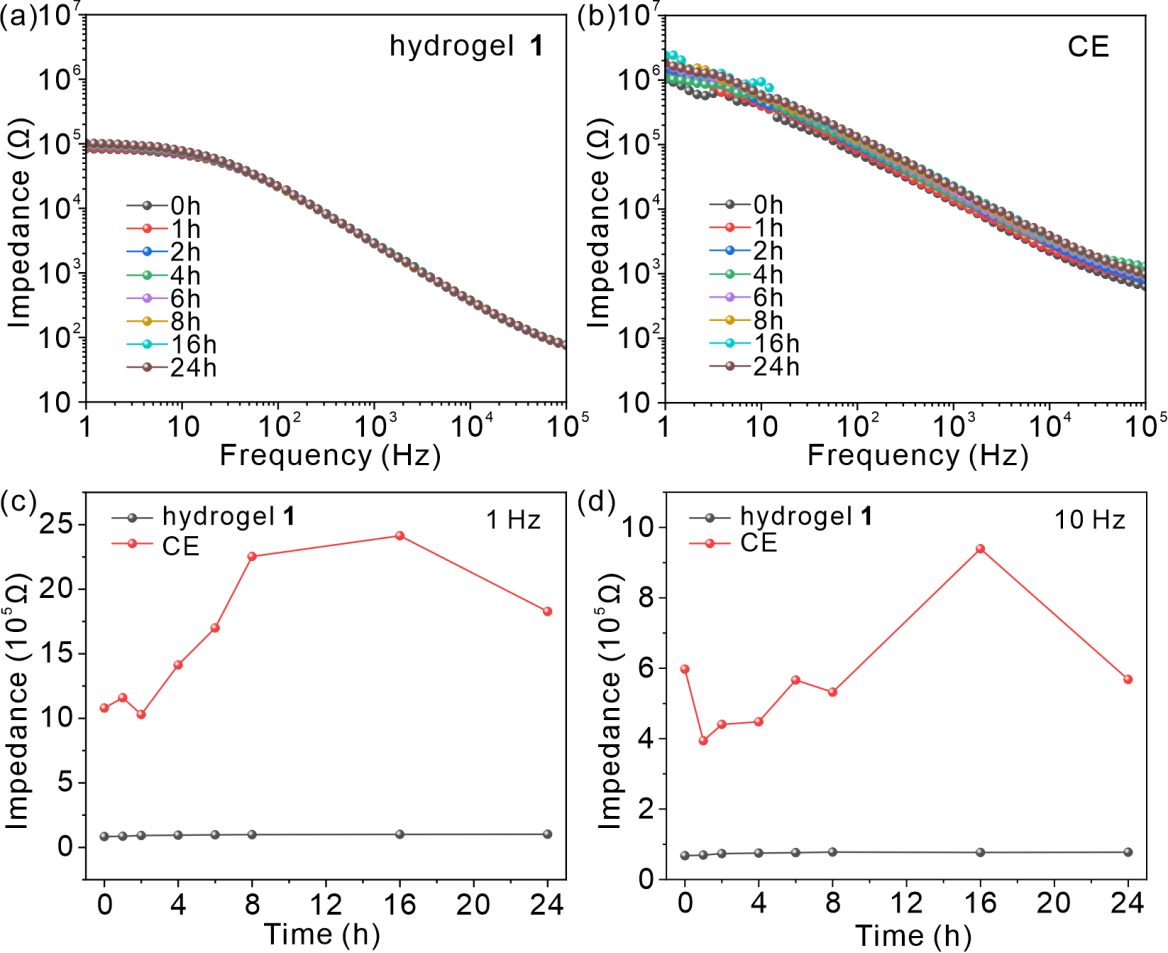


***Figure S31.*** Bode plots of electrode-skin interface impedance for hydrogel **1** (a)and CE (b) over 24 h. The changes of the electrode-skin interface impedance at 1 Hz (c) and 10 Hz (d).


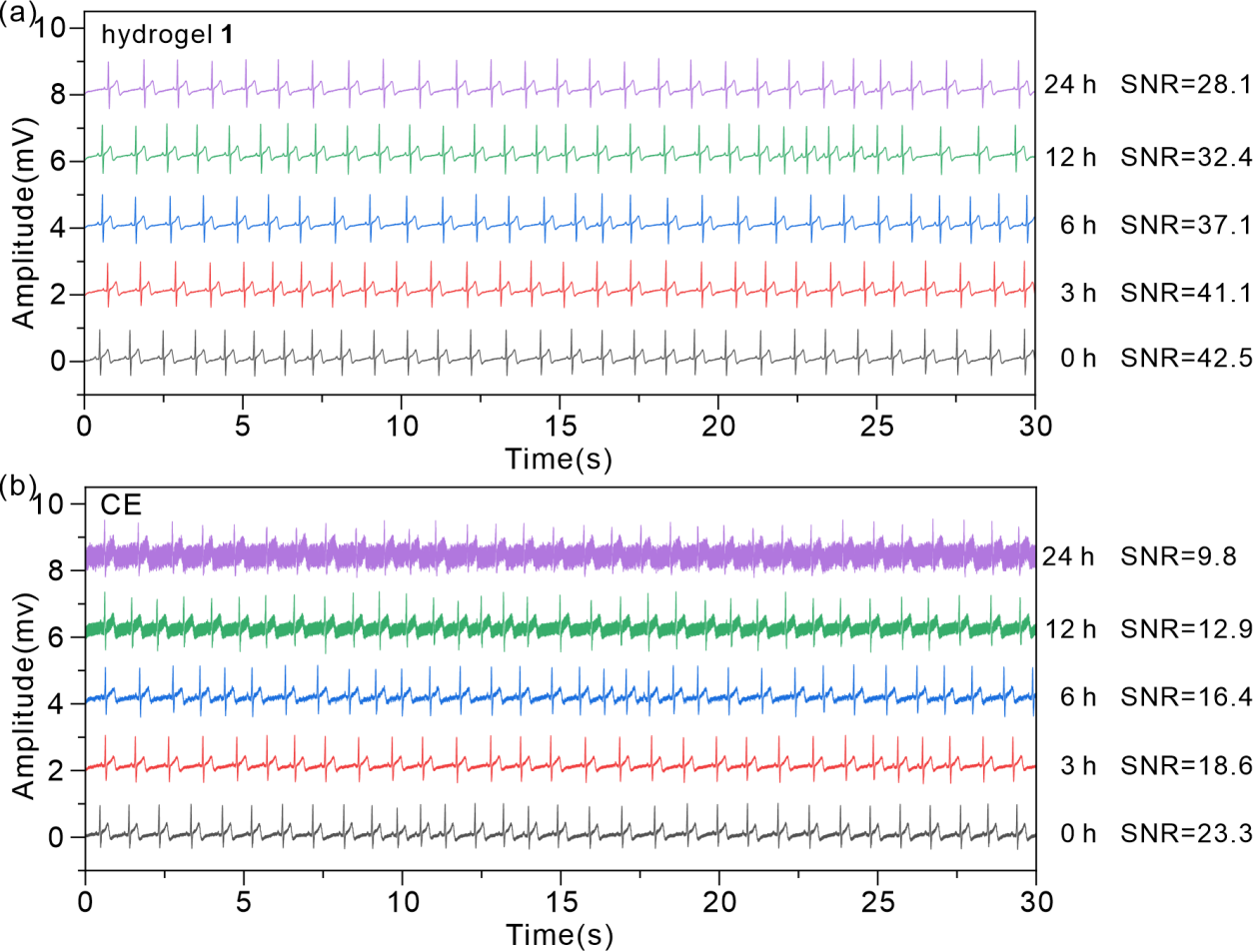


***Figure S32.*** The electrocardiogram (ECG) pattern and SNR obtained using hydrogel **1** (a) and CE (b) over 24 h.

**Table S3** Comparison of key parameters between hydrogel **1** and representative slide-ring hydrogel sensors

|  | Stretchability  (%) | Stress  (kPa) | Conductivity  (mS/cm) | Adhesive strength  to pigskin (kPa) | GF value | Sensing stability tests |
| --- | --- | --- | --- | --- | --- | --- |
| **This work** | **2450** | **110** | **7.46** | **92** | **4.8** | **10000 cycles** |
| *Nat. Commun.*, **2023**, *14*, 1311 | 830 | 78.1 | 9.3 | 4.5 | 8.5 | 100 cycles |
| *Adv. Mater.*, **2024**, *36*, 2404225 | 3000 | 109 | 2.9 | 27 | 3.4 | 200 |
| *Small,* **2024**, *20*, 2406902 | 369 | 550 | 30 | 25 | 2.7 | < 600 cycles |
| *Macromolecules,* **2025**, *58*, 8762 | 674 | 115 | - | - | 5.9 | 100 cycles |
| *Small,* **2025**, *21*, 2501558 | 2572 | 449 | 6.4 |  | 6.1 | 300 cycles |
| *Small,* **2025**, *21*, 2412665 | 2000 | 154 | 22 | 5.8 (chicken skin) | 1.0 | - |
| *Small,* **2025**, *21*, e08493 | 651 | 92.14 | 0.4 | 78.7 | 4.8 | - |
| *Chem. Eng. J.*, **2026**, *528*, 172417 | 823 | 182 | 10.8 | 55 | 3.4 | < 600 cycles |
| *Chin. Chem. Lett.*, **2026**, *37*, 111676 | 513 | 713 | 0.15 | - | < 1 | 200 cycles |

1. **References**

[1] G.-B. Huang, S.-H. Wang, H. Ke, L.-P. Yang, W. Jiang, Selective Recogonition of Highly Hydrophilic Molecules in Water by Endo-Functionalized Molecular Tubes. *J. Am. Chem. Soc.,* **2016**, *138*, 14550-14553.

[2] H. Yao, Y.-M. Wang, M. Quan, M. U. Farooq, L.-P. Yang, W. Jiang, Adsorptive Separation of Benzene, Cyclohexene, and Cyclohexane by Amorphous Nonporous Amide Naphthotube Solids. *Angew. Chem. Int. Ed.,* **2020**, *59*, 19945-19950; *Angew. Chem.,* **2020**, *132*, 20117-20122.

[3] H. Ke, L.-P. Yang, M. Xie, Z. Chen, H. Yao, W. Jiang, Shear-induced Assembly of a Transient yet Highly Stretchable Hydrogel based on Pseudopolyrotaxanes. *Nat. Chem.*, **2019**, *11*, 470-477.

[4] G. M. Sheldrick, Crystal Structure Refinement with SHELXL. *Acta Cryst.* **2015**, *C71*, 3**–**8.

[5] O. V. Dolomanov, L. J. Bourhis, R. J. Gildea, J. A. K. Howard, H. Puschmann, OLEX2: A Complete Structure Solution, Refinement and Analysis Program *J. Appl. Cryst.* **2009**, *42*, 339**–**341.

[6] A. L. Spek, A Tool for the Calculation of the Disordered Solvent Contribution to the Calculated Structure Factors. *Acta Crystallorgr.* **2015**, *C71*, 9**–**18.

[7] H. Zhao, S. Wang, T. Li, Y. Liu, Y. Tang, Z. Zhang, H. Li, Y. Li, X. Li, G. Li, X. Liu, Q. Tian, Z. Liu, Stretchable Multi-Channel Ionotronic Electrodes for In Situ Dual-Modal Monitoring of Muscle–Vascular Activity. *Adv. Func. Mater.*, **2024**, *34*, 2308686.

[8] X. Yan, R. Zhao, H. Lin, Z. Zhao, S. Song, Y. Wang, Nucleobase-Driven Wearable Ionogel Electronics for Long-Term Human Motion Detection and Electrophysiological Signal Monitoring. *Adv. Func. Mater.*, **2025**, *35*, 2422244.

[9] J. Luo, C. Sun, B. Chang, B. Zhang, K. Li, Y. Li, Q. Zhang, H. Wang, C. Hou, On-Skin Paintable Water-Resistant Biohydrogel for Wearable Bioelectronics. *Adv. Func. Mater.*, **2024**, *34*, 2400884.
